# Metallodrugs against Breast Cancer: Combining the Tamoxifen Vector with Platinum(II) and Palladium(II) Complexes

**DOI:** 10.3390/pharmaceutics15020682

**Published:** 2023-02-17

**Authors:** Aleksandr Kazimir, Benedikt Schwarze, Peter Lönnecke, Sanja Jelača, Sanja Mijatović, Danijela Maksimović-Ivanić, Evamarie Hey-Hawkins

**Affiliations:** 1 Institute of Inorganic Chemistry, Faculty of Chemistry and Mineralogy, Leipzig University, 04103 Leipzig, Germany; 2 Institute for Medical Physics and Biophysics, Medical Faculty, Leipzig University, 04107 Leipzig, Germany; 3 Department of Immunology, Institute for Biological Research “Siniša Stanković”, National Institute of Republic of Serbia, University of Belgrade, 11060 Belgrade, Serbia

**Keywords:** breast cancer, tamoxifen derivative, platinum dichloride, palladium dichloride, platinacarboranes, palladacarboranes, oxidative stress, cytotoxicity

## Abstract

The luminal A-subtype of breast cancer, where the oestrogen receptor α (ERα) is overexpressed, is the most frequent one. The prodrug tamoxifen (**1**) is the clinically used agent, inhibiting the ERα activity via the formation of several active metabolites, such as 4-hydroxytamoxifen (**2**) or 4,4′-dihydroxytamoxifen (**3**). In this study, we present the tamoxifen derivative 4-[1,1-bis(4-methoxyphenyl)but-1-en-2-yl]-2,2′-bipyridine (**4**), which was combined with platinum or palladium dichloride, the former a well-known scaffold in anticancer treatment, to give [PtCl_2_(**4**-κ^2^*N*,*N*′)] (**5**) or [PdCl_2_(**4**-κ^2^*N*,*N*′] (**6**). To prevent fast exchange of weakly coordinating chlorido ligands in aqueous solution, a bulky, highly stable and hydrophobic *nido*-carborate(−2) ([C_2_B_9_H_11_]^2−^) was incorporated. The resulting complexes [3-(**4**-κ^2^*N*,*N*′)-3,1,2-PtC_2_B_9_H_11_] (**7**) and [3-(**4**-κ^2^*N*,*N*′)-3,1,2-PdC_2_B_9_H_11_] (**8**) exhibit a dramatic change in electronic and biological properties compared to **5** and **6**. Thus, **8** is highly selective for triple-negative MDA-MB-231 cells (IC_50_ = 3.7 μM, MTT test), while **7** is completely inactive against this cell line. The observed cytotoxicity of compounds **4**–**6** and **8** against this triple-negative cell line suggests off-target mechanisms rather than only ERα inhibition, for which these compounds were originally designed. Spectroscopic properties and electronic structures of the metal complexes were investigated for possible explanations of the biological activities.

## 1. Introduction

**Figure 1:**
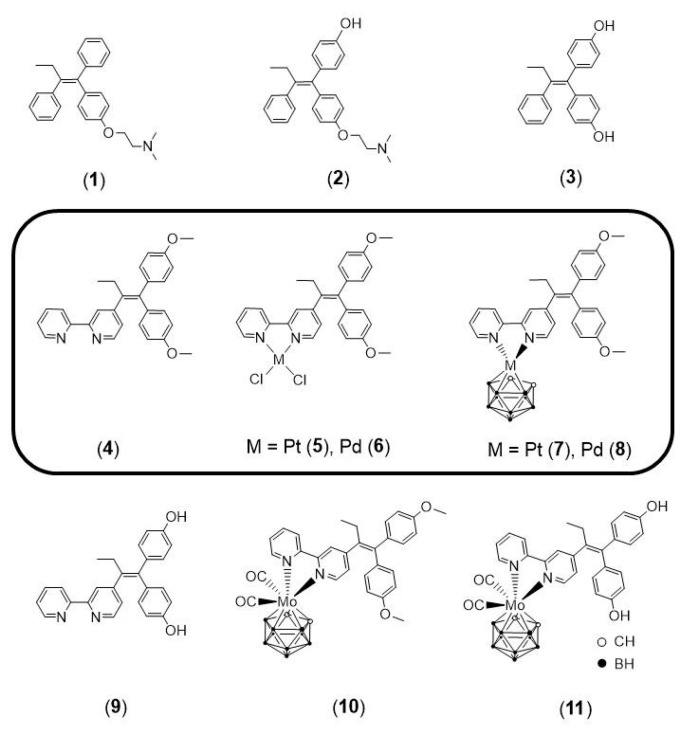
Schematic molecular structures of tamoxifen and tamoxifen-based derivatives: literature-known reference compounds tamoxifen (**1**), 4-hydroxytamoxifen (**2**), and 4,4′-dihydroxytamoxifen (**3**), compounds investigated in this study 4-[1,1-bis(4-methoxyphenyl)but-1-en-2-yl]-2,2′-bipyridine (**4**, L), [PtCl_2_(L-κ^2^*N*,*N*′)] (**5**), [PdCl_2_(L-κ^2^*N*,*N*′)] (**6**), [3-(L-κ^2^*N*,*N*′)-3,1,2-PtC_2_B_9_H_11_] (**7**), [3-(L-κ^2^N,N′)-3,1,2-PdC_2_B_9_H_11_] (**8**), as well as previously reported compounds 4-[1,1-bis(4-hydroxyphenyl)but-1-en-2-yl]-2,2′-bipyridine (**9**, L^1^), [3-(L-κ^2^*N*,*N*′)-3-(CO)_2_-3,1,2-MoC_2_B_9_H_11_] (**10**), and [3-(L^1^-κ^2^*N*,*N*′)-3-(CO)_2_-3,1,2-MoC_2_B_9_H_11_] (**11**) [22]. Compounds reported here are framed.

According to data from the Cancer Statistics Center of the American Cancer Society, in 2021 breast cancer took the first place being the most prevalent form of cancer in women [1]. Approximately 70% of the breast cancers are hormone receptor positive (HR+) [2]. In particular, the nuclear receptors for oestrogen (ERα) and/or progesterone are main transcription factors in the development of HR+ breast cancers [2,3]. In the presence of oestrogen, the nuclear ERα activates the cell division via inducing the gene transcription [4]. Overexpression of ERα is the reason of the tumour proliferation in the oestrogen receptor positive (ER+) luminal A breast cancer [5]. In this case, anti-oestrogen therapies, based on anti-oestrogen agents such as tamoxifen (Figure 1, compound **1**), are common and effective approaches [6]. Tamoxifen is a selective oestrogen receptor modulator (SERM) and a prodrug, which is metabolised in the liver by cytochrome P450 giving more than twenty metabolites [7,8]. Among these metabolites, two active species are well-known: 4-hydroxytamoxifen (**2**, Figure 1) and 4,4′-dihydroxytamoxifen (**3**, Figure 1) [9]. The structure of the ERα co-crystallised with the oestrogen antagonist 4-hydroxytamoxifen demonstrated that the metabolite occupies the ligand-binding domain (LBD), induces conformational changes of the receptor helices, and thus leads to inhibition of the ERα functions [10]. Although hormone therapy showed its efficiency in the treatment of HR+ breast cancers, a significant number of patients developed resistance towards anti-oestrogen therapy with tamoxifen, which is known to be related to ERα-independent mechanisms [11].

Tamoxifen resistance is becoming increasingly problematic as a concomitant side effect of hormone therapy [12,13]. Therefore, it is necessary to design novel therapeutics. A known promising way towards overcoming adverse side effects involves the combination of inhibitors and metallodrugs, which potentially have greater advantages in the adjuvant therapy creating several therapeutic effects [14,15].

Due to the antagonistic and cytotoxic properties of tamoxifen, its chemical structure remains attractive as a lead structure for synthetic modifications [16,17,18,19]. Tamoxifen has been modified in order to combine this structure with transition metals such as rhenium [16], ruthenium [16], titanium [17], osmium [18], and platinum [19]. Some other prominent examples include a combination of the tamoxifen structure with ferrocene (ferrocifen) [20] or with carborane, namely boroxifen [21]. We have already reported 4-[1,1-bis(4-methoxyphenyl)but-1-en-2-yl]-2,2′-bipyridine (**4**) incorporating the chelating 2,2′-bipyridine (2,2′-bpy) unit (Figure 1) [22], which enables the formation of inhibitor–metallodrug conjugates with a variety of transition metal complexes. Examples are the molybdacarboranes **10** and **11** (Figure 1) which have shown promising anticancer activity [22].

A number of examples for 2,2′-bpy transition metal complexes with very interesting activity profiles against different cancer cell lines including triple-negative breast cancer (TNBC) have already been reported [18,19,20,21,23,24,25,26,27,28,29,30]. For instance, ruthenium(II) compounds incorporating bisphosphino and 2,2′-bpy ligands exhibited cytotoxicity towards HR+ and TNBC cell lines [24]. Polydentate phenylquinoline and 2,2′-bpy-bearing iridum(III) and rhodium(III) complexes demonstrated their high potency towards TNBC cells via inhibition of cyclin-dependent kinase 9 (CDK9) [25]. Moreover, polydentate rhodium(III)-2,2′-bpy complexes have shown selectivity towards lysine-specific demethylase 5A (KDM5A), which is a promising target in the treatment of TNBC [26]. Another example is the pioneer study for the present investigations on a molybdacarborane complex bearing a 2,2′-bpy ligand, which exhibited higher cytotoxic activity when pre-incubated with bovine serum albumin (BSA) [27]. The mechanism of action against breast cancer stem cells and their potential clinical application have been considered for many other transition metal-2,2′-bpy complexes, including molybdenum, ruthenium, copper, nickel, cobalt, and osmium [28]. Interestingly, the design of the polydentate 2,2′-bpy scaffold and its conjugation to amino acids, lipids, carbohydrates, or vitamins allows it to regulate the (high) toxicity [29]. Rhenium(I) tricarbonyl aqua 2,2′-bpy complexes exhibited activity against the HR+ MCF-7 cell line [30]. Additionally, rhenium(I), ruthenium(II), osmium(II), and platinum(II) compounds with nitrogen-based bidentate and polydentate ligands demonstrated anticancer activity in vitro induced by visible light, making them potentially applicable in photodynamic therapy (PDT) [31,32,33]. Another class of organometallic compounds, where the unsubstituted phenyl ring of tamoxifen was replaced by ferrocene (ferrocifen), demonstrated its efficiency against various breast cancer cell lines due to the redox-active iron cation of the ferrocene moiety including structural rearrangements (Fenton chemistry) [20].

Among all metallodrugs, platinum(II) complexes (e.g., cisplatin, carboplatin, oxaliplatin) are the most investigated anticancer agents [34]. Many studies examine the mechanisms of action of these drugs; one of them is the ability of the platinum compounds to form DNA adducts preventing the repair of DNA [34,35]. However, proteins also contain functional groups in their amino acid side chains suitable for coordination of metal cations, thus leading to DNA-independent mechanisms of action [36]. Furthermore, cisplatin and carboplatin generate reactive oxygen species (ROS) inducing oxidative stress in cells and ultimately leading to cell death [37]. Regarding breast cancer therapy, platinum drugs are especially active against TNBC [38]. However, their application is often limited due to severe side effects related to systemic toxicity of these compounds and development of resistance against platinum-based chemotherapy [39]. To overcome some limitation connected with platinum, palladium has been considered as a promising alternative due to the similar coordination behaviour [40,41]. However, the hydrolysis of palladium(II) chloride analogues is too rapid and 10^5^ times faster than for the corresponding platinum(II) complexes, thus preventing the formed reactive palladium(II) species to reach their pharmacological target. Accordingly, *cis*-[PdCl_2_(NH_3_)_2_] did not exhibit anticancer activity [42]. However, as the in vitro and in vivo activity and stability of palladium(II) complexes depend on the coordination strength and exchange rate of (labile) ligands, the appropriate design of biologically active carrier ligands can make Pd^2+^ complexes even more favourable than the known Pt^2+^ drugs (e.g., polyamine complexes [43,44]). Thus, several nitrogen-containing polydentate palladium(II) complexes were shown to exhibit cytotoxicity against TNBC via similar mechanisms as platinum(II)-based compounds, including interaction with DNA [23,45]. Furthermore, several studies have demonstrated that complexes of *cis*- and also *trans*-palladium dichloride had equal, or even higher, cytotoxic activities compared to their platinum analogues [39,40,41].

Besides the successful examples of metallodrugs mentioned above, also half-, mixed- and full-sandwich metallacarboranes have proven their potential for medical application [46,47]. The hydrophobic features of the *nido*-dicarborate ion ([C_2_B_9_H_11_]^2−^, dicarbollide), the possible resistance towards enzymatic degradation [48,49], and facile three-dimensional modifiability for improving target engagement [48] make metallacarboranes attractive for further research as anticancer agents. Promising examples of half- and full-sandwich complexes are molybdacarboranes [22] and ruthenacarboranes [50,51] with cytotoxic activity against both HR+ and TNBC cell lines. The activity of the molybdacarboranes exceeded not only the one of the active metabolite of tamoxifen (**3**) but also showed different modes of action [22] compared to ferrocifen.

We here report on the extension of our previous results obtained for **9**–**11** [22] to known bioactive transition metal moieties, namely PtCl_2_ (**5**), PdCl_2_ (**6**), 3,1,2-PtC_2_B_9_H_11_ (**7**), and 3,1,2-PdC_2_B_9_H_11_ (**8**) complexes with the tamoxifen derivative **4**, and present their syntheses, spectroscopic and theoretical/computational characterisation followed by an assessment of their cytotoxic activity in in vitro cell cultures.

## 2. Materials and Methods

### 2.1. Methods

All reactions were carried out under a dry and oxygen-free nitrogen or argon atmosphere using Schlenk line technique. Methanol was dried over CaH_2_ and kept over molecular sieves (3 Å). Tetrahydrofuran (THF) was dried over potassium with benzophenone and kept over molecular sieves 4 Å [52]. Dichloromethane (DCM), diethyl ether (Et_2_O), and *n*-hexane were used as purchased. The molecular sieves (3 and 4 Å) were activated under vacuum at 300 °C for 3 h. Silica gel for the column was purchased from Merck (0.035–0.070 mm, 60 Å). Thin layer chromatography (TLC) was conducted on pre-coated TLC sheets ALUGRAM Xtra SIL G/UV254 (0.20 mm silica gel 60 F254); visualisation of the compounds on the plate was achieved with UV light (254 and 366 nm). Starting materials [PtCl_2_(DMSO)_2_] and Tl_2_C_2_B_9_H_11_ were synthesised according to the literature [53,54]. Chemicals were used as purchased: thallium(I) acetate and K_2_[PtCl_4_] from Sigma Aldrich and PdCl_2_ from TCI Chemicals. Handling and all procedures involving thallium(I) compounds were carried out as stated by the safety data sheet and according to the general synthetic procedures involving thallium-based compounds [55]. The reference compound 1,1-bis(4-hydroxyphenyl)-2-phenylbut-1-ene (**3**) was synthesised according to the literature procedure [56]. Spectroscopic and analytical data for full characterisation of compounds **4**–**8** are given in the Supplementary Materials, Characterisation.

### 2.2. Instrumentation

NMR spectra were recorded at room temperature (25 °C) with a Bruker AVANCE III HD 400 spectrometer. ^1^H (400.13 MHz) and ^13^C (100.16 MHz) NMR spectra were referred to SiMe_4_ (TMS) as an internal standard. ^11^B NMR (128.38 MHz) spectra were referenced to the unified Ξ scale [57]. ESI mass spectra were recorded with a Bruker ESQUIRE 3000 (Benchtop LC Ion trap) mass spectrometer. The FT-IR spectra were obtained with a Nicolette IS5 (ATR) from Thermo Fisher (Waltham, MA, USA) with the scan range 4000–400 cm^−1^. A Hereaus VARIO EL oven was used to perform elemental analyses.

### 2.3. Syntheses

#### 2.3.1. Synthesis of 4-[1,1-bis(4-methoxyphenyl)but-1-en-2-yl]-2,2′-bipyridine (**4**, L)

The ligand 4-[1,1-bis(4-methoxyphenyl)but-1-en-2-yl]-2,2′-bipyridine was prepared according to the published procedure [22]. Analytical data were in agreement with those previously reported.

#### 2.3.2. Synthesis of [PtCl_2_(L-κ^2^*N,N′*)] (**5**)

The ligand **4** (152 mg, 0.36 mmol, 1 eq.) and [PtCl_2_(DMSO)_2_] (151 mg, 0.36 mmol, 1 eq.) were mixed in dry THF (15 mL). The reaction mixture was heated to reflux and stirred overnight. A colour change was observed from a white suspension to an orange solution. The solution was cooled to ambient temperature, and the solvent was evaporated under reduced pressure. The orange residue was washed with Et_2_O (10 mL × 3) and cold methanol (10 mL) resulting in an orange powder (206 mg, 0.30 mmol, 83%). Crystals of **5** suitable for single crystal X-ray crystallography were obtained by layering a DCM solution of compound **5** with *n*-pentane over a period of 14 days (*n*-pentane/DCM 3:2, *v*:*v*).

#### 2.3.3. Synthesis of [PdCl_2_(L-κ^2^*N,N′*)] (**6**)

The ligand **4** (63 mg, 0.15 mmol, 1 eq.) and PdCl_2_ (27 mg, 0.15 mmol, 1 eq.) were dissolved in methanol (15 mL). The mixture was stirred overnight at ambient temperature. The yellow precipitate was filtered off and dissolved in a small amount of DCM. This DCM solution was washed with water (15 mL × 2) and brine (15 mL × 2). The solvent was evaporated under reduced pressure, and the yellow residue was dried under high vacuum giving **6** as a yellow powder (63 mg, 0.11 mmol, 70%). Crystals of **6** suitable for single crystal X-ray crystallography were obtained by layering a DCM solution of compound **6** with *n*-pentane over a period of 15 days (*n*-pentane/DCM 3:2, *v*:*v*).

#### 2.3.4. Synthesis of [3-(L-κ^2^*N*,*N′*)-3,1,2-PtC_2_B_9_H_11_] (**7**)

The platinum(II) complex **5** (100 mg, 0.15 mmol, 1 eq.) and solid Tl_2_C_2_B_9_H_11_ (80 mg, 0.15 mmol, 1 eq.) were placed in a Schlenk flask, and dry THF (15 mL) was added. The mixture turned immediately dark red upon addition of the solvent. The reaction mixture was stirred overnight at ambient temperature. Then, stirring was stopped allowing the formed TlCl to precipitate. The solution was transferred to another Schlenk flask via cannula, and the solvent was evaporated under vacuum from the filtrate. The black residue was dissolved in minimal amounts of DCM and purified by column chromatography on silica gel. The elution with DCM/*n*-hexane (5:1, *v*:*v*) resulted in four fractions, of which the third red fraction contained the product. The solvent was evaporated under reduced pressure giving **7** as a dark red powder (45 mg, 0.06 mmol, 40%). Crystals of **7** suitable for single crystal X-ray crystallography were obtained by slow evaporation of a DCM solution of compound **7**.

#### 2.3.5. Synthesis of [3-(L-κ^2^*N*,*N′*)-3,1,2-PdC_2_B_9_H_11_] (**8**)

Compound **8** was synthesised according to the procedure described above for compound **7**. The palladium(II) complex **6** (100 mg, 0.17 mmol, 1 eq.) and Tl_2_C_2_B9H_11_ (93 mg, 0.17 mmol, 1 eq.) reacted in dry THF (15 mL) overnight. The formed precipitate of TlCl was filtered off, and the solvent was evaporated under reduced pressure. The elution of the brown residue with DCM/*n*-hexane (3:1, *v*:*v*) resulted in three fractions. The second orange fraction contained the product. The solvent was evaporated giving **8** as a dark orange powder (50 mg, 0.08 mmol, 47%). Crystals of **8** suitable for single crystal X-ray crystallography were obtained by slow evaporation of a DCM solution of compound **8**.

### 2.4. Reagents and Cells

Reagents and cells have been obtained from the following manufacturers: Sigma (St. Louis, MO, USA): dimethyl sulfoxide (DMSO), crystal violet (CV), 3-methyladenine (3-MA), phosphate-buffered saline (PBS), propidium iodide (PI), carboxyfluoresceindiacetate succinimidyl ester (CFSE), fluorescent mounting medium, acridine orange (AO); SERVA Electrophoresis GmbH (Heidelberg, Germany): paraformaldehyde (PFA); AppliChem (Darmstadt, Germany): 3-(4,5-dimethylthiazol-2-yl)-2,5-diphenyltetrazolium bromide (MTT) and bovine serum albumin (BSA); Capricorn Scientific GmbH (Ebsdorfergrund, Germany): culture medium RPMI-1640 and fetal bovine serum (FBS); Biological Industries (Cromwell, CT, USA): penicillin streptomycin solution; BD (Pharmingen, San Diego, CA, USA): Annexin V-FITC (AnnV); R&D Systems (Minneapolis, MN, USA): ApoStat; Thermo Fisher Scientific (Waltham, MA, USA): HEPES (4-(2-hydroxyethyl)-1-piperazineethanesulfonic acid)-buffered RPMI (Roswell Park Memorial Institute)-1640 medium, chloroquine, dihydrorhodamine 123 (DHR 123); American Type Culture Collection (ATCC, Manassas, VA, USA): cell lines (human malignant glioma U251; human breast adenocarcinoma MCF-7, MDA-MB-361, MDA-MB-231).

All cell lines were routinely cultivated in HEPES-buffered RPMI-1640 medium previously supplemented with 10% heat-inactivated FBS, penicillin (100 units mL^−1^) and streptomycin (100 μg mL^−1^) and were grown at 37 °C in a humidified atmosphere with 5% CO_2_. For viability determination, cells were seeded at the following densities in 96-well plates: MCF-7 (1 × 10^4^ cells/well), MDA-MB-231 (8 × 10^3^ cells/well), MDA-MB-361 (7 × 10^3^ cells/well), and U251 (3 × 10^3^ cells/well). For flow cytometric analyses, MCF-7 cells were seeded in 6-well plates in density 2.5 × 10^5^ cells/well.

For the cell treatment, stock solutions (20 mM) of the reference compound **3**, ligand **4** and complexes **5**–**8** were prepared in DMSO and stored at −20 °C or directly used. The stock solution of chloroquine was prepared according to the supplier’s data sheet. The stock solution was diluted with the medium to prepare the final working concentrations. The highest final concentration of DMSO was 0.5% (*v*/*v*).

### 2.5. Bioanalytical Measurements

#### 2.5.1. Peritoneal Exudates Cells (PEC)

C57BL/6 mice were used for isolation of peritoneal exudates cells. The origin of animals was the animal facility at the Institute for Biological Research “Siniša Stanković”, National Institute of the Republic of Serbia, University of Belgrade (Belgrade, Serbia). The handling of the animals was in accordance with local guidelines and approved by the local Institutional Animal Care and Use Committee (IACUC). After isolation the cells were cultivated in HEPES-buffered RPMI-1640 medium supplemented with 5% (*v*/*v*) heat-inactivated FBS and antibiotics under standard growth conditions. Cells were seeded at density 1.5 × 10^5^ cells/well in 96-well plates and left for two hours to adhere. Prior to treatment, non-adherent cells were removed. After 72 h treatment, cell viability was determined using CV and MTT assays.

#### 2.5.2. Determination of Cell Viability (MTT and CV Assays)

All cell lines were seeded in suitable densities overnight and treated with the tamoxifen derivatives **3**–**8** for 72 h. After incubation, the supernatant was discarded, and the cells were washed with PBS. MTT solution (0.5 mg mL^−1^) was added and incubated at 37 °C until purple formazan crystals were formed. The dye solution was discarded and DMSO was added to dissolve the formed formazan.

For CV assay, after the treatment the cells were fixed for 10 min with 4% (*v*/*v*) of paraformaldehyde (PFA) and stained with 1% (*v*/*v*) CV solution for 15 min at room temperature (rt). Afterward, cells were washed with tap water and dried. Prior to absorbance measurement, the dye was dissolved in acetic acid.

For both assays absorbance was measured at λ_max_ = 540 nm, with the reference/background wavelength 670 nm. Results were expressed as a percentage of the control value (100%).

#### 2.5.3. Annexin V (AnnV)/Propidium Iodide (PI), ApoStat and Acridine Orange (AO) Staining

For detection of apoptosis, cells were treated with IC_50_ value concentrations of compounds **4**–**8** for 60 h. Afterwards, cells were washed with PBS and stained with AnnV and PI (15 μg mL^−1^) during 15 min at rt protected from light according to the manufacturer’s instructions. At the end, cells were resuspended in AnnV-binding buffer and analysed using flow cytometry. In order to investigate whether apoptosis was mediated by caspase activation, cells were incubated with pan-caspase inhibitor ApoStat. After 30 min incubation at 37 °C, cells were washed with PBS and analysed. To detect the presence of autophagy cells were stained with 1 µg mL^−1^ of AO solution for 15 min at 37 °C. Finally, cells were washed with PBS, resuspended, and analysed by flow cytometry.

#### 2.5.4. Carboxyfluorescein Succinimidyl Ester (CFSE) Staining

The impact of compounds **4**–**8** on cell proliferation was analysed using CFSE staining. Prior to seeding, cells were stained with CFSE to a final concentration of 1 μM for 10 min at 37 °C followed by washing, seeding, and treatment with IC_50_ doses for 60 h. Finally, cells were trypsinised, washed, resuspended in PBS, and analysed by flow cytometry.

#### 2.5.5. Measurement of ROS/RNS Generation

For detection of production of reactive oxygen and nitrogen species (ROS/RNS), cells were pre-stained with 1 µM DHR for 20 min at 37 °C, followed by treatment with compounds **4**–**8** for 60 h. Afterwards, cells were washed, trypsinised, and analysed using flow cytometry.

### 2.6. Statistical Analysis

The data presented represent the means ± SD of at least three independent experiments. Student’s t-test was used to evaluate the significance between groups, and two-sided p values of less than 0.05 were considered statistically significant.

## 3. Results and Discussion

### 3.1. Synthesis and Characterisation

**Scheme 1:**
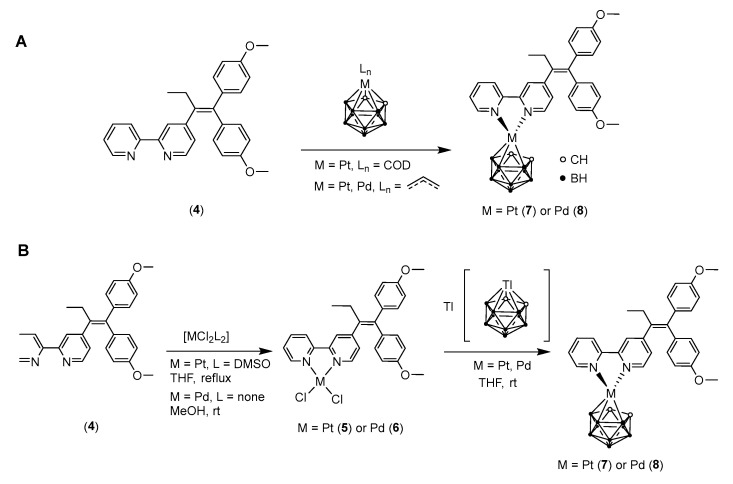
Incorporation of the *nido*-carborate dianion in 2,2′-bipyridine complexes: (**A**) initial formation of metallacarboranes (not shown) followed by reaction with ligand **4**; (**B**) initial formation of metal(II) dichloride complexes **5** and **6** followed by reaction with Tl[*closo*-TlC_2_B_9_H_11_] for chloride replacement.

The ligand 4-[1,1-bis(4-methoxyphenyl)but-1-en-2-yl]-2,2′-bipyridine (**4**) was prepared in four steps as previously reported [22]. Reaction with *cis*-[PtCl_2_(DMSO)_2_] in THF or PdCl_2_ in methanol gives the platinum(II) and palladium(II) dichloride complexes **5** and **6** in good yields (80% and 70%, respectively) (Scheme 1B).

Platina- and palladacarborane complexes with ligand **4** can be prepared either by initial formation of the respective metallacarborane featuring labile ligands followed by complexation with **4**, or by substituting the chloride ligands in **5** and **6** with *nido*-carborate(-2) (Scheme 1). For the first approach, [3-(1′,2′:5′,6′-*η^4^*-COD)-*closo*-3,1,2-PtC_2_B_9_H_11_] (COD = 1,5-cyclooctadiene) [58] and [NEt_4_][3-(*η*^3^-C_3_H_5_)-*closo*-3,1,2-MC_2_B_9_H_11_] (M = Pt, Pd) [59,60] were prepared. However, this approach turned out to be problematic. In the first case, the COD ligand could not be replaced by **4** using various conditions, and starting with [NEt_4_][3-(*η*^3^-C_3_H_5_)-*closo*-3,1,2-MC_2_B_9_H_11_] (M = Pt, Pd) the yield of **7** and **8** was very low (Scheme 1A; for details see Supplementary Materials, Alternative synthetic strategies). On the other hand, complexes **7** and **8** could be prepared in good yields by reacting **5** or **6** with thallium(I) dicarbollide Tl[*closo*-TlC_2_B_9_H_11_] in THF at room temperature (Scheme 1B).

The resulting complexes **7** and **8** were purified using flash chromatography on silica gel as stationary phase and an *n*-hexane/DCM mixture (1:5 for **7** and 1:3 for **8**) as liquid phase. The complexes were characterised by ^1^H, ^11^B, ^11^B{^1^H}, ^13^C{^1^H} NMR, and Fourier-transform infrared spectroscopy (FT-IR), as well as high-resolution electrospray mass spectrometry (HR ESI-MS). The purity of the compound was confirmed by elemental analysis (see Supplementary Materials, Characterisation).

The colours of complexes **5**–**8**, assessed by UV–Vis spectroscopy, result mainly from charge-transfer (CT) transitions between the ligands and the metals in both directions or between two different ligands (metal-to-ligand, MLCT, ligand-to-metal, LMCT, or ligand-to-ligand, LLCT). The coordination of the colourless ligand **4** with the colourless precursor complex [PtCl_2_(DMSO)_2_] resulted in the formation of **5** as an orange solid; the reaction of PdCl_2_ with compound **4** (L) gave **6** as a yellow solid. The dark red or dark orange colour of [3-(L-κ^2^*N*,*N*′)-3,1,2-PtC_2_B_9_H_11_] and [3-(L-κ^2^*N*,*N*′)-3,1,2-PdC_2_B_9_H_11_], respectively, are due to additional LLCTs between the *nido*-carborate dianion and **4** (Figures S35–S38, Supplementary Materials, UV-vis spectroscopy and transitions).

The ^1^H NMR spectra of complexes **5**–**8** support the coordination of the ligand (Figures S27 and S28, Supplementary Materials). Additionally, the signal for the CH_cluster_ protons in the platina- and palladacarborane complexes appears as a broad singlet at 3.99 and 3.85 ppm, respectively. This is indicative for transition metal carborate complexes [22,51]. In the FT-IR spectra of **7** and **8**, the ν(B–H) stretching frequency is observed at 2529 cm^−1^ and 2509 cm^−1^, respectively, verifying the presence of the carborane cluster. Finally, single crystals suitable for X-ray crystallography could be obtained demonstrating the coordination only to the boron atoms of the five-membered ring of the *nido*-carborate cluster (Figure S26, Supplementary Materials).

The stability of complexes **5**–**8** was assessed via NMR spectroscopy in water-containing DMSO solution in air at rt and kept below 4 °C between the measurements. According to the ^1^H NMR spectra, the dichlorido complexes **5** and **6** appear to be stable over 30 days in solution (Figures S29 and S30, Supplementary Materials). However, an exchange of the chloride ligands in water-containing solutions and under biological conditions, as it was shown for other platinum-containing drugs [61], cannot be excluded. On the other hand, palladacarborane **8** is slowly dissociating releasing the *nido*-cluster after 3 days (Figures S33 and S34, Supplementary Materials), as confirmed by ^11^B{^1^H} NMR spectroscopy. For complex **7**, a broad signal at ca. 20 ppm appeared in the ^11^B{^1^H} NMR spectrum after seven days, which could not be assigned to any typical decomposition product (Figure S32, Supplementary Materials). We assume that this signal is related to formation of self-assembled species in solution as was previously shown also for molybda- [22] and ruthenacarboranes [51]. The ^1^H NMR spectra, however, remained unaffected over time (Figure S31, Supplementary Materials).

### 3.2. Bonding Interactions

**Figure 2:**
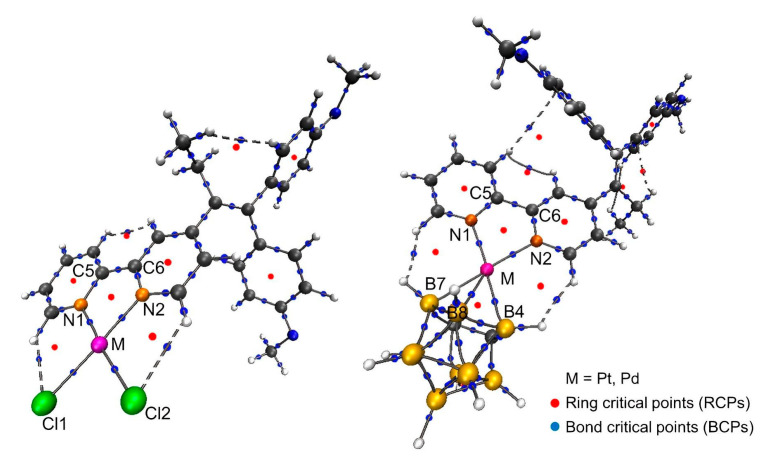
QTAIM analysis: molecular graphs of metal(II) dichlorido complexes (**5** and **6**, **left**) and the metallacarboranes (**7** and **8**, **right**). The bond paths of the non-covalent interactions are shown as dashed lines. The bond critical points (BCPs) and the ring critical points (RCPs) are indicated as blue or red dots.

The stability of complexes **5**–**8** was also investigated with the Quantum Theory of Atoms in Molecules (QTAIM) approach (Supplementary Materials, QTAIM: bonding interactions) [62]. In this theory, the molecule can be described in terms of so-called critical points (CPs) as extrema in the charge density plot (ρ(r)) [62,63]. There are four stable types of CPs; however, here we focused on two types only, namely the bond critical points (BCPs) [63] and the ring critical points (RCPs) [64]. BCPs characterise the interactions between two atoms by indicating a maximum of charge density between them, while RCPs appear inside a ring being formed by the geometric arrangement of several atoms (e.g., phenyl rings). The gradient lines connecting BCPs and nuclei are the bond paths, the set of which form the molecular graph (Figure 2).

The bond lengths and several topological parameters in CPs such as electron density (ED, ρ_cp_), Laplacian of ED (∇^2^ρ_cp_) [63,65], potential energy density (V_cp_) [66], and total electron energy density (H_cp_) [62], which is the sum of the positive kinetic (G_cp_) and negative potential energy densities [63,67], were analysed. These parameters allow the estimation of the stability of an interaction between two atoms or between a group of atoms in the curtain region of a molecule. In particular, low H_cp_ values in BCP or RCP indicate stronger bonding [63,65]. Additionally, the ratio of V_cp_ and G_cp_ is an indicator for the stability of an interaction; thus, the higher the values the more stable the interaction (Table 1).

The RCPs of the ring M−N1−C5−C6−N2 (M = Pt, Pd) indicate that in the complexes **5** and **6** the interactions between the metal and the ligand **4** are stronger than the M−Cl interactions. In other terms, the possibility of dissociation of the M−Cl bond and release of Cl^−^ is higher. As reported in the literature [68,69], this is the main activation route of platinum(II) and palladium(II) dichlorido complexes in aqueous systems. Incorporation of the *nido*-cluster resulting in complexes **7** and **8** changes the electronic structure situation. The decrease in the ED in the M−N1−C5−C6−N2 ring (RCP from 0.322 in **5** to 0.121 in **7** and from 0.326 in **6** to 0.120 in **8**) clearly demonstrates the electron-withdrawing effect of the dicarbollide anion. However, comparing ∑ρ_cp_ and ∑H_cp_ for RCPs and BCPs in **7**, consisting of B−Pt BCPs and Pt−B4−B7 RCP (0.336 and −0.137, respectively; ∑(Pt−B)), as well as of Pt−N1−C5−C6−N2 RCP and N−Pt BCPs (0.347 and −0.147, respectively; ∑(Pt−N)), a stronger interaction between ligand **4** and Pt^2+^ than between the dicarbollide anion and Pt^2+^ can be concluded. A similar trend was observed for Pd−[C_2_B_9_H_11_] in **8**, where ∑ρ_cp_ and ∑H_cp_ are 0.229 and −0.103, respectively, for the ∑(Pd−B) contacts, and 0.310 and −0.112 for ∑(Pd−N) interactions. Laplacian of ED is an additional indicator of the interaction stability pointing out the accumulation ($\nabla^{2}$ρ_cp_ < 0 for more stable interactions) or depletion ($\nabla^{2}$ρ_cp_ > 0 for less stable interactions) of electron charge. Thus, negative $\nabla^{2}$ρ_cp_ values indicate the accumulation of electron density between ligand **4** and the metal cations for M−N1−C5−C6−N2 RCPs in **7** and **8**, while $\nabla^{2}$ρ_cp_ values are positive for M−(B4, B7) (M–cage) RCPs meaning a depletion of ED between metal cations and dicarbollide. Importantly, the comparison of compounds **7** and **8** revealed higher covalency of the B−Pt bonds (**7**, with a greater ratio of |V_cp_|/G_cp_ equal to 2.1 for B4−Pt and B7−Pt) compared to the B−Pd bonds (**8**, equal to 1.8 for B4−Pd and B7−Pd) pointing towards a more stable Pt−[C_2_B_9_H_11_] unit. In conclusion, the bonding interactions between [C_2_B_9_H_11_]^2−^ and Pt^2+^ are stronger than between [C_2_B_9_H_11_]^2−^ and Pd^2+^; thus, palladacarborane **8** may dissociate with higher probability compared to **7**, releasing the *nido*-carborane cluster in solution.

In silico modelling of the interaction between compounds **3**−**9** and the target protein ERα was conducted by docking their DFT geometry-optimised X-ray molecular structures into the C-terminal ligand-binding domain (LBD) (Supplementary Materials, Docking). The comparison of the binding energy of the reference molecule **3** (−10.73 kcal mol^−1^), the ligand **4** (−8.44 kcal mol^−1^), and the ligand **9** (−8.76 kcal mol^−1^) demonstrated that the presence of 2,2′-bpy instead of a phenyl ring decreased the binding energy of both ligands (decreased the affinity), while the presence (**4**) or absence (**9**) of the methyl groups in the bisphenol part of the ligand hardly changed the binding energies. Interestingly, the incorporation of PtCl_2_ (**5**) or PdCl_2_ (**6**) slightly improved the binding strength compared to the ligand **4** alone (−9.08 kcal mol^−1^ for **5** and −8.99 kcal mol^−1^ for **6**). Incorporation of [PtC_2_B_9_H_11_] in **7** decreased the binding energy (−8.12 kcal mol^−1^), while **8** showed the best interaction of all investigated compounds with a binding energy of −9.71 kcal mol^−1^. In the in silico study, we have considered the affinity of the synthesised compounds as tamoxifen-inspired structures towards one target, namely ERα, and have compared it to tamoxifen and its metabolites. It can, however, not be excluded that our compounds may exhibit higher affinity to other protein targets in an off-target mechanism.

### 3.3. In Vitro Cytotoxicity Studies

To evaluate the cytotoxic potential of the ligand **4** and the metal complexes **5**−**8** in comparison to the literature-known compound **3**, in vitro cell toxicity studies with three ERα-expressing (U251, MCF-7, MDA-MB-361) and one triple-negative (MDA-MB-231) cancer cell lines were performed. Stock solutions of all compounds were prepared in DMSO and diluted with the cell medium before the treatment of the cell lines. After 72 h of incubation with compounds **3**−**8**, cell viability was determined by measuring the total mitochondrial respiration and number of adherent cells in cultures, using MTT (3-(4,5-dimethylthiazol-2-yl)-2,5-diphenyltetrazolium bromide) and CV (crystal violet) assays, respectively (Table 2).

Treatment of all cell lines with compound **3** resulted in a decrease in viability with IC_50_ values varying between 20 and 30 µM. Compound **3** was used also in one of our previous studies, but with a bovine serum albumin (BSA) formulation strategy, which is the reason for the discrepancy of the IC_50_ values observed here (ca. ± 10 µM for breast cancer cell lines and >80 µM for U251) [22]. As compounds **9**–**11** were also pre-incubated with BSA [22], their toxicity cannot be directly compared with the complexes **5**−**8** studied here.

Ligand **4** and metal complexes **5** and **6** showed significantly higher potency in all tested cell lines with activities even 10x higher than the reference compound **3**. The incorporation of the dicarbollide ligand changed the cytotoxicity of compounds **7** and **8** compared to **5** and **6**, being strongly dependent on cell line and nature of the metal. Previously, it was shown that the cytotoxicities of 2,2′-bipyridine-modified tamoxifen derivates were generally decreased when [MoC_2_B_9_H_11_] was incorporated [22]. A similar trend for the cytotoxicity was observed for **7** (towards all tested cell lines) and **8** (towards MCF-7 and MDA-MB-361), demonstrating a decreased activity when platina- and palladacarboranes were incorporated in the ligand structure **4**. Interestingly, the presence of the [PdC_2_B_9_H_11_] moiety (**8**) instead of PdCl_2_ (**6**) made complex **8** highly selective against triple-negative breast adenocarcinoma (MDA-MB-231) and moderately toxic against ERα+ glioma (U251) cell lines. This suggests the possibility to fine-tune the cytotoxic behaviour of ligands against certain types of cancers by varying the metallacarborane unit.

As compounds **4**–**6** and **8** are active against both ERα+ and ERα− cell lines, the antiproliferative efficacy could be hormone receptor-independent, suggesting the existence of (additional) off-targets.

**Figure 3:**
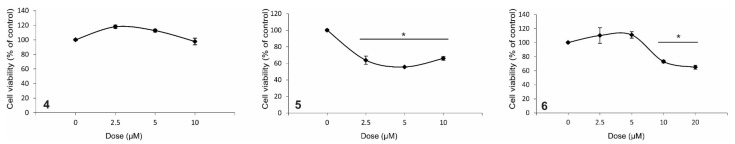
The effect of compounds **4**−**6** on the viability of peritoneal exudate cells. Peritoneal exudate cells were treated with **4**−**6** and subjected to CV viability assay after 72 h. The data are expressed as percentage of viability of untreated control (set as 100%) from one representative out of three independent experiments and presented as mean ± SD of triplicate cultures (* *p* < 0.05 in comparison to untreated cells).

In order to investigate the selectivity for cancer cells, peritoneal exudate cells, as a model of primary cells, were treated with ligand **4** and the most cytotoxic complexes of our series, namely **5** and **6**. Ligand **4** has no statistically significant effect on the viability of these cells in the dose range between 0 and 10 µM, with the maximal dose being two to five time higher than the average IC_50_ doses determined for different tumour cell lines (Figure 3).

On the other hand, treatment with compounds **5** or **6** slightly diminished primary cell viability in the indicated dose range, but never reaching cell viability of 50% (thus, no IC_50_ values could be determined). Therefore, the selectivity index cannot be calculated even though the selectivity towards malignant cells is obvious.

### 3.4. Flow Cytometry

**Figure 4:**
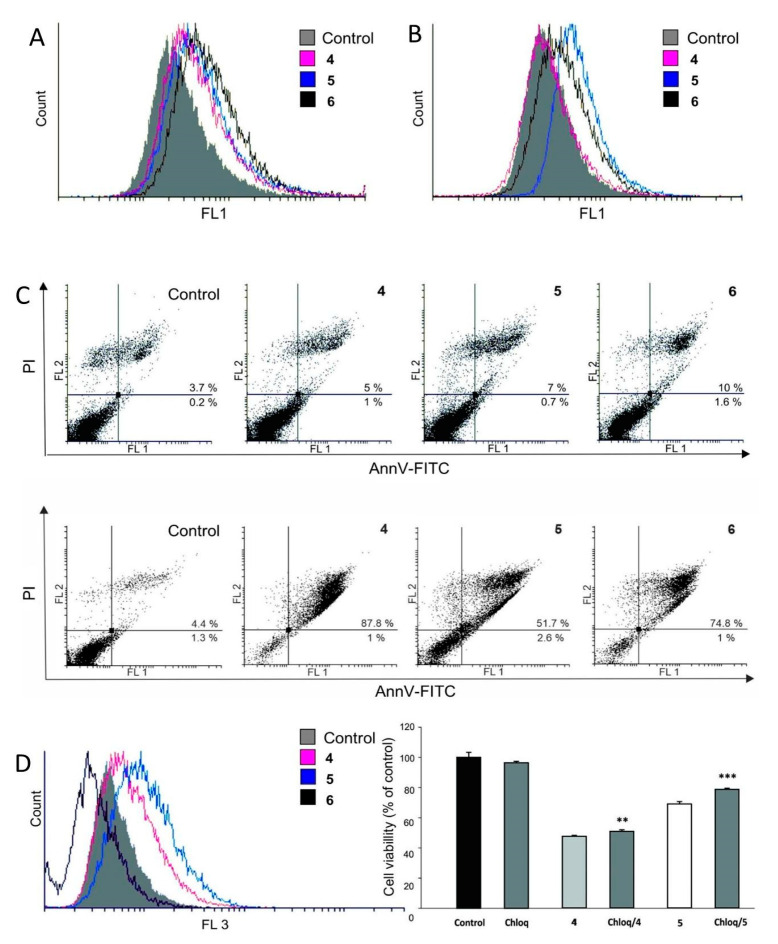
Tamoxifen derivatives attenuated MCF-7 cell division and induced programmed cell death type 1 and 2. MCF-7 cells were exposed to IC_50_ doses of compounds **4**, **5** and **6** for 60 or 72 h and analysed by flow cytometry: (**A**) cell proliferation rate, fluorescence intensity from FL1 channel (exc. 488 nm/em. 536 nm); (**B**) ApoStat staining for caspase-dependent apoptosis, fluorescence intensity from FL1 channel; (**C**) apoptosis detection (AnnV-FITC/PI staining) after 60 h (upper panels) or 72 h (lower panels), fluorescence intensity from FL2 channel (y axis, exc. 488 nm/em. 590 nm) vs. FL1 channel (x axis); (**D**) autophagy detection after 60 h and determination of the role of autophagy by specific inhibitor chloroquine (Chloq) (20 µM), fluorescence intensity from FL3 channel (exc. 488 nm/em. 675 nm). The data are presented as mean ± SD of triplicate cultures (** *p* < 0.01; *** *p* < 0.001 refers to compound-treated cultures. Representative dot plots and histograms are shown from at least three independent experiments).

In order to identify potential modes of action of the most potent compounds, MCF-7 cells were exposed to IC_50_ doses of **4**–**6** for 60 h or 72 h. After the end of the cultivation period, flow cytometric assessments were conducted for cell proliferation, caspase activity, apoptotic cell death, and autophagy using the respective indicative dyes. Ligand **4** and complexes **5** and **6** significantly affect the cell division rate and thus exert a cytostatic effect (Figure 4A).

**Figure 5:**
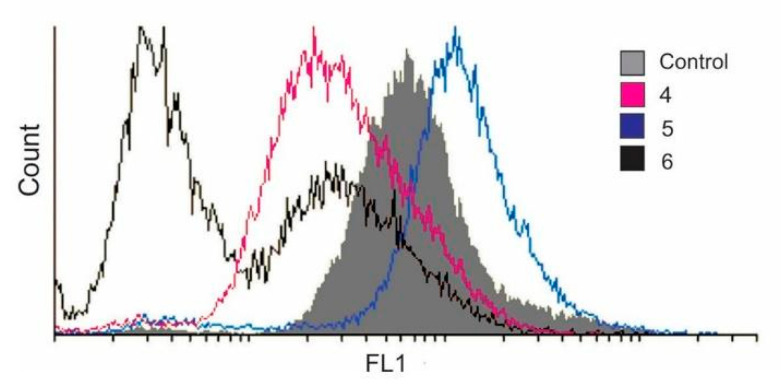
Compounds **4**−**6** differently affect the production of reactive oxygen and nitrogen species (ROS/RNS) in MCF-7 cells. MCF-7 cells were pre-stained with dihydrorhodamine 123 (DHR 123) and treated with **4**, **5** or **6** (IC_50_ dose) for 60 h. Intracellular accumulation of ROS/RNS were measured by flow cytometry. Representative histograms from three independent experiments are shown.

Additionally, activation of caspases was observed after the treatment with complexes **5** and **6** but not for **4** (Figure 4B). To investigate the potential of the experimental compounds to trigger apoptotic cell death, AnnV/PI double staining was performed. AnnV strongly interacts with phosphatidylserine exposed on the outer plasma membrane in early apoptotic phase. Possible changes of the cellular membrane integrity lead to internalisation of PI in the late phase of apoptosis manifested by the appearance of double stained cells (Figure 4C) [70]. The obtained results showed a dramatic increase in late apoptotic cells in the time range between 60 h and 72 h after treatment (5% (**4**), 7% (**5**), 10% (**6**) vs. 88% (**4**), 52% (**5**), 75% (**6**), Figure 4C). In concordance with this, staining of cells with the DNA-specific dye, PI, showed shrunken nuclei with condensed chromatin, irregular shape, and decreased nuclear volume as morphologic signs of apoptosis, confirming the presence of typical apoptotic cell death in response to the treatments (Figure S41, Supplementary Materials). Taken together, the combination of platinum or palladium dichloride with 4-[1,1-bis(4-methoxyphenyl)but-1-en-2-yl]-2,2′-bipyridine converted the apoptotic process from caspase-independent to caspase-dependent. Treatment of cells with compounds **4** and **5**, but not **6**, strongly potentiated the presence of acidic vesicles in the cytoplasm recognised as autophagosomes (Figure 4D, left panel). This process is often the regular cell response to stress induced by external or internal factors, as well as applied therapy [71]. Its role can vary from cytoprotective to destructive depending on the extend of intracellular structure damage. In order to investigate the contribution of autophagy in drug action, the cells were treated with **4** or **5** in combination with chloroquine (20 µM). Chloroquine is an autophagy inhibitor preventing the fusion of autophagosomes and used quite often in combination with platinum drugs [72,73]. Here, this co-treatment led to statistically significant restoration of cell viability, confirming the contribution of autophagic cell death to the repertory of compounds **4** and **5** antitumour activities (Figure 4D). The ability of ligand **4** and complexes **5** and **6** to generate reactive oxygen and nitrogen species (ROS/RNS) was investigated using dihydrorhodamine 123 (DHR 123) staining (Figure 5). While the platinum(II) complex **5** potentiated oxidative stress as other platinum(II) and platinum(IV)-based compounds [74], ligand **4** and the palladium(II) complex **6** downregulated ROS/RNS production possibly exerting scavenging potential (Figure 5). Similar behaviour regarding scavenging properties was previously observed for ligand **9** and complex **11** [22].

Taken together, the obtained results showed that the fusion of the lead structure of tamoxifen with a 2,2′-bpy moiety leads to an enhanced cytotoxic potential against ERα+/− tumour cell lines compared to compound **3** (see CV and MTT assays in Table 2). The mechanisms of action include combined cytostatic and cytotoxic effects, including both inhibition of cell proliferation and induction of programmed cell death of types 1 (apoptosis) and 2 (autophagic cell death). The choice of the metal is essential for the redox response of the cells to the treatment and can vary from scavenging potential observed for ligand **4** and complex **6**, to oxidative burst observed for compound **5**.

## 4. Conclusions

In order to overcome resistance in tamoxifen therapy, derivatives of the tamoxifen lead structure bearing a chelating unit (2,2′-bpy), well-known in coordination chemistry, were developed as ligands for a variety of known bioactive metal-containing moieties. This strategy allows for a modular combination of tamoxifen derivatives and metals to fine-tune modes of action for scenarios in which new resistances have been developed, as is it also known for metal-containing drugs [75]. Here, we have extended our previous studies to known bioactive transition metal moieties, namely PtCl_2_ (**5**), PdCl_2_ (**6**), 3,1,2-PtC_2_B_9_H_11_ (**7**), and 3,1,2-PdC_2_B_9_H_11_ (**8**) complexes with the tamoxifen derivative **4** and present their syntheses and spectroscopic and theoretical/computational characterisation followed by an assessment of their cytotoxic activity in in vitro cell cultures.

The formal exchange of two chloride ligands in **5** and **6** by an electron-withdrawing dianionic *nido*-carborane showed the influence of the latter on the electronic properties of compounds **7** and **8** as well as the activity and selectivity of the corresponding tamoxifen-based platinum(II) and palladium(II) complexes towards selected cancer cell lines.

Even though our docking studies predicted binding strengths for ERα in the order **3** > **8** > **5** ≈ **6** > **4** > **7**, it can be excluded by our biological results that this is the main mode of action under the applied conditions. Thus, **8** is highly selective for triple-negative (ERα−) MDA-MB-231 cells, while **7** is completely inactive against this cell line. The observed cytotoxicity of compounds **4**−**6** and **8** against this triple-negative cell line suggests other off-target mechanisms rather than only ERα inhibition, for which these compounds were originally designed. Similarly, for ferrocifen oestrogen activity is observed only for nanomolar concentrations, while other modes of action, such as senescence (ca. 10^−7^ M), apoptosis (ca. 10^−6^ to 10^−5^ M), or Fenton chemistry (ca. 10^−5^ to 10^−4^ M) [76,77] become more important at higher concentrations [78].

The strong cytotoxic activity of ligand **4** is comparable to its metal complexes **5** and **6**, suggesting potential metal scavenging properties, which would allow removal of metals essential for functioning of certain biomolecules. For example, 2,2′-bpy appears to be a specific chelator of iron(II), being able to inhibit dopamine β-hydroxylase activity in rats and mice [79] and [^3^H]-spiroperidol to bind to dopamine receptor 2 [80]. Additionally, this moiety can moderately induce DNA cleavage, as was shown for leukaemia and sarcoma cell lines [81].

Compounds **5** and **6** exhibit similar behaviour and stability in solution according to their ^1^H NMR spectra, while metallacarboranes **7** and **8** behave differently based on ^11^B{^1^H} NMR spectroscopic data. The literature studies [68,69] and QTAIM calculations suggest that the N,N-chelating ligand is substantially stronger bound to the respective metal centre than the chloride ligands in **5** and **6**, while in compounds **7** and **8**, the carborane cluster is the stronger ligand. However, the B−Pd bonds exhibit higher ionic character compared to B−Pt and thus indicate the possibility for a more facile dissociation with release of the *nido*-cluster for complex **8.** The potential release of chloride in complexes **5** and **6** should facilitate interaction with DNA or other cellular biomolecules. As observed for other Pt drugs [37,74], compound **5** also potentiates the oxidative stress possibly by acting as a DNA crosslinker and additionally by DNA damaging through ROS generation. This possible ligand exchange and subsequent unselective coordination to biomolecules such as DNA or proteins might be the reason why **5** and **6** outperform **7** and **8** on all tested cancer cell lines and could also cause the observed delayed strong increase in apoptotic cells after treatment with **5** or **6**.

## Figures and Tables

**Table 1:** Comparison of selected bond lengths and topological parameters of the bond critical points’ analyses: electron density (ρ_cp_), Laplacian of electron density (∇^2^ρ_cp_), total electron energy density (H_cp_), and the ratio of the potential to kinetic energy densities |V_cp_|/G_cp_. The single crystal X-ray crystallography data of **5**–**8** were used as input, and the geometries were optimised at PBE0 D3BJ/ZORA-def2-TZVPP level of theory using DFT.

Complex	Bond or Ring	Bond Length, Å	ρ_cp_, a.u.	$\nabla^{2}\rho_{\mathrm{cp}},\;a.u.$	H_cp_, a.u.	|V_cp_|/G_cp_
**5**	Pt−N1−C5−C6−N2	-	0.322	−0.969	−0.356	4.3
	N1−Pt	2.019	0.131	0.465	−0.051	1.3
	Cl1−Pt	2.305	0.108	0.210	−0.045	1.5
**6**	Pd−N1−C5−C6−N2	-	0.326	−0.991	−0.356	4.3
	N1−Pd	2.017	0.113	0.452	−0.032	1.2
	Cl1−Pd	2.276	0.098	0.236	−0.031	1.5
**7**	B4−Pt	2.191	0.099	−0.012	−0.045	2.1
	B8−Pt	2.183	0.095	0.035	−0.043	1.8
	B7−Pt	2.179	0.100	−0.014	−0.046	2.1
	Pt−(B4, B7) (Pt–cage)	-	0.042	0.144	−0.003	1.1
	∑(Pt−B)	-	0.336	-	−0.137	-
	N1-Pt	2.077	0.112	0.398	−0.038	1.3
	N2-Pt	2.082	0.114	0.401	−0.039	1.3
	Pt−N1−C5−C6−N2	-	0.121	−0.121	−0.069	2.8
	∑(Pt−N)	-	0.347	-	−0.147	-
**8**	B4−Pd	2.178	0.089	0.031	−0.037	1.8
	B8−Pd	2.270	0.078	0.109	−0.026	1.5
	B7−Pd	2.190	0.090	0.029	−0.037	1.8
	Pd−(B4, B7) (Pd–cage)	-	0.042	0.156	−0,003	1.0
	∑(Pd−B)	-	0.229	-	−0.103	-
	N1−Pd	2.111	0.096	0.400	−0.022	1.2
	N2−Pd	2.098	0.094	0.395	−0.021	1.2
	Pd−N1−C5−C6−N2	-	0.120	−0.120	−0.069	2.8
	∑(Pd−N)	-	0.310	-	−0.112	-

**Table 2:** IC_50_ values (µM) of compounds **3**−**8** from MTT and CV assays after 72 h incubation shown as mean together with one standard deviation (mean ± SD). Compound **3** served as reference.

Compound	Assay	U251	MCF-7	MDA-MB-361	MDA-MB-231
**3**	MTT	28.1 ± 1.4	14.3 ± 2.9	28.6 ± 0.2	24.5 ± 0.7
	CV	20.1 ± 1.9	19.1 ± 1.3	36.2 ± 2.1	26.2 ± 3.7
**4**	MTT	4.1 ± 0.3	2.5 ± 0.4	4.8 ± 0.0	2.1 ± 0.0
	CV	4.4 ± 0.3	2.8 ± 0.1	5.8 ± 0.2	2.3 ± 0.0
**5**	MTT	2.4 ± 0.4	5.4 ± 0.4	5.5 ± 0.1	2.2 ± 0.1
	CV	3.0 ± 0.3	7.3 ± 0.7	6.2 ± 0.0	2.6 ± 0.1
**6**	MTT	4.4 ± 0.3	2.7 ± 0.6	9.3 ± 0.2	2.0 ± 0.1
	CV	5.9 ± 0.2	4.4 ± 0.4	11.4 ± 1.0	2.3 ± 0.1
**7**	MTT	>100	>100	>100	>100
	CV	>100	>100	>100	>100
**8**	MTT	30.8 ± 1.4	>100	>100	3.7 ± 0.1
	CV	25.3 ± 2.8	>100	>100	5.2 ± 0.1

## Data Availability

The data presented in this study are available in the Supplementary Materials.

## Supplementary Materials

The following supporting information can be downloaded at: https://www.mdpi.com/article/10.3390/pharmaceutics15020682/s1: Characterisation of compounds **4**−**8** (^1^H, ^13^C, ^11^B (**7** and **8**) NMR spectroscopy, IR (**5**−**8**), HR-ESI MS, elemental analysis, X-ray crystallography data for **5**−**8**, stability studies of complexes **5**−**8** by NMR spectroscopy, alternative synthetic strategy for **7** and **8**, computational chemistry (geometry optimisation, UV-vis transitions, QTAIM: bonding interactions, docking studies), bioanalytical measurements (PI staining, cell viability). References [82,83,84,85,86,87,88,89,90,91,92,93,94,95,96,97,98,99,100,101,102,103,104] are cited in the Supplementary Materials.
