# Correction: Kazimir et al. Metallodrugs against Breast Cancer: Combining the Tamoxifen Vector with Platinum(II) and Palladium(II) Complexes. *Pharmaceutics* 2023, *15*, 682

**DOI:** 10.3390/pharmaceutics15061769

**Published:** 2023-06-19

**Authors:** Aleksandr Kazimir, Benedikt Schwarze, Peter Lönnecke, Sanja Jelača, Sanja Mijatović, Danijela Maksimović-Ivanić, Evamarie Hey-Hawkins

**Affiliations:** 1 Institute of Inorganic Chemistry, Faculty of Chemistry and Mineralogy, Leipzig University, 04103 Leipzig, Germany; alex.kazimir95@gmail.com (A.K.); loenneck@rz.uni-leipzig.de (P.L.); 2 Institute for Medical Physics and Biophysics, Medical Faculty, Leipzig University, 04107 Leipzig, Germany; benedikt.schwarze@medizin.uni-leipzig.de; 3 Department of Immunology, Institute for Biological Research “Siniša Stanković”, National Institute of Republic of Serbia, University of Belgrade, 11060 Belgrade, Serbia; sanjajelaca93@gmail.com (S.J.); sanjamama@ibiss.bg.ac.rs (S.M.); nelamax@ibiss.bg.ac.rs (D.M.-I.)

## Error in Scheme 1

In the original publication [1], there was an error in Scheme 1 as published. Scheme 1 was cut off at the left-hand side. The error happened during the final stage of publishing by the journal, and the authors provided the proper version in the corrected proof. The corrected Scheme 1 appears below.

**Scheme 1:**
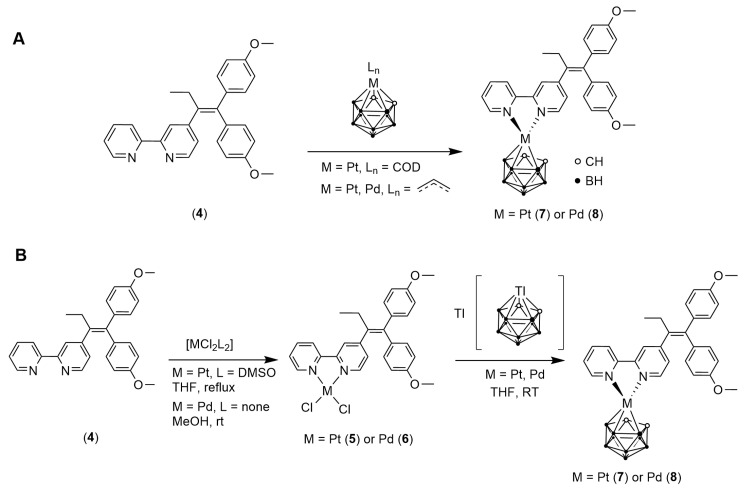
Incorporation of the *nido*-carborate dianion in 2,2′-bipyridine complexes: (**A**) initial formation of metallacarboranes (not shown) followed by reaction with ligand **4**; (**B**) initial formation of metal(II) dichloride complexes **5** and **6** followed by reaction with Tl [*closo*-TlC_2_B_9_H_11_] for chloride replacement.

The authors apologize for any inconvenience caused and state that the scientific conclusions are unaffected. This correction was approved by the Academic Editor. The original publication has also been updated.
