# Supplementary material for: Metallodrugs against Breast Cancer: Combining the Tamoxifen Vector with Platinum(II) and Palladium(II) Complexes
Source: Pharmaceutics. 2023 Feb 17;15(2):682. doi: 10.3390/pharmaceutics15020682 (PMC9959148; doi:10.3390/pharmaceutics15020682)
Supplement: Supplementary file 1 [file pharmaceutics-15-00682-s001.zip › pharmaceutics-2175367-supplementary.pdf]

SUPPORTING INFORMATION:

Metallodrugs against Breast Cancer: Combining the Tamoxifen Vector with Platinum(II) and Palladium(II) Complexes

A. Kazimir, B. Schwarze, P. Lönnecke, S. Jelača, S. Mijatović, D. Maksimović-Ivanić, E. Hey-Hawkins

Table of Contents

1. Characterisation 3

1.1. 4-[1,1-Bis(4-methoxyphenyl)but-1-en-2-yl]-2,2′-bipyridine (L) (**4**) 3

1.2. [$PtCl_2$(L-κ^2*N,N′*)] (**5**) 5

1.3. [$PdCl_2$(L-κ^2*N,N′*)] (**6**) 8

1.4. [3-(L-κ^2*N,N′*)-3,1,2-$PtC_2B_9H_{11}$] (**7**) 10

1.5. [3-(L-κ^2*N,N′*)-3,1,2-$PdC_2B_9H_{11}$] (**8**) 14

2. X-ray crystallography 17

3. NMR study 22

4. Stability 23

4.1. Complex **5** 24

4.2. Complex **6** 24

4.3. Complex **7** 25

4.4. Complex **8** 27

5. Alternative synthetic strategies 29

6. Computational Chemistry 31

6.1. Geometry optimisation 31

6.2. UV-vis spectroscopy and transitions 31

6.3. QTAIM: bonding interactions 33

6.4. Protein structure preparation 36

6.5. Docking 36

7. Bioanalytical measurements 38

7.1. PI staining on chamber slides 38

7.2. Cell viability 39

References 40

1. Characterisation

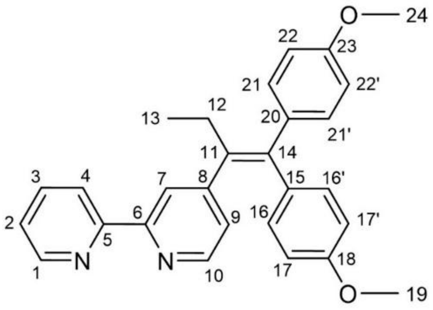

*1.1. 4-[1,1-Bis(4-methoxyphenyl)but-1-en-2-yl]-2,2′-bipyridine (L) (**4**)*

M($C_{28}H_{26}Cl_2N_2O_2$) = 422.53 g mol^{-1}. **^{1}H NMR** (400 MHz, $CDCl_3$) , δ (ppm): 8.67 (m, 1H, 1-C**H**), 8.40 (d, $^3J_{HH}$ = 8.6 Hz, 1H, 10-C**H**), 8.35 (d, $^3J_{HH}$ = 8.0 Hz, 1H, 4-C**H**), 8.30 (s, 1H, 7-C**H**), 7.80 (td, $^3J_{HH}$ = 7.8, $^3J_{HH}$ = 1.8 Hz, 1H, 3-C**H**), 7.29 (ddd, $^3J_{HH}$ = 7.6, $^3J_{HH}$ = 4.7, $^4J_{HH}$ =1.2 Hz, 1H, 2-C**H**), 7.16 (d, $^3J_{HH}$ = 8.5 Hz, 2H, 21,21′-C**H**), 6.93 (dd, $^3J_{HH}$ = 5.0, $^4J_{HH}$ = 1.7 Hz, 1H, 9-C**H**), 6.89 (d, $^3J_{HH}$ = 8.6 Hz, 2H, 22, 22′-C**H**), 6.84 (d, 2H, $^3J_{HH}$ = 8.6 Hz, 2H, 16,16′-C**H**), 6.56 (d, $^3J_{HH}$ = 8.6 Hz, 2H, 17,17′-C**H**), 3.83 (s, 3H, 24-C**H$_3$**), 3.66 (s, 3H, 19-C**H$_3$**), 2.59 (q, $^3J_{HH}$ = 7.3 Hz , 2H, 12-C**H$_2$**), 0.97 (t, $^3J_{HH}$ = 7.4 Hz, 3H, 13-C**H$_3$**). **^{13}C{^{1}H} NMR** (101 MHz, $CDCl_3$), δ (ppm): 158.6 (23-**C**), 158.0 (18-**C**), 156.3 (5-**C**), 155.9 (6-**C**), 152.3 (8-**C**), 149.1 (1-CH), 148.7 (10-**C**H), 140.2 (14-**C**), 138.7 (11-**C**), 136.9 (3-**C**H), 135.5 (20-**C**), 134.8 (15-**C**), 132.0 (16, 16′-CH), 130.5 (21,21′-**C**H), 125.8 (9-**C**H), 123.6 (2-**C**H), 121.3 (7-**C**H), 121.1 (4-**C**H), 113.6 (22, 22′-**C**H), 113.1 (17, 17′-**C**H), 55.3 (24-**C**H$_3$), 55.0 (19-**C**H$_3$), 28.4 (12-**C**H$_3$), 13.6 (13-**C**H$_2$). **Elemental analysis:** $C_{28}H_{26}N_2O_2{\cdot}H_2O$ calc. (%) C 76.34 H 6.41 N 6.36, found (%) C 76.75 H 6.38 N 6.17.

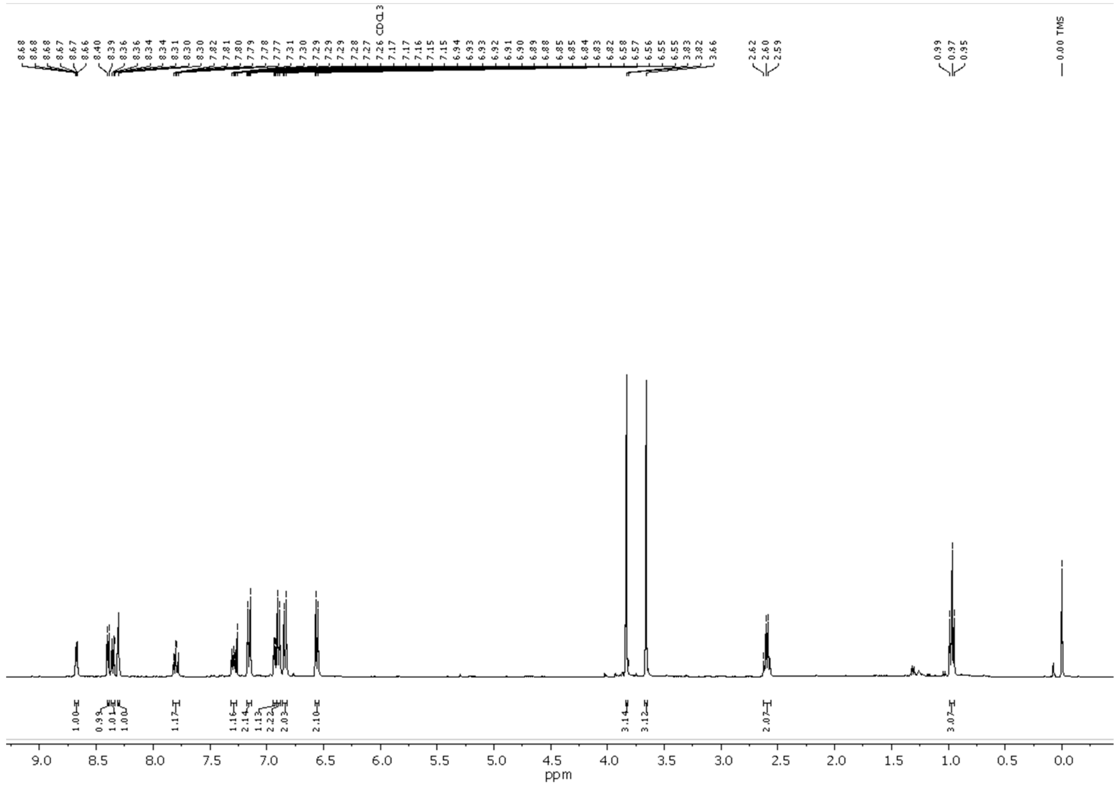

Figure S1. ^{1}H NMR spectrum of compound **4** in $CDCl_3$.

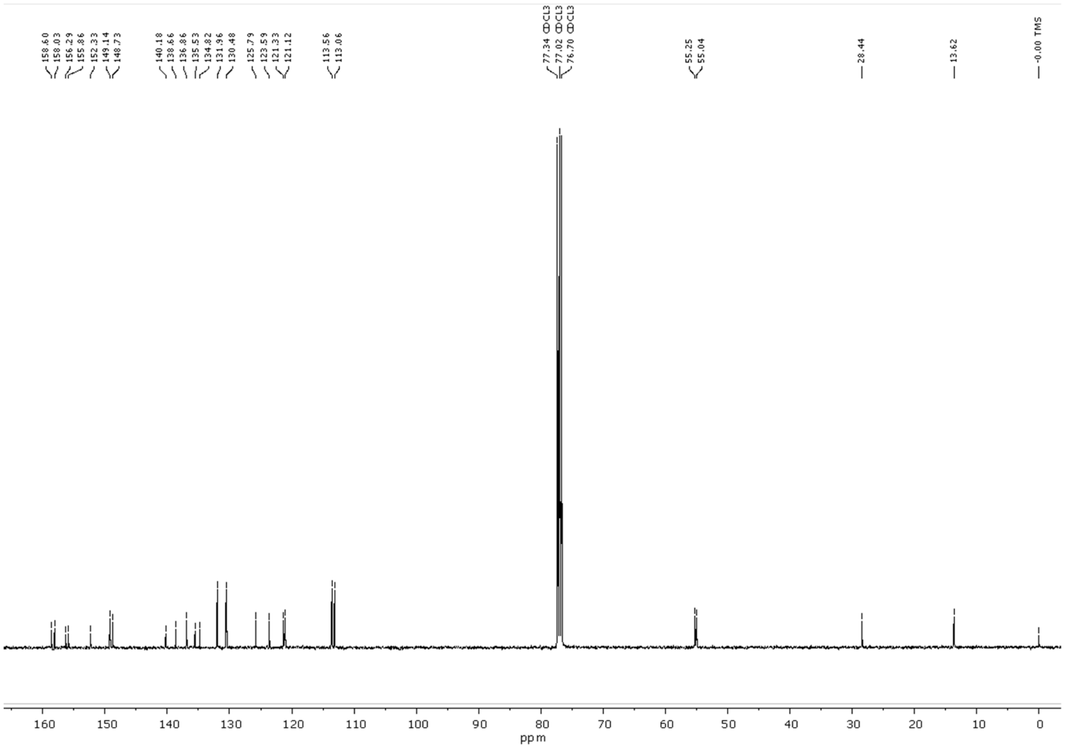

Figure S2. $^{13}C\{^{1}H\}$ NMR spectrum of compound **4** in $CDCl_3$.

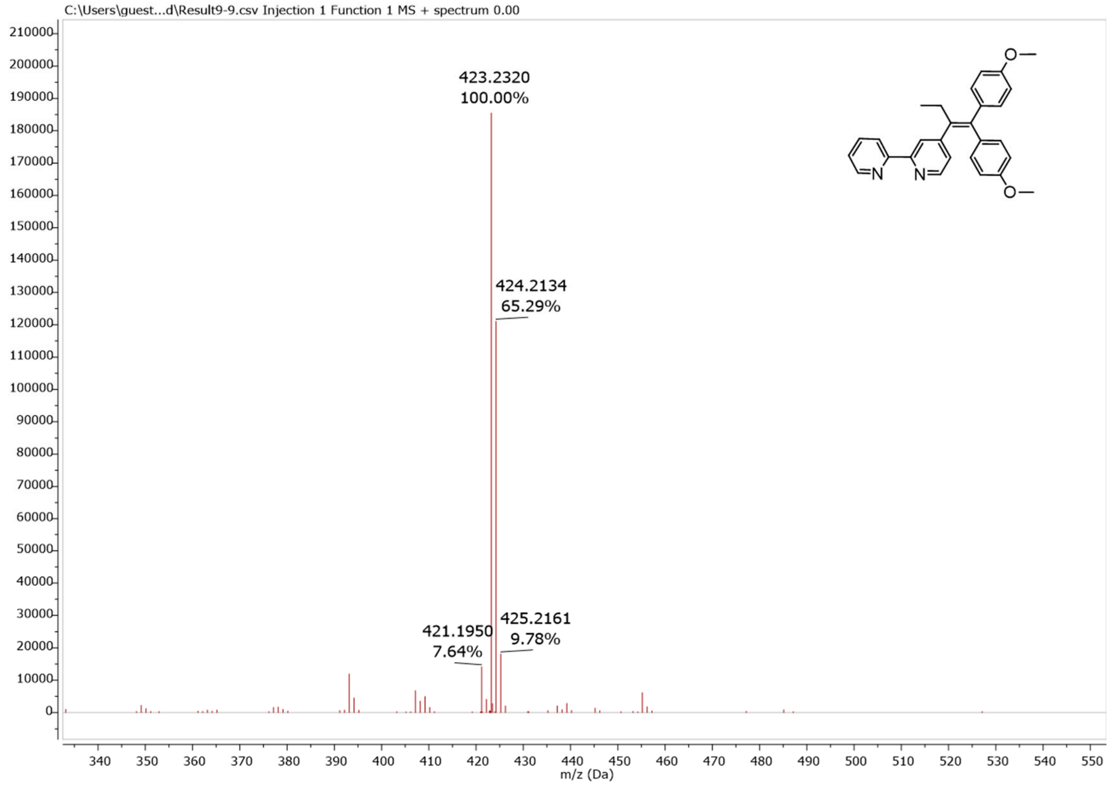

Figure S3. HR-ESI-MS (positive mode, acetonitrile) of **4**: m/z $[M+H]^+$ = 423.2072 (calc.), 423.2320 (found).

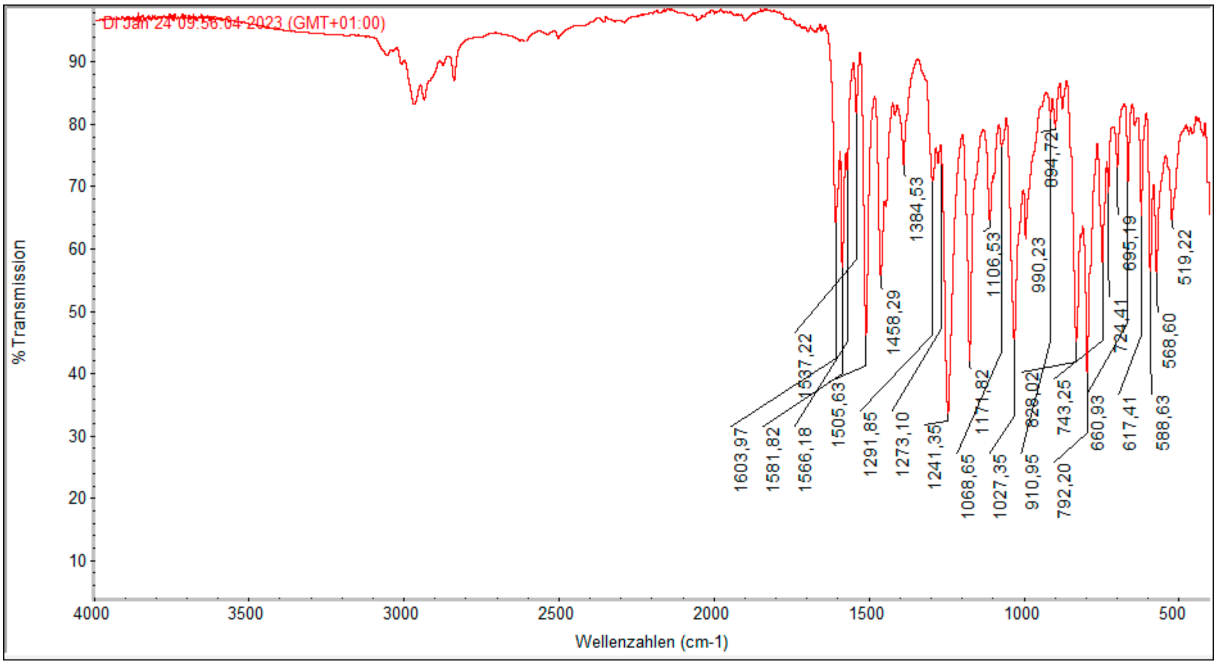

Figure S4. IR spectrum of **4**: 3050–2084 (w, C_{alk}–H), 1604 (m, ν(C=C)), 1582 (m, ν(C=C)), 1537 (w, ν(C=C)), 1506 (s, C_{arom}–H in-plane bending), 1458 (w, C_{arom}-H in-plane binding), 1384 (w, C_{arom}–H in-plane bending), 1292 (w), 1273 (w, C_{arom}–O), 1241 (s, C_{arom}-O), 1172 (s, C_{arom}–O), 1107 (m), 1069 (w), 1027 (s, C_{alk}–O), 828 (m, out-of-plane bending), 792 (m, out-of-plane bending) 743 (s, out-of-plane bending), 724 (w), 661 (w), 627(w), 589 (w), 567 (w), 519 (w).

*1.2. [$PtCl_2$(L-κ²N,N′)] (**5**)*

M($C_{28}H_{26}Cl_2N_2O_2Pt$) = 687.10 g mol^{-1}, **^{1}H NMR** (400 MHz, $CDCl_3$), δ (ppm): 9.68 (d, $^3J_{HH}$ = 5.9 Hz, 1H, 1-C**H**), 9.44 (d, ^3J = 6.1 Hz, 1H, 10-C**H**), 8.07 (td, $^3J_{HH}$ = 7.8, $^3J_{HH}$ = 1.5 Hz, 1H, 3-C**H**), 7.66 (d, $^3J_{HH}$ = 8.0 Hz, 1H, 4-C**H**), 7.63 (d, $^3J_{HH}$ = 1.9 Hz, 1H, 7-C**H**), 7.47 (ddd, $^3J_{HH}$ = 7.4, $^3J_{HH}$ = 5.8, $^4J_{HH}$ = 1.3 Hz 1H, 2-C**H**), 7.30 (dd, $^3J_{HH}$ = 6.2, $^4J_{HH}$ = 1.9 Hz, 1H, 9-C**H**), 7.16 (d, $^3J_{HH}$ = 8.6 Hz, 2H, 21,21′-C**H**), 6.91 (d, J_{HH} = 8.7 Hz, 2H, 22,22′-C**H**), 6.86 (d, $^3J_{HH}$ = 8.7 Hz, 2H, 16,16′-C**H**), 6.66 (d, $^3J_{HH}$ = 8.7 Hz, 2H, 17,17′-C**H**), 3.85 (s, 3H, 24-C**H**$_3$), 3.69 (s, 3H, 19-C**H**$_3$), 2.67 (q, $^3J_{HH}$ = 7.4 Hz, 2H, 12-C**H**$_2$), 1.03 (t, $^3J_{HH}$ = 7.4 Hz, 3H, 13-C**H**$_3$). **^{13}C{^{1}H} NMR** (101 MHz, $CDCl_3$), δ (ppm): 159.3 (23-**C**), 159.1 (18-**C**), 157.2 (8-**C**), 156.0 (6-**C**), 155.3 (5-**C**), 149.6 (1-**C**H), 148.9 (10-**C**H), 144.6 (14-**C**), 139.2 (3-**C**H), 136.2 (20-**C**), 134.3 (15-**C**), 133.8 (11-**C**), 132.2 (16,16′-**C**H), 130.4 (21,21′-**C**H), 127.6 (9-**C**H), 126.7 (2-**C**H), 124.2 (7-**C**H), 122.3 (4-**C**H), 113.9 (22,22′-**C**H), 113.8 (17,17′-**C**H), 55.3 (24-**C**H$_3$), 55.2 (19-**C**H$_3$), 27.7 (12-**C**H$_3$), 13.8 (13-**C**H$_2$). **^{1}H** NMR (400 MHz, DMSO-d_6), δ (ppm): 9.47 (d, $^3J_{HH}$ = 5.8 Hz, 1H, 1-C**H**), 9.14 (d, $^3J_{HH}$ = 6.2 Hz, 1H, 10-C**H**), 8.50 (d, $^3J_{HH}$ = 8.1 Hz, 1H, 4-C**H**), 8.43 (d, $^3J_{HH}$ = 1.9 Hz, 1H, 7-C**H**), 8.38 (t, $^3J_{HH}$ = 7.8 Hz, 1H, 3-C**H**), 7.82 (t, $^3J_{HH}$ = 6.8 Hz, 1H, 2-C**H**), 7.40 (dd, $^3J_{HH}$ = 6.2, $^4J_{HH}$ = 1.9 Hz, 1H, 9-C**H**), 7.16 (d, $^3J_{HH}$ = 8.6 Hz, 2H, 16,16′-C**H**), 6.99 (d, $^3J_{HH}$ = 8.6 Hz, 2H, 17,17′-C**H**), 6.85 (d, $^3J_{HH}$ = 8.6 Hz, 2H, 21,21′-C**H**), 6.70 (d, $^3J_{HH}$ = 8.5 Hz, 2H, 22,22′-C**H**), 3.79 (s, 3H, 24-C**H**$_3$), 3.63 (s, 3H, 19-C**H**$_3$), 2.62 (q, $^3J_{HH}$ = 7.3 Hz, 2H, 12-C**H**$_2$), 0.95 (t, $^3J_{HH}$ = 7.4 Hz, 3H, 13-C**H**$_3$). **Elemental analysis:** $C_{28}H_{26}Cl_2N_2O_2Pt$ calc. (%) C 47.85 H 3.81 N 4.07, found (%) C 47.10 H 3.67 N 3.98; **UV-vis:** λ_{max} = 240, 300, 430 nm.

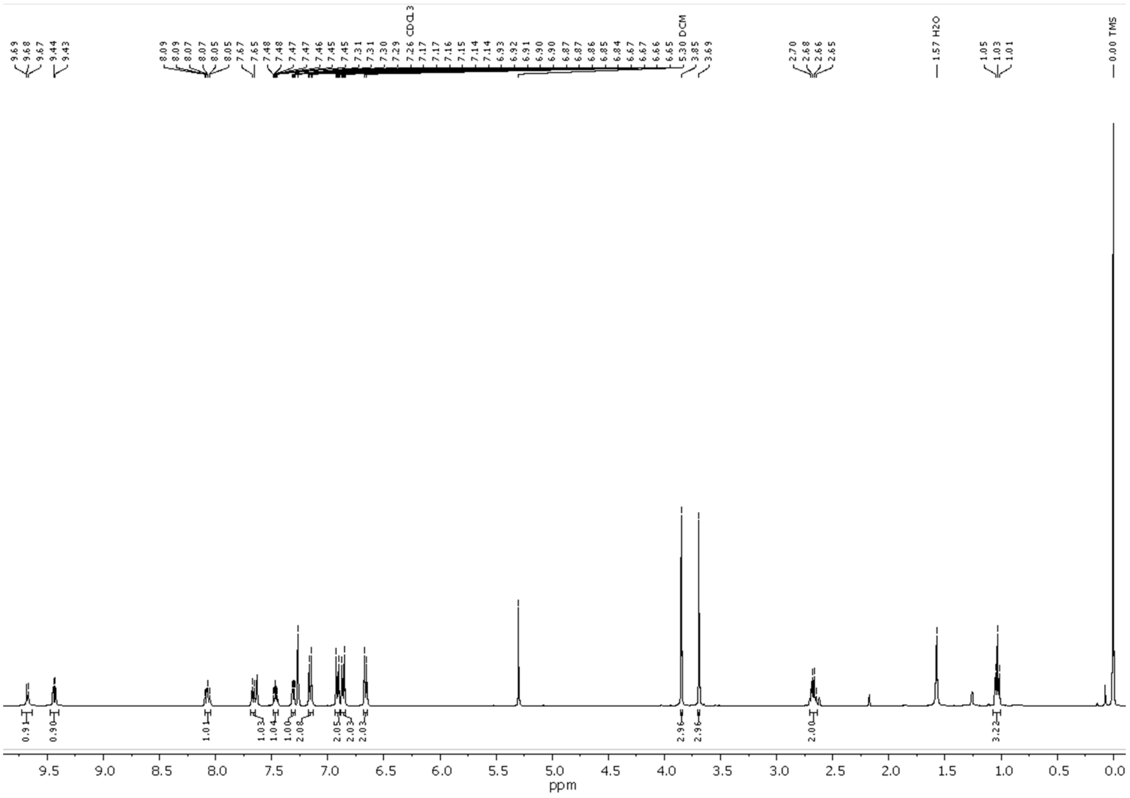

Figure S5. ^{1}H NMR spectrum of compound **5** in $CDCl_3$.

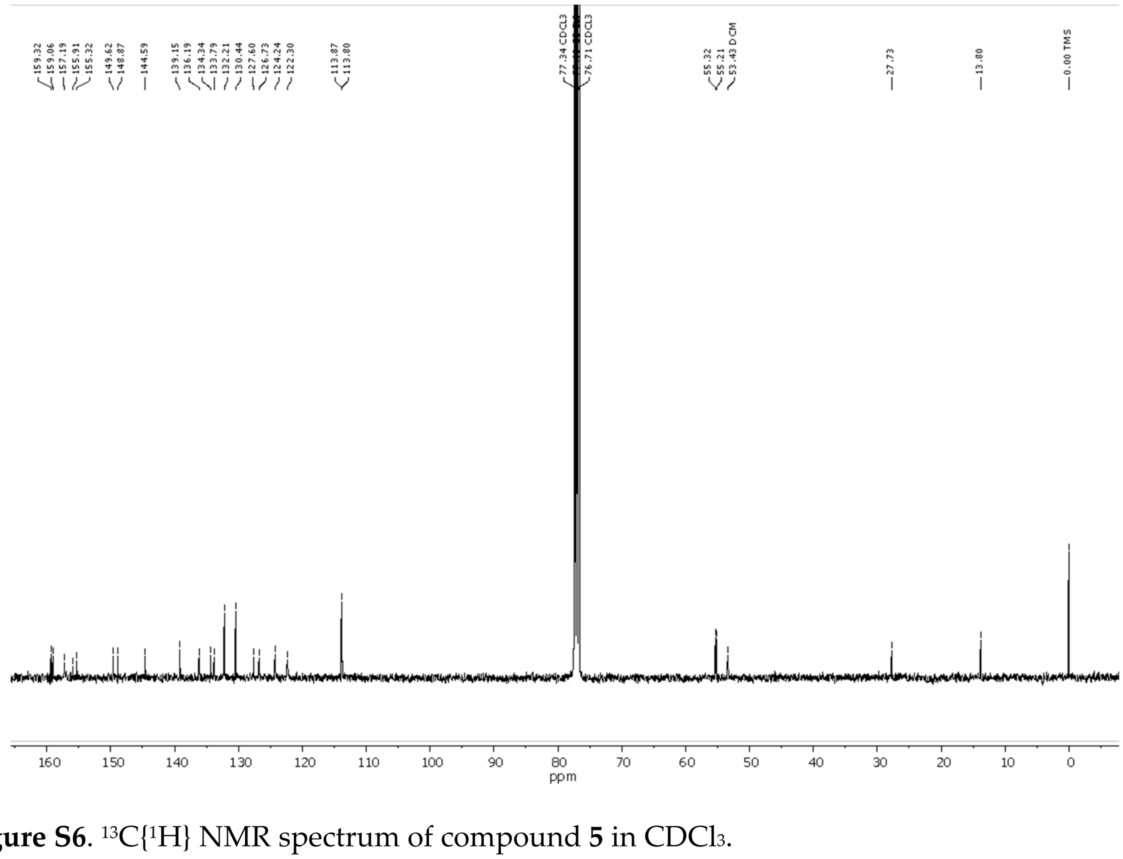

Figure S6. $^{13}C\{^{1}H\}$ NMR spectrum of compound **5** in $CDCl_3$.

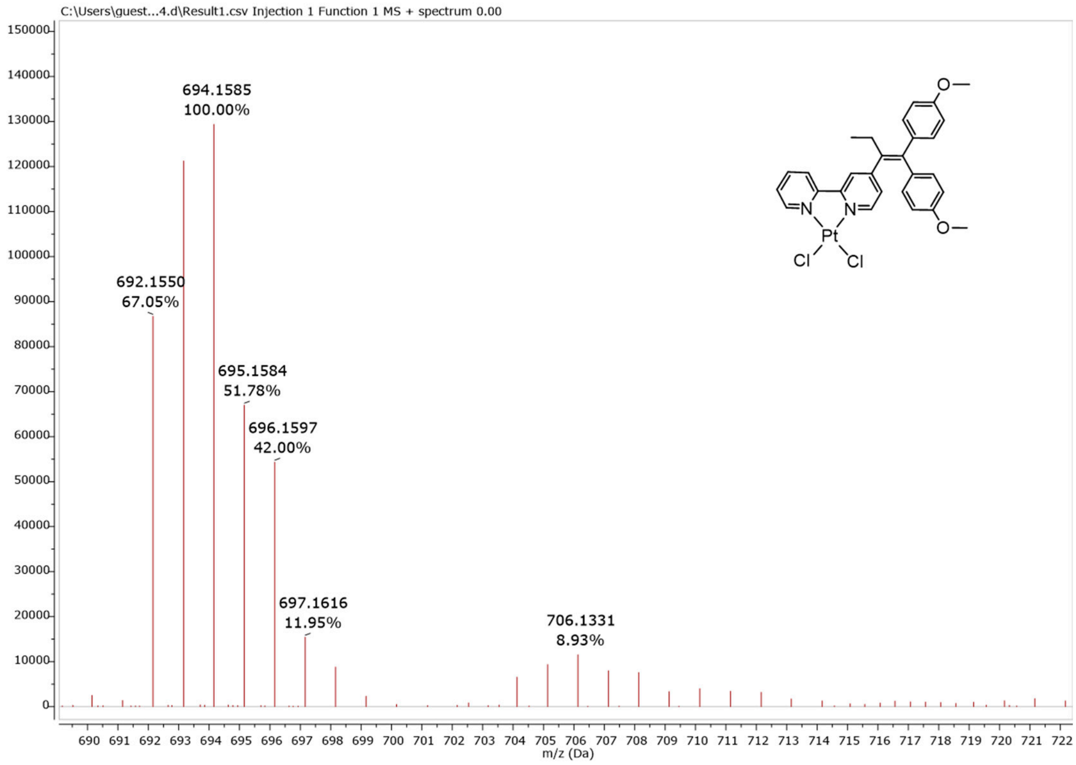

Figure S7. HR-ESI-MS (Positive mode, acetonitrile (MeCN)) of **5**: m/z $[M+NH_4]^+$ = 706.1363 (calc.), 706.1331 (found); m/z $[M\text{-}Cl+MeCN]^+$ = 694.1596 (calc.), 694.1585 (found).

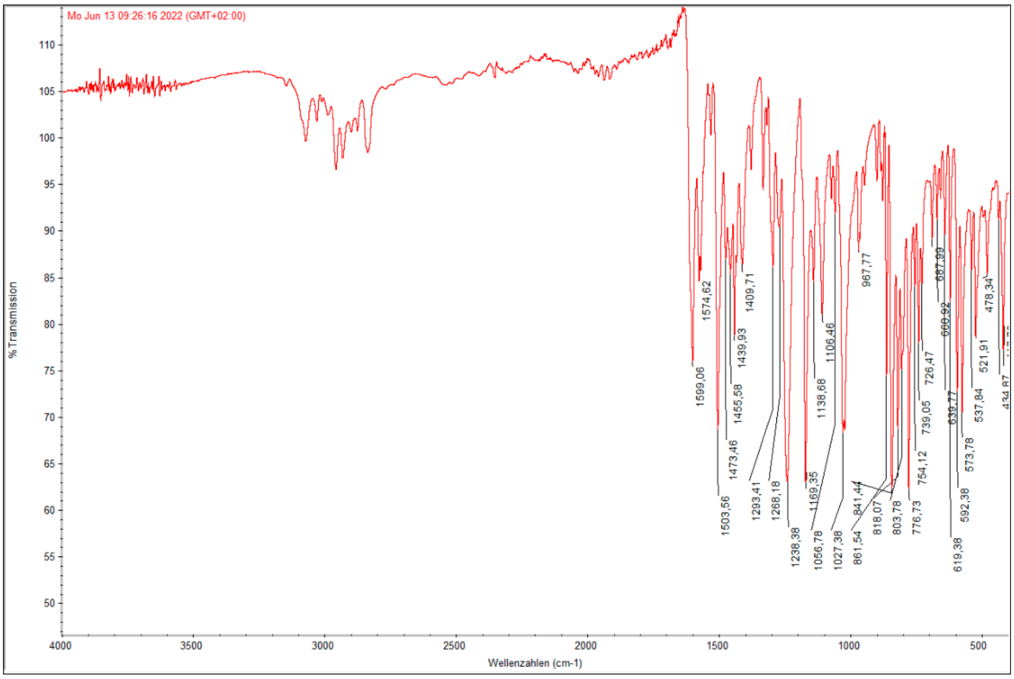

Figure S8. IR spectrum of **5**: 3071−2084 (w, C_{alk}−H), 1599 (m, ν(C=C)), 1575 (m), 1504 (s, C_{arom}−H in-plane bending), 1473 (w, C_{arom}-H in-plane binding), 1456 (w, C_{arom}−H in-plane bending), 1440 (m, C_{arom}−H in-plane bending), 1409 (w), 1293 (w), 1268 (w, C_{arom}−O), 1238 (s, C_{arom}-O), 1169 (s, C_{arom}−O), 1107 (m,), 1027 (s, C_{alk}−O), 841 (m, out-of-plane bending), 804 (m, out-of-plane bending) 776 (s, out-of-plane bending), 739 (w), 661 (w), 574 (w), 522 (w), 435 (w).

*1.3. [$PdCl_2$(L-κ²N,N′)] (**6**)*

M($C_{28}H_{26}Cl_2N_2O_2Pd$) = 598.04 g mol⁻¹, **¹H NMR** (400 MHz, $CDCl_3$), δ (ppm): 9.34 (dd, $^3J_{HH}$ = 5.8, $^3J_{HH}$ = 1.5 Hz, 1H, 1-C**H**), 9.11 (d, $^3J_{HH}$ = 6.0 Hz, 1H, 10-C**H**), 8.03 (td, $^3J_{HH}$ = 7.8 Hz, $^3J_{HH}$ = 1.6 Hz, 1H, 3-C**H**), 7.69 (d, $^3J_{HH}$ = 8.2 Hz, 1H, 4-C**H**), 7.65 (d, $^3J_{HH}$ = 1.9 Hz, 1H, 7-C**H**), 7.47 (ddd, $^3J_{HH}$ = 7.4, $^3J_{HH}$ = 5.7, $^4J_{HH}$ = 1.3 Hz, 1H, 2-C**H**), 7.30 (dd, $^3J_{HH}$ = 6.1 Hz, $^4J_{HH}$ = 1.9 Hz, 1H, 9-C**H**), 7.15 (d, $^3J_{HH}$ = 8.6, 2H, 21,21′-C**H**), 6.91 (d, $^3J_{HH}$ = 8.7 Hz, 2H, 22,22′-C**H**), 6.84 (d, $^3J_{HH}$ = 8.4 Hz, 2H, 16,16′-C**H**), 6.66 (d, $^3J_{HH}$ = 8.4 Hz, 2H, 17,17′-C**H**), 3.84 (s, 3H, 24-C**H**$_3$), 3.70 (s, 3H, 19-C**H**$_3$), 2.66 (q, $^3J_{HH}$ = 7.4 Hz, 2H, 12-C**H**$_2$), 1.02 (t, $^3J_{HH}$ = 7.4 Hz, 3H, 13-C**H**$_3$).**¹³C{¹H} NMR** (101 MHz, $CDCl_3$), δ (ppm): 159.4 (23-**C**), 159.1 (18-**C**), 156.5 (5-**C**), 156.3 (6-**C**), 155.2 (8-**C**), 151.1 (1-**C**H), 150.4 (10-**C**H), 144.9 (14-**C**), 140.0 (3-**C**H), 136.0 (20-**C**), 134.2 (15-**C**), 133.7 (11-**C**H), 132.2 (16,16′-**C**H), 130.5 (21,21′-**C**H), 127.3 (9-**C**H), 126.4 (2-**C**H), 123.9 (7-**C**H), 121.9 (4-**C**H), 113.8 (22,22′-**C**H), 113.8 (17, 17′-**C**H) 55.3 (24-**C**H$_3$), 55.2 (19-**C**H$_3$), 27.9 (12-**C**H$_2$), 13.8 (13-**C**H$_3$). **¹H NMR** (400 MHz, DMSO-d_6), δ (ppm): 9.47 (d, $^3J_{HH}$ = 5.8 Hz, 1H, 1-C**H**), 9.14 (d, $^3J_{HH}$ = 6.2 Hz, 1H, 10-C**H**), 8.50 (d, $^3J_{HH}$ = 8.1 Hz, 1H, 4-C**H**), 8.43 (d, $^3J_{HH}$ = 1.9 Hz, 1H, 7-C**H**), 8.38 (t, $^3J_{HH}$ = 7.8 Hz, 1H, 3-C**H**), 7.82 (t, $^3J_{HH}$ = 6.8 Hz, 1H, 2-C**H**), 7.40 (dd, $^3J_{HH}$ = 6.2, $^4J_{HH}$ = 1.9 Hz, 1H, 9-C**H**), 7.15 (d, $^3J_{HH}$ = 8.6 Hz, 2H, 21,21′-C**H**), 6.99 (d, $^3J_{HH}$ = 8.1 Hz, 2H, 22,22′-C**H**), 6.85 (d, $^3J_{HH}$ = 8.6 Hz, 2H, 16,16′-C**H**), 6.70 (d, $^3J_{HH}$ = 8.6 Hz, 1H, 17,17′-C**H**), 3.79 (s, 3H, 24-C**H**$_3$), 3.63 (s, 3H, 19-C**H**$_3$), 2.62 (q, $^3J_{HH}$ = 7.5 Hz, 1H, 12-C**H**$_2$), 0.95 (t, $^3J_{HH}$ = 7.4 Hz, 3H, 13-C**H**$_3$), **Elemental analysis:** $C_{28}H_{26}Cl_2N_2O_2Pd$ calc. (%) C 56.07 H 4.37 N 4.67, found (%) C 55.74 H 4.77 N 4.36; UV-vis: λ_{max} = 245, 290, 400 nm

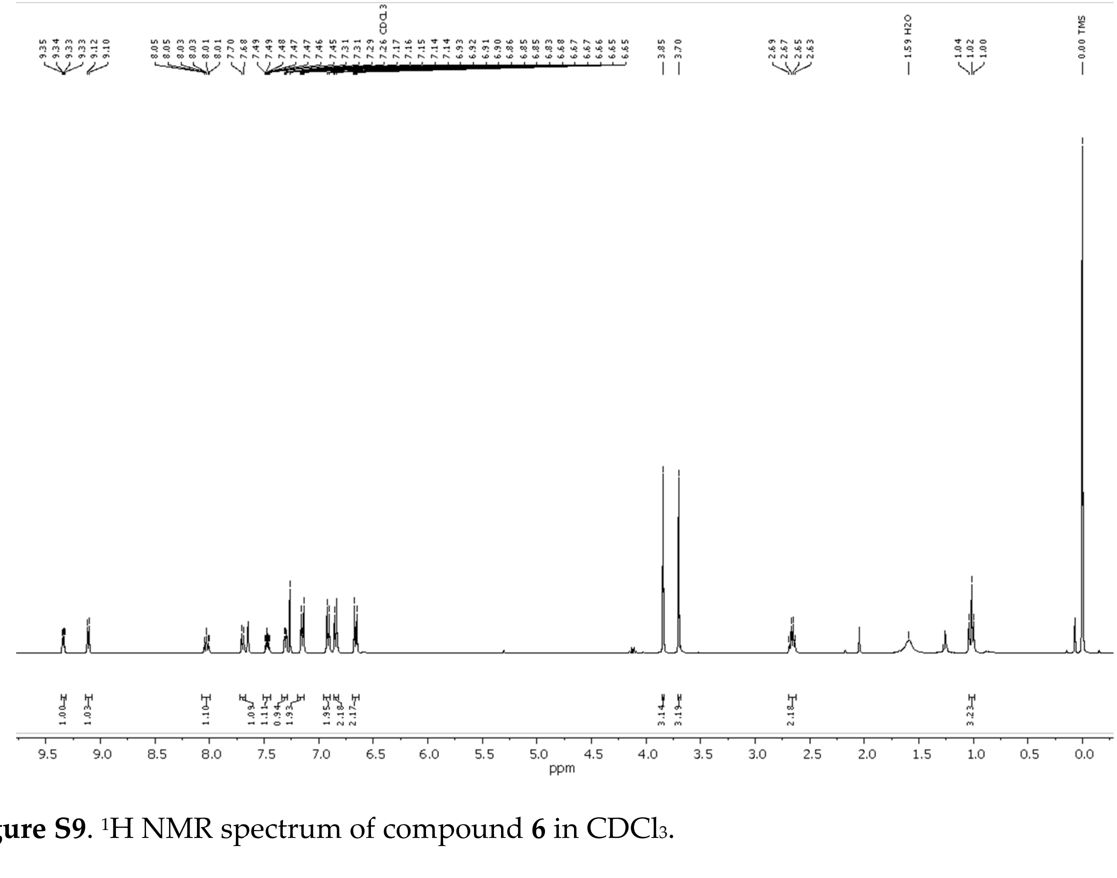

Figure S9. ¹H NMR spectrum of compound **6** in $CDCl_3$.

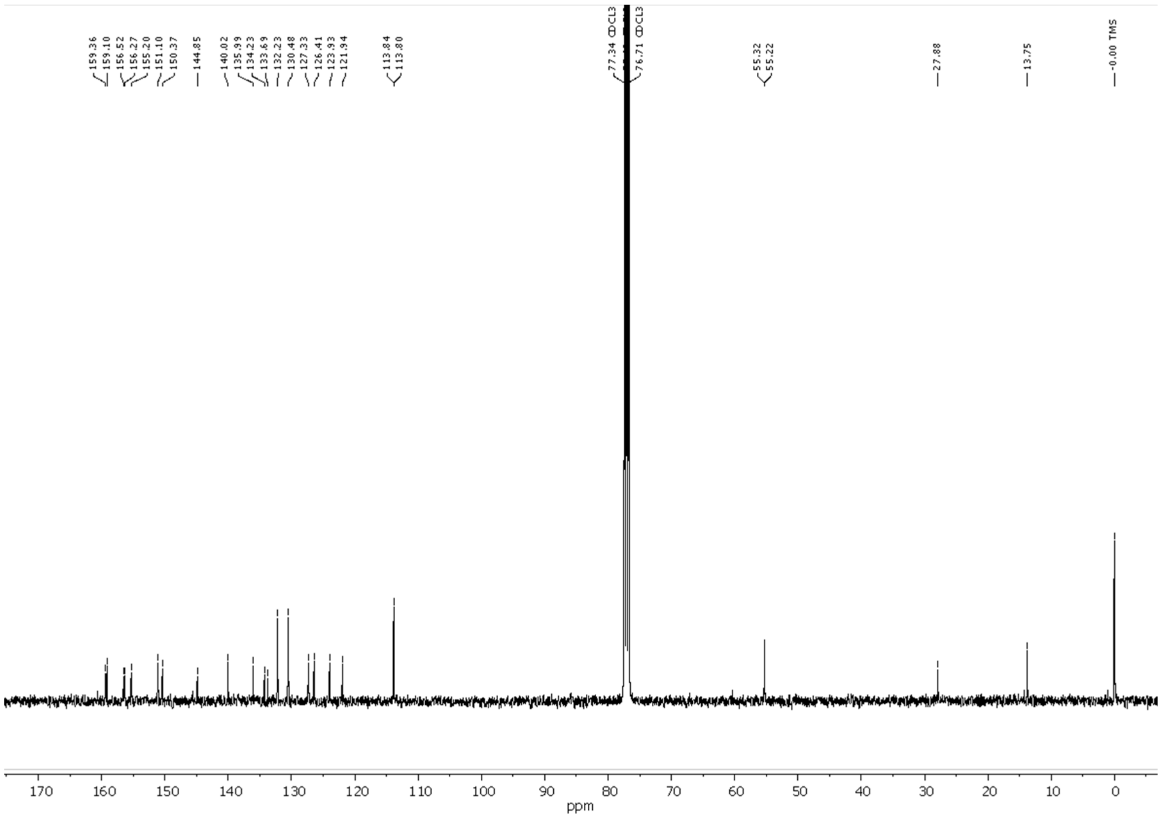

Figure S10. $^{13}C\{^{1}H\}$ NMR spectrum of compound **6** in $CDCl_3$.

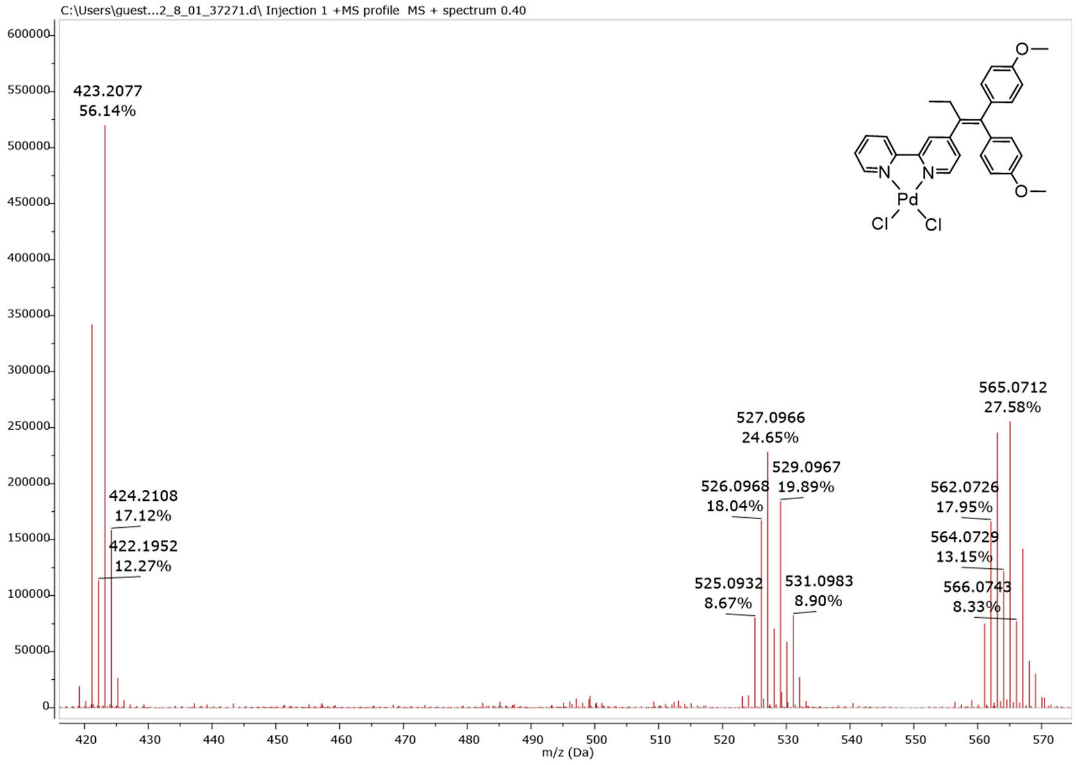

Figure S11. HR-ESI-MS (positive mode, acetonitrile) of **6**: m/z $[M{-}Cl]^+$ = 565.0722 (calc.), 565.0712 (found); m/z $[M{-}2Cl]^+$ = 527.1045 (calc.), 527.0966 (found); m/z $[C_{28}H_{26}N_2O_2{+}H]^+$ = 423.2073 (calc.), 423.2077 (found).

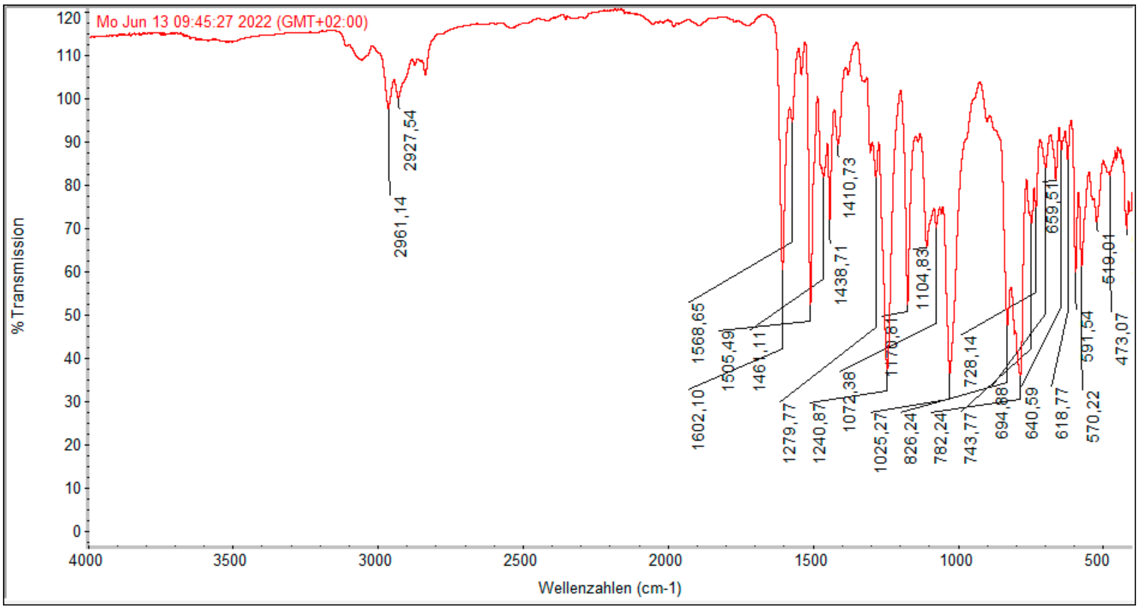

Figure S12. IR spectrum of **6**: 2961 (w, C_{alk}–H), 2927 (w, C_{alk}–H), 1602 (m, v(C=C)), 1569 (w), 1506 (m, C_{arom}–H in-plane bending), 1461 (w, C_{arom}–H in-plane bending), 1439 (m, C_{arom}–H in-plane bending), 1411(w), 1279 (w), 1241 (m, C_{arom}–O), 1171 (m, C_{arom}–O), 1105 (w), 1025 (m, C_{alk}–O), 826 (m, C_{arom}–H out-of-plane bending), 782 (m, C_{arom}–H out-of-plane bending), 744 (w), 660 (w), 619 (w), 591 (w), 570 (w), 519 (w), 473 (w).

1.4. [3-(L-κ²N,N′)-3,1,2-PtC₂B₉H₁₁] (**7**)

R_f = 0.25 (DCM/*n*-hexane, 3:2), M($C_{30}H_{37}N_2O_2PtB_9$) = 751.33 g mol^{-1}. **^{1}H NMR** (400 MHz, CD_2Cl_2), δ (ppm): 9.12 (d, $^3J_{HH}$ = 5.7 Hz, 1H, 1-C**H**), 8.88 (d, $^3J_{HH}$ = 5.9 Hz, 1H, 10-C**H**), 8.02 (t, $^3J_{HH}$ = 7.8, $^3J_{HH}$ = 1.5 Hz, 1H, 3-C**H**), 7.88 (dd, $^3J_{HH}$ = 5.1, $^3J_{HH}$ = 3.2 Hz, 2H, 4-C**H** and 7-C**H**), 7.45 (t, $^3J_{HH}$ = 6.7 Hz, 1H, 2-C**H**), 7.27 (dd, $^3J_{HH}$ = 5.9, $^4J_{HH}$ = 1.9 Hz, 1H, 9-C**H**), 7.18 (d, $^3J_{HH}$ = 8.7 Hz, 2H, 21,21′-C**H**), 6.91 (dd, $^3J_{HH}$ = 12.7, $^3J_{HH}$ = 8.6 Hz, 4H, 22, 22′- and 16,16′-C**H**), 6.67 (d, ^3J = 8.7 Hz, 2H, 17,17′-C**H**), 3.90 (br s, 2H, C**H**$_{cluster}$), 3.83 (s, 3H, 24-C**H**$_3$), 3.68 (s, 3H, 19-C**H**$_3$), 2.66 (q, $^3J_{HH}$ = 7.4 Hz, 2H, 12-C**H**$_2$), 1.02 (t, $^3J_{HH}$ = 7.4 Hz, 3H, 13-C**H**$_3$), 2.32–1.03 (m, 9H, B**H**$_{cluster}$).**^{13}C{^{1}H} NMR** (101 MHz, CD_2Cl_2), δ (ppm): 159.7 (23-**C**), 159.4 (18-**C**), 154.9 (5-**C**), 154.4 (6-**C**), 153.5 (8-**C**), 153.3 (1-CH), 152.8 (10-CH), 144.2 (14-**C**), 138.9 (3-CH), 137.0 (20-C), 135.1 (15-**C**), 134.5 (11-CH), 132.5 (21,21′-C**H**), 130.8 (16,16′-C**H**), 128.0 (9-C**H**), 127.0 (2-C**H**), 124.9 (7-C**H**), 122.8 (4-C**H**), 114.1 (17,17′-C**H** and 22,22′-C**H**), 59.1 (C**H**$_{cluster}$), 55.7 (24-C**H**$_3$), 55.6 (19-C**H**$_3$), 28.2 (12-C**H**$_2$), 13.8 (13-C**H**$_2$). **^{11}B** NMR (128 MHz, CD_2Cl_2), δ (ppm): 15.5 (d, *J* = 147 Hz, 1B), −10.6 (m, 5B), −22.4 (d, *J* = 167 Hz, 1B), −28.6 (d, *J* = 168 Hz, 2B). **^{1}H NMR** (400 MHz, DMSO-d_6), δ (ppm): 8.94 (d, $^3J_{HH}$ = 5.4 Hz, 1H, 1-C**H**), 8.67 (s, 1H, 7-C**H**), 8.65 (d, $^3J_{HH}$ = 2.9 Hz, 1H, 10-C**H**), 8.59 (d, $^3J_{HH}$ = 1.9 Hz, 1H, 4-C**H**), 8.35 (d, $^3J_{HH}$ = 2.9 Hz, 1H, 3-C**H**), 7.69 (ddd, $^3J_{HH}$ = 7.4, $^3J_{HH}$ = 5.6, $^4J_{HH}$ = 1.3 Hz, 1H, 2-C**H**), 7.30 (dd, $^3J_{HH}$ = 5.9, $^4J_{HH}$ = 1.8 Hz, 1H, 9-C**H**), 7.16 (d, $^3J_{HH}$ = 8.7 Hz, 2H, 21,21′-C**H**), 6.99 (d, *J* = 8.7 Hz, 2H, 22,22′-C**H**), 6.88 (d, *J* = 8.7 Hz, 2H, 16,16′-C**H**), 6.71 (d, *J* = 8.8 Hz, 2H, 17,17′-C**H**), 3.99 (br s, 2H, C**H**$_{cluster}$), 3.79 (s, 3H, 24-C**H**$_3$), 3.63 (s, 3H, 3H, 19-C**H**$_3$), 2.63 (q, $^3J_{HH}$ = 7.5 Hz, 2H, 12-C**H**$_2$), 0.95 (t, $^3J_{HH}$ = 7.4 Hz, 3H, 13-C**H**$_3$), 0.40 – 2.5 (m, 9-B**H**). **Elemental analysis:** $C_{30}H_{37}N_2O_2PtB_9$ calc. (%) C 48.04 H 4.97 N 3.74, found (%) C 47.68 H 5.26 N 3.93; **UV-vis:** λ_{max} = 240, 300, 450 nm.

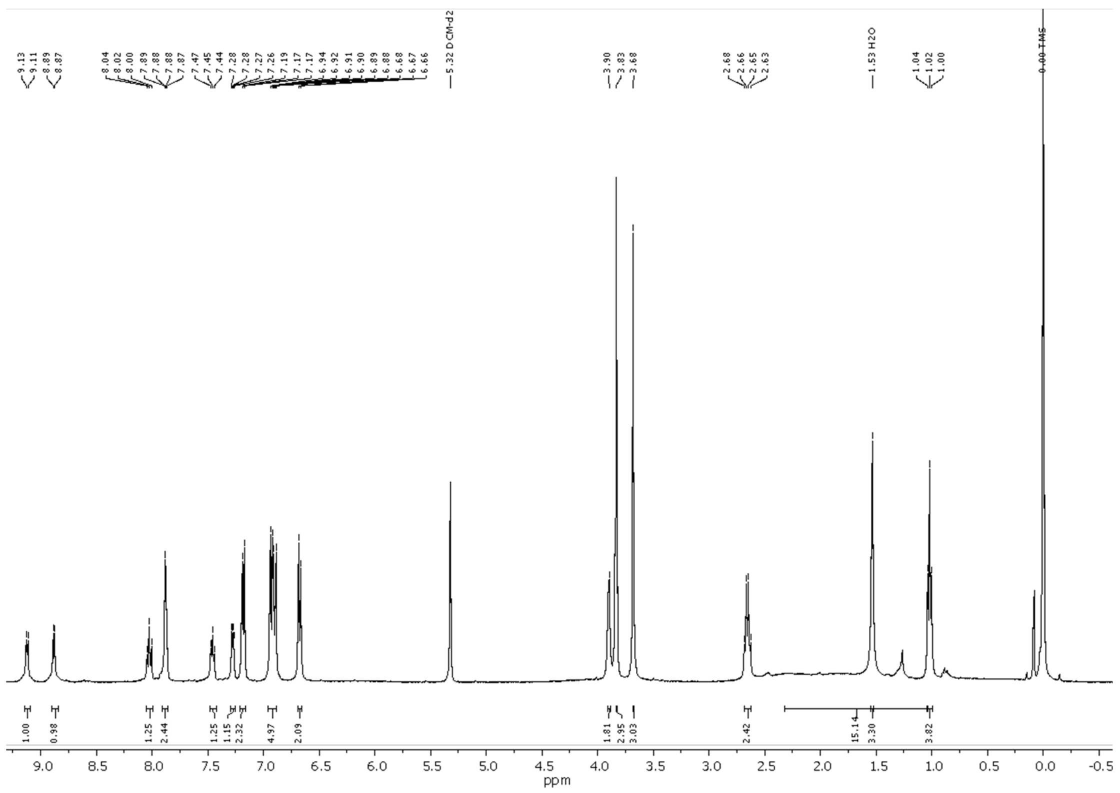

Figure S13. ^{1}H NMR spectrum of compound **7** in CD_2Cl_2.

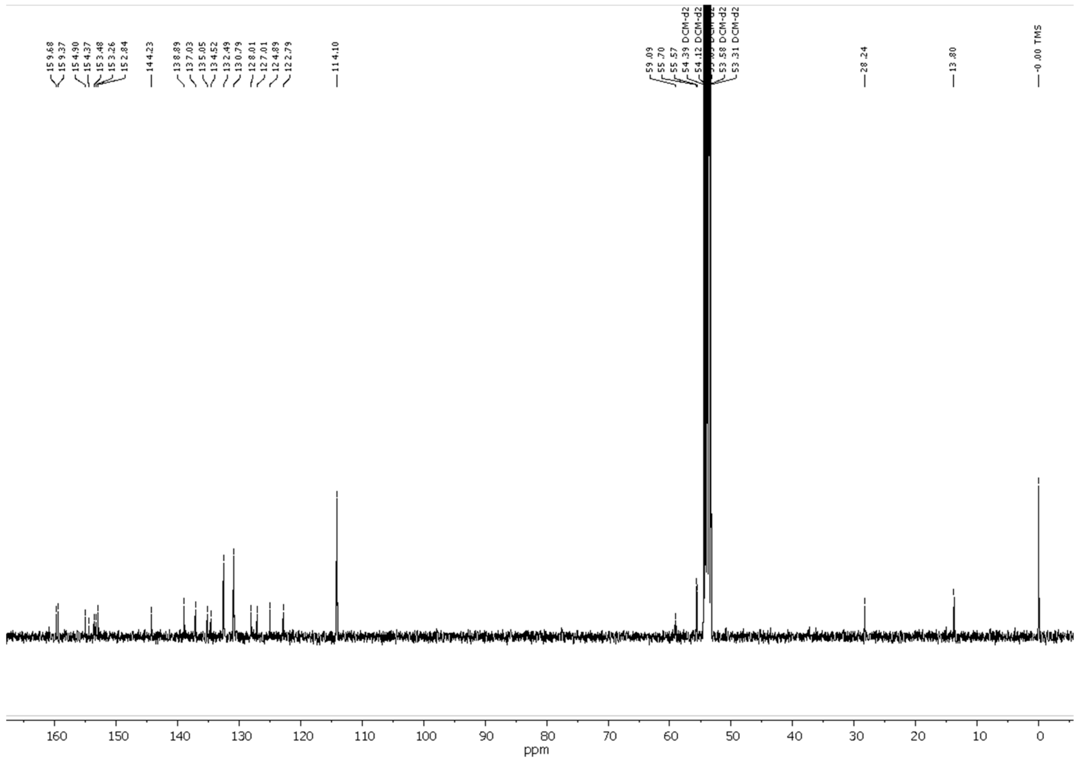

Figure S14. $^{13}C\{^{1}H\}$ NMR spectrum of compound **7** in CD_2Cl_2.

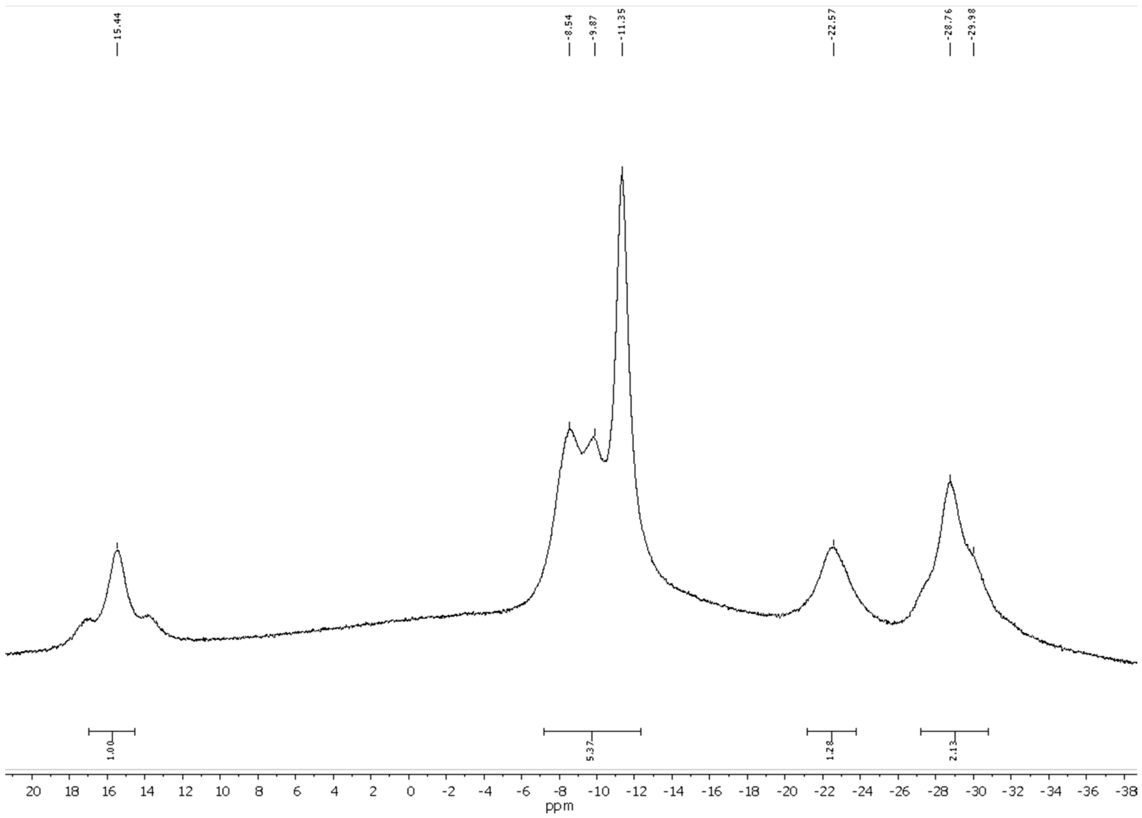

Figure S15. $^{11}B\{^{1}H\}$ NMR spectrum of compound **7** in CD_2Cl_2.

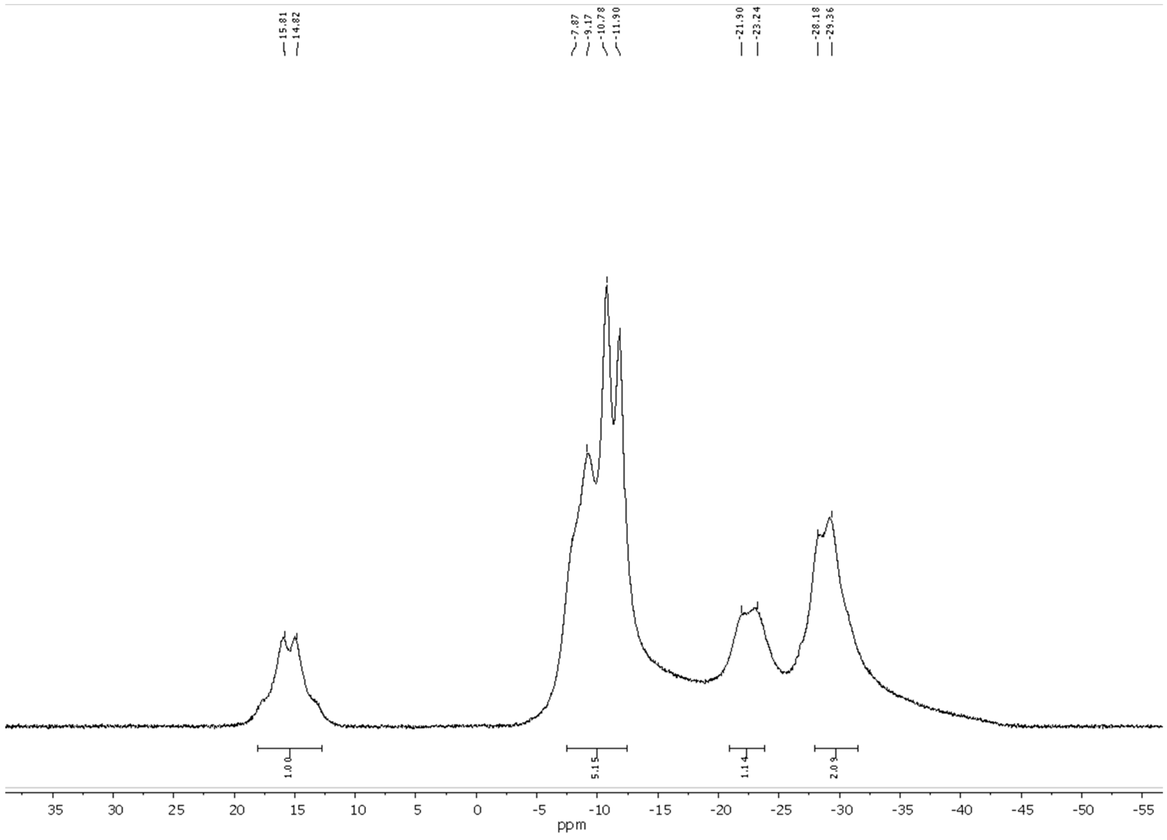

Figure S16. ^{11}B NMR spectrum of compound **7** in CD_2Cl_2.

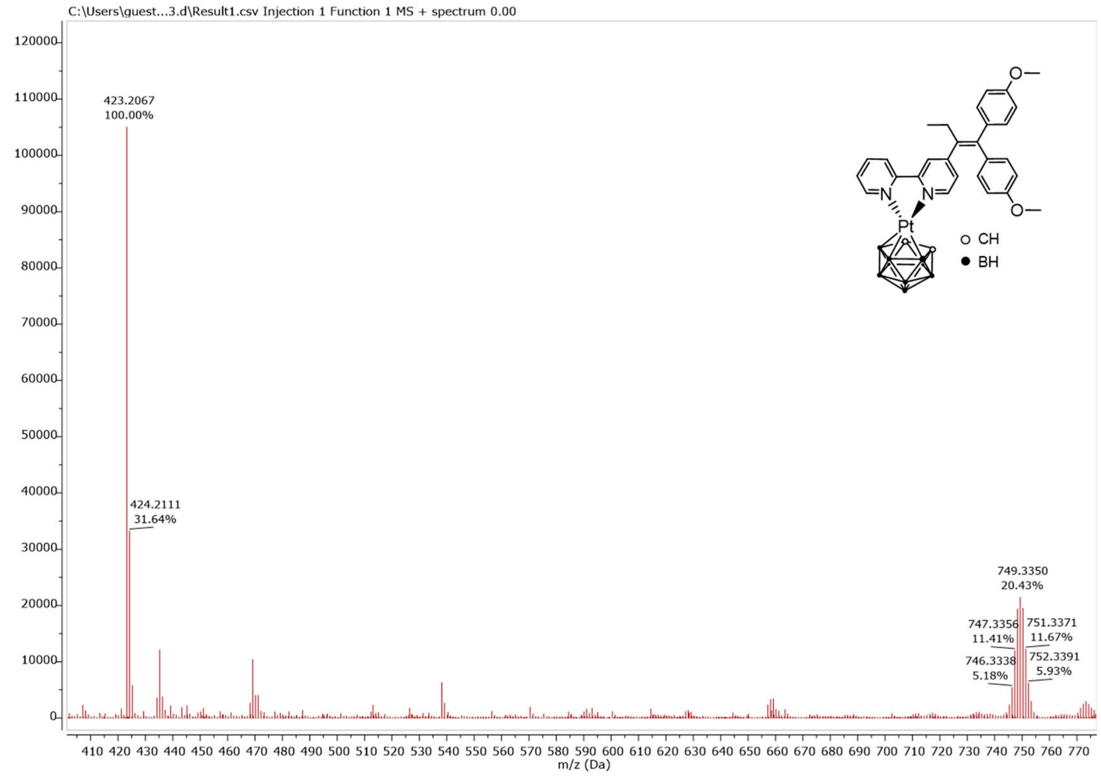

Figure S17. HR-ESI-MS (positive mode, acetonitrile) of **7**: m/z $[M+H]^+$ = 749.3340 (calc.), 749.3350 (found); m/z $[C_{28}H_{26}N_2O_2+H]^+$ = 423.2073 (calc.), 423.2067 (found).

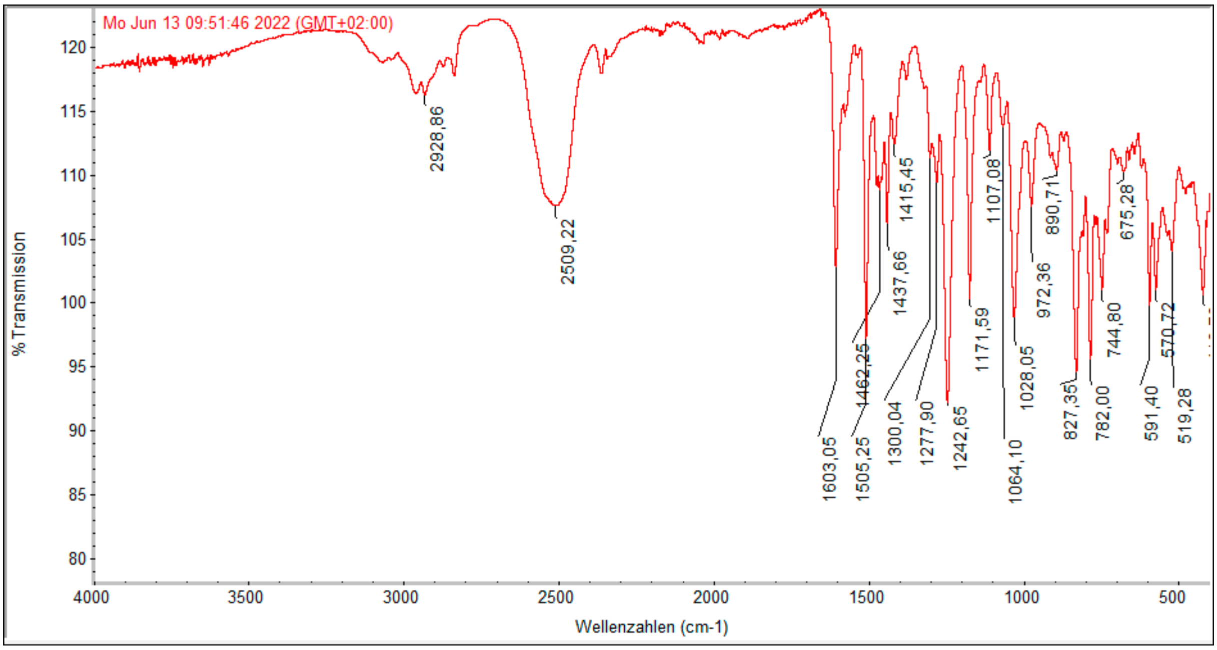

Figure S18. IR spectra of **7**: 2961(w, C_{alk}-H), 2929 (w, C_{alk}-H), 2509 (m, v(B-H)), 1603 (m, v(C=C)), 1505 (m, C_{arom}-H in-plane bending), 1462 (w, C_{arom}-H in-plane bending), 1438 (m, C_{arom}-H in-plane bending), 1277 (w), 1243 (s, C_{arom}-O), 1173 (m, C_{arom}-O), 1028 (m, C_{alk}-O), 826 (m, C_{arom}-H out-of-plane bending), 782 (m, C_{arom}-H out-of-plane bending), 591 (w), 520 (w).

1.5. [3-(L-κ²N,N′)-3,1,2-$PdC_2B_9H_{11}$] (8)

R_f = 0.40 (DCM/*n*-hexane 3:1), M($C_{30}H_{37}N_2O_2PdB_9$) = 662.27 g mol⁻¹. **¹H NMR** (400 MHz, $CDCl_3$), δ (ppm): 8.66 (d, $^3J_{HH}$ = 5.6, 1H, 1-C**H**), 8.45 (d, $^3J_{HH}$ = 5.7 Hz, 1H, 10-C**H**), 7.94 (t, $^3J_{HH}$ = 7.8 Hz, 1H, 3-C**H**), 7.83 (m, 2H, 4-C**H** and 7-C**H**), 7.41 (t, $^3J_{HH}$ = 6.5 Hz, 1H, 2-C**H**), 7.23 (d, $^3J_{HH}$ = 6.0, 1H, 9-C**H**), 7.16 (d, ^3J = 8.6 Hz, 2H, 21,21′-C**H**), 6.92 (d, ^3J = 8.7 Hz, 2H, 22,22′-C**H**), 6.86 (d, ^3J = 8.7 Hz, 2H, 16,16′-C**H**), 6.67 (d, ^3J = 8.7 Hz, 2H, 17,17′-C**H**), 3.84 (s, 3H, 24-C**H**$_3$), 3.76 (br s, 2H, C**H**$_{cluster}$), 3.70 (s, 3H, 19-C**H**$_3$), 2.65 (q, ^3J = 7.4 Hz, 2H, 12-C**H**$_2$), 1.00 (t, ^3J = 7.4 Hz, 3H, 13-C**H**$_3$), 0.80–2.35 (m, 9H, B**H**$_{cluster}$). **¹³C{¹H} NMR** (101 MHz, $CDCl_3$), δ (ppm): 159.2 (23-**C**), 158.9 (18-**C**), 154.4 (5-**C**), 153.5 (6-**C**), 152.4 (8-**C**), 152.4 (1-**C**H), 151.9 (10-**C**H), 143.8 (14-**C**), 138.3 (3-**C**H), 136.4 (20-**C**), 134.5 (15-**C**), 134.0 (11-**C**H), 132.1 (21, 21′-**C**H), 130.4 (16,16′-**C**H), 126.8 (9-**C**), 125.8 (2-**C**H), 124.0 (7-**C**H), 121.9 (4-**C**H), 113.8 (17,17′-**C**H), 113.8 (22,22′-**C**H) 55.3 (24-**C**H), 55.2 (19-**C**H), 49.5 (**C**H$_{cluster}$), 28.0 (12-**C**H), 13.7 (13-**C**H). **¹¹B NMR** (400 MHz, $CDCl_3$), δ (ppm): 17.9 (d, *J* = 134 Hz, 1B, **B**H), −9.7 (d, *J* = 142 Hz, 5B, **B**H), −20.7 (m, 3B, **B**H). **¹H NMR** (400 MHz, DMSO-d_6), δ (ppm): 8.59 (d, $^3J_{HH}$ = 8.2 Hz, 1H, 10-C**H**), 8.53 (d, $^3J_{HH}$ = 1.8 Hz, 1H, 7-C**H**), 8.50 (dd, $^3J_{HH}$ = 5.5, $^3J_{HH}$ = 1.5 Hz, 1H, 1-C**H**), 8.24 (m, 2H, 4-C**H** and 3-C**H**), 7.66 (ddd, $^3J_{HH}$ = 7.3, $^3J_{HH}$ = 5.5, $^4J_{HH}$ = 1.2 Hz, 1H, 2-C**H**), 7.28 (dd, $^3J_{HH}$ = 5.8, $^4J_{HH}$ = 1.8 Hz, 1H, 9-C**H**), 7.16 (d, $^3J_{HH}$ = 8.7 Hz, 2H, 21,21′-C**H**), 6.98 (d, $^3J_{HH}$ = 8.7 Hz, 2H, 22,22′-C**H**), 6.87 (d, $^3J_{HH}$ = 8.8 Hz, 16,16′-C**H**), 6.70 (d, $^3J_{HH}$ = 8.7 Hz, 2H, 17,17′-C**H**), 3.85 (br s, 2H, C**H**$_{cluster}$), 3.78 (s, 3H, 24-C**H**$_3$), 3.62 (s, 3H, 19-C**H**$_3$), 2.61 (q, $^3J_{HH}$ = 7.3 Hz, 2H, 12-C**H**$_2$), 0.93 (t, $^3J_{HH}$ = 7.4 Hz, 3H, 13-C**H**$_3$), 0.30–2.24 (m, 9H, B**H**$_{cluster}$). **Elemental analysis:** $C_{30}H_{37}N_2O_2PdB_9$ Calc. (%) C 54.49 H 5.64 N 4.24 Found (%) C 54.15 H 5.60 N 3.84; **UV-vis:** λ_{max} = 240, 300, 420 nm.

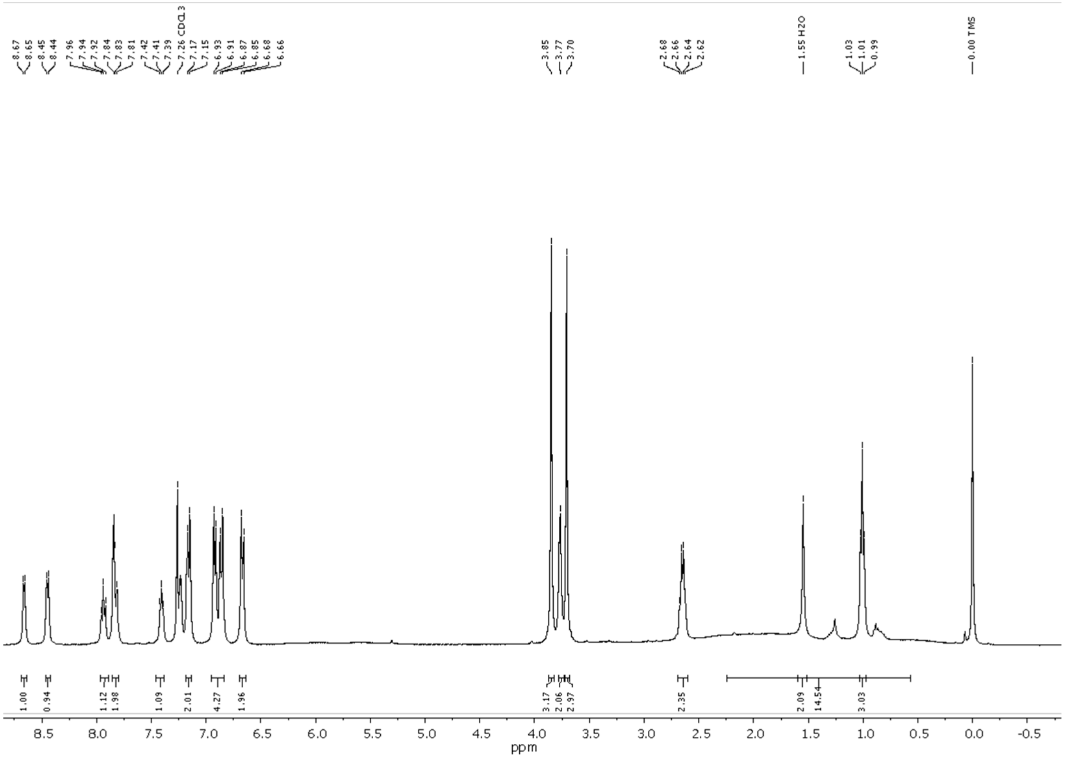

Figure S19. ¹H NMR spectrum of compound **8** in $CDCl_3$.

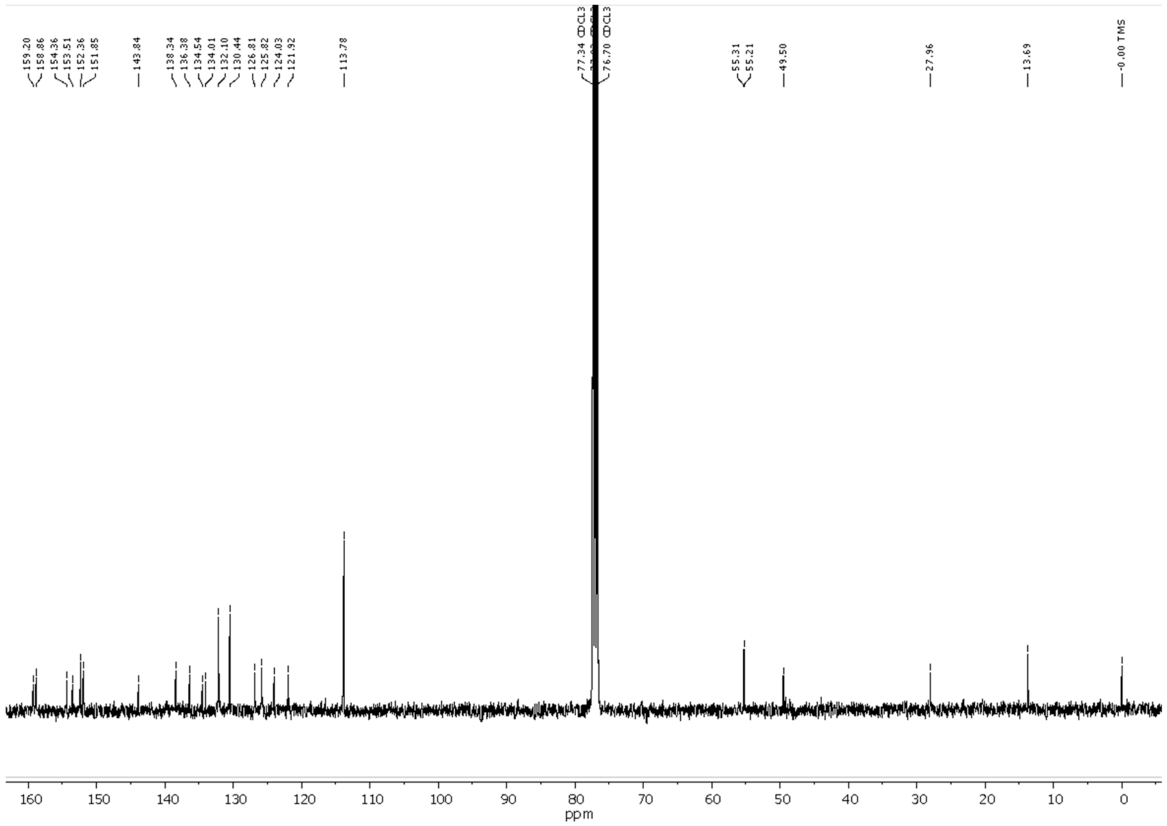

Figure S20. $^{13}C\{^1H\}$ NMR spectrum of compound **8** in $CDCl_3$.

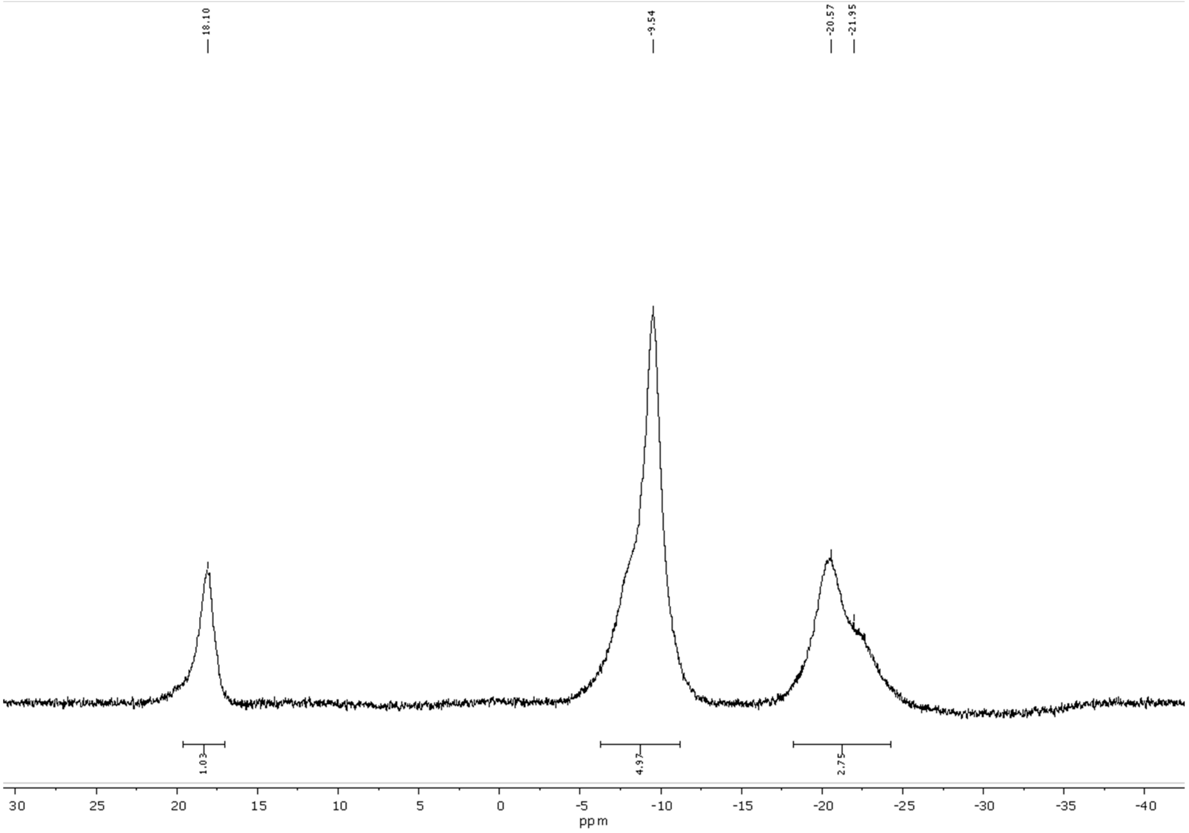

Figure S21. $^{11}B\{^1H\}$ NMR spectrum of compound **8** in $CDCl_3$.

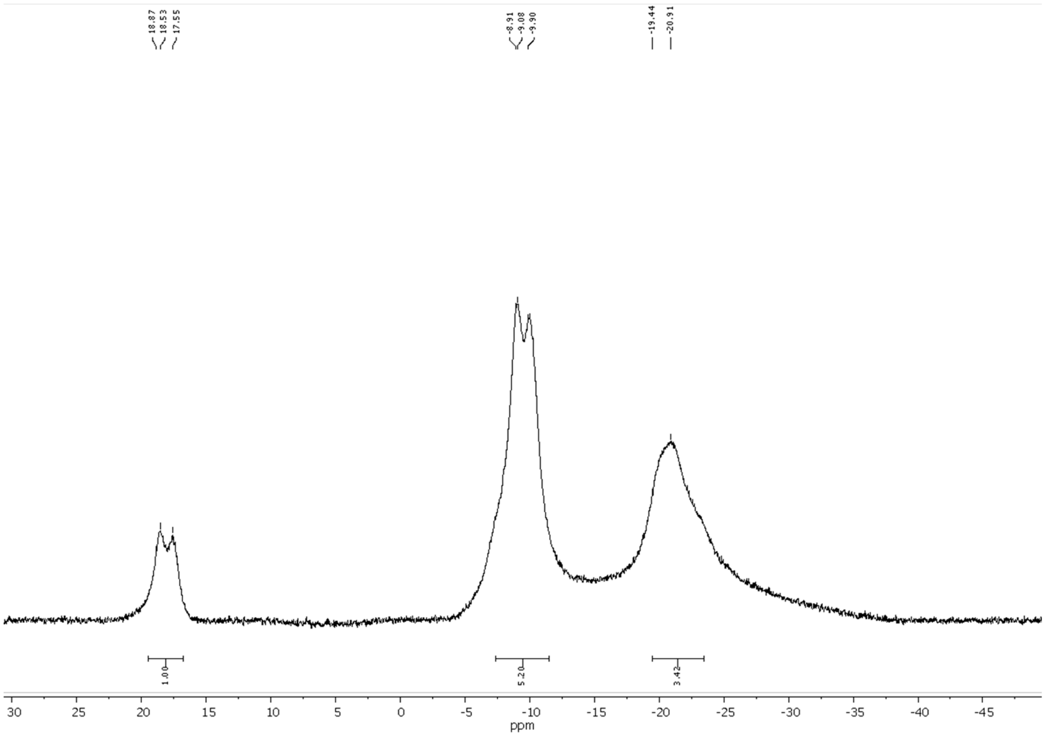

Figure S22. ^{11}B NMR spectrum of compound **8** in $CDCl_3$.

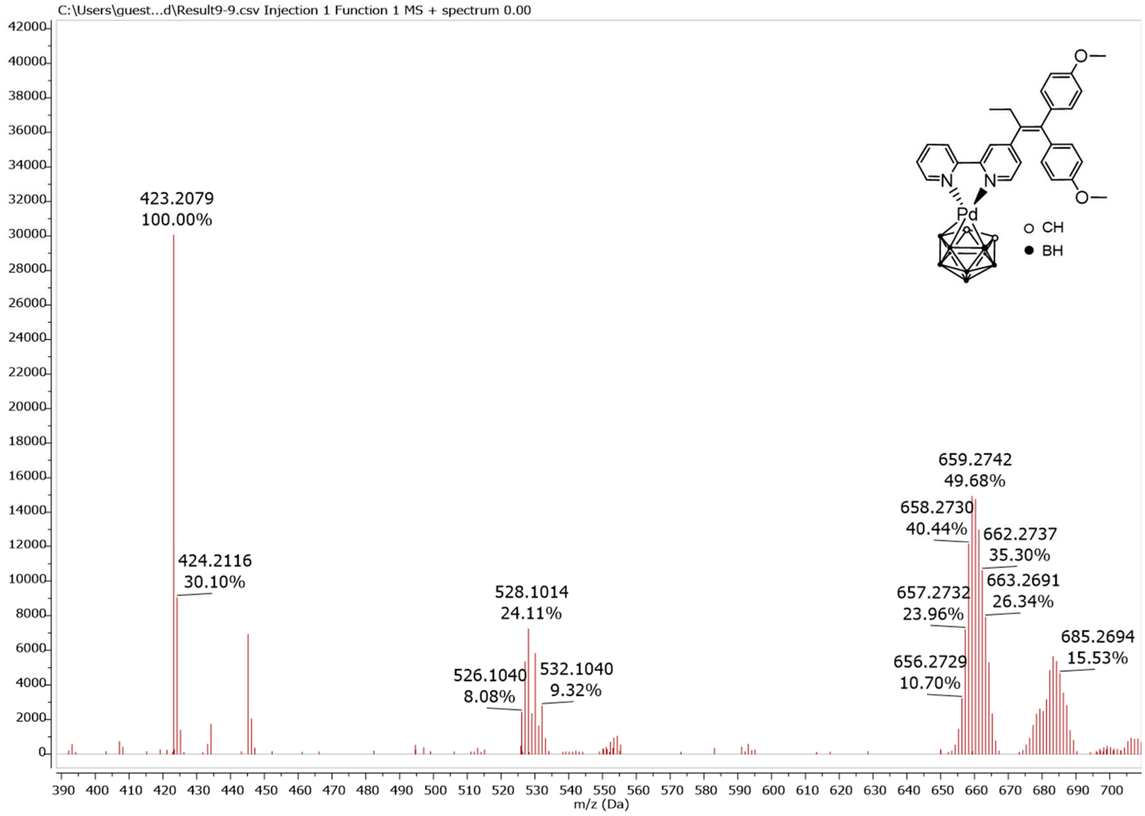

Figure S23. HR-ESI-MS (positive mode, acetonitrile) of **8**: m/z $[M+Na]^+$ = 685.2625 (calc.), 685.2694 (found); m/z $[M+H]^+$ = 659.2826 (calc.), 659.2742 (found); m/z $[M-C_2B_9H_{11}]^+$ = 528.1029 (calc.), 528.1014 (found), m/z $[C_{28}H_{26}N_2O_2+H]^+$ = 423.2073 (calc.), 423.2079 (found).

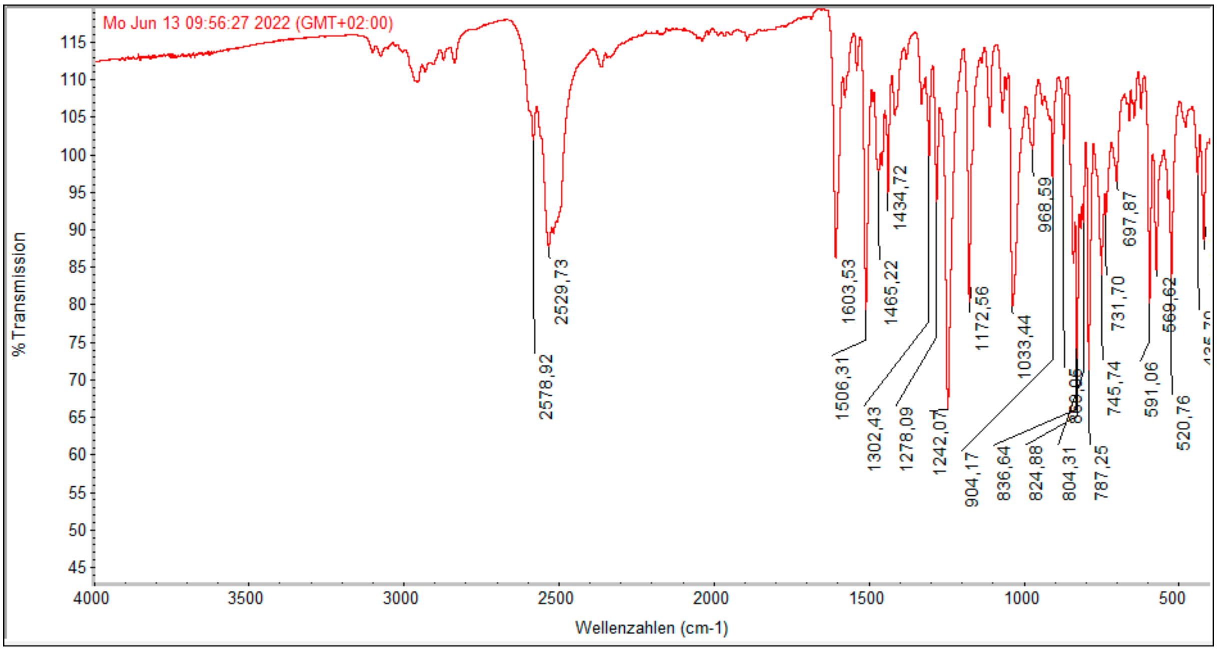

Figure S24. IR: 2961(w, C_{alk}-H), 2927 (w, C_{alk}-H), 2580-2500 (m, v (B-H)), 1603 (m, v(C=C)), 1506 (m, C_{arom}-H in-plane bending), 1465 (w, C_{arom}-H in-plane bending), 1278 (w), 1242 (s, C_{arom}-O), 1173 (m, C_{arom}-O) 1033 (m, C_{alk}-O), 850 (m, C_{arom}-H out-of-plane bending), 746 (m, C_{arom}-H out-of-plane bending) 591 (m), 521 (w).

2. X-ray crystallography

The data were collected on a Gemini diffractometer (Rigaku Oxford Diffraction) using Mo-Kα radiation and ω-scan rotation. Data reduction was performed with CrysAlisPro [1] including the program SCALE3 ABSPACK for empirical absorption correction. All structures were solved by dual space methods with SHELXT [2] and the refinement was performed with SHELXL [3]. Non-hydrogen atoms were refined with anisotropic displacement parameters. Hydrogen atoms for **5**, **6** and **7** were calculated on idealised positions using the riding model, whereas for **8** a difference-density Fourier map was used to locate all hydrogen atoms. Structure figures were generated with Diamond [4].

The structures of **5** and **6**, as well as of **7** and **8**, are isotypic. For **7** and **8**, the carborane carbon atoms could be localised with a bond length and displacement parameter analysis.

CCDC deposition numbers given in the Tables below contain the supplementary crystallographic data for this paper. These data can be obtained free of charge via https://summary.ccdc.cam.ac.uk/structure-summary-form (or from the Cambridge Crystallographic Data Centre, 12 Union Road, Cambridge CB2 1EZ, UK; fax: (+44)1223-336-033; or deposit@ccdc.cam.uk).

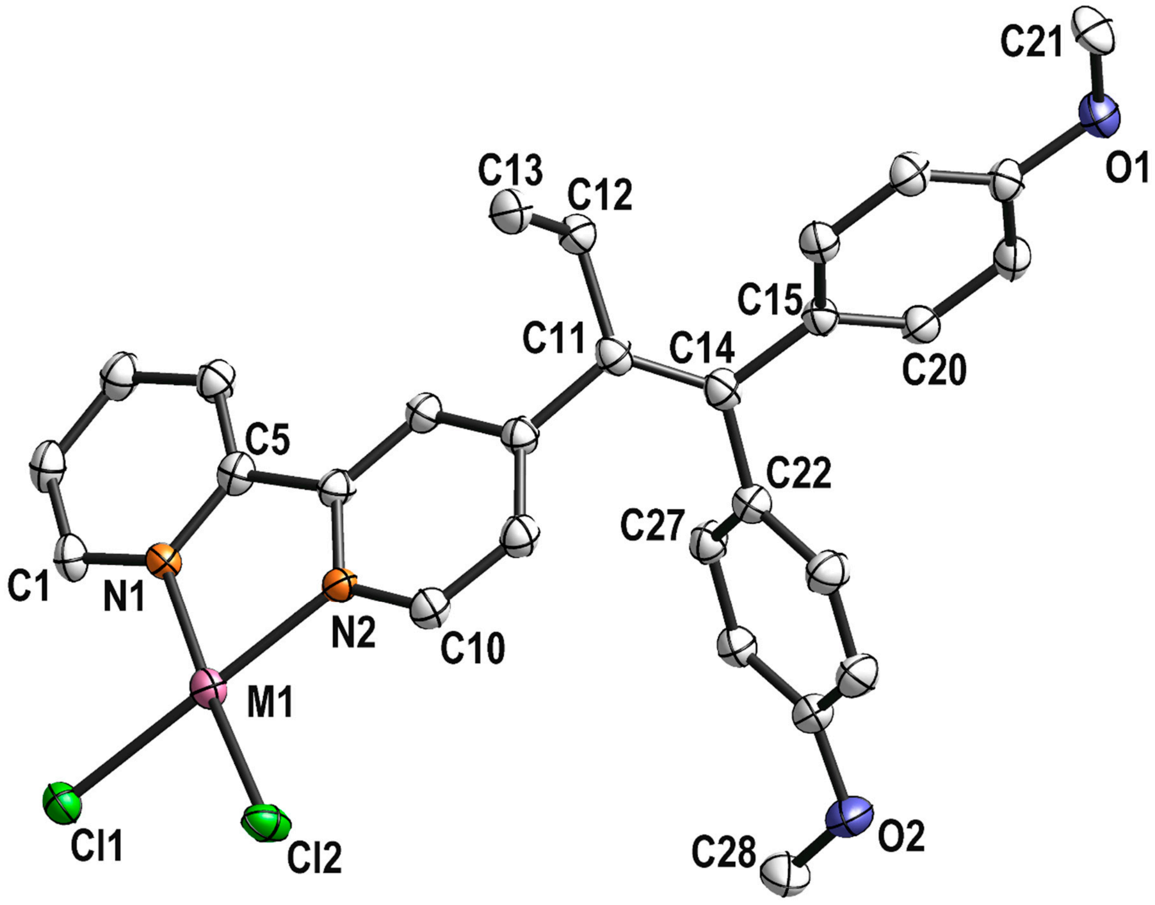

Figure S25. Molecular structures of **5** (M1 = Pt) and **6** (M1 = Pd) complexes in the solid state. Hydrogen atoms are omitted for clarity. Displacement ellipsoids are presented at 50% probability level. The calculated bond lengths and angles at PBE0 D3BJ/def2-TZVPP level of theory are in agreement with the crystallographic data. N1–Pt (2.010 calc., 2.019 exp.), N2–Pt (2.015 calc., 2.021 exp.), Cl1–Pt (2.296 calc., 2.305 exp.), Cl2–Pt (2.286 calc., 2.291 exp.); ∠N1–Pt–Cl1 (93.9° calc, 94.3° exp); ∠N2–Pt–Cl2 (95.1° calc, 94.8° exp); ∠Cl1–Pt–Cl2 (90.4° calc, 90.0° exp); ∠N1–Pt–N2 (80.6° calc, 81.1° exp). N1–Pd (2.010 calc., 2.019 exp.), N2–Pd (2.015 calc., 2.021 exp.), Cl1–Pd (2.296 calc., 2.305 exp.), Cl2–Pd (2.286 calc., 2.291 exp.); ∠N1–Pd–Cl1 (94.4° calc, 94.1° exp); ∠N2–Pd–Cl2 (94.2° calc, 94.3° exp); ∠Cl1–Pd–Cl2 (91.7° calc, 91.1° exp); ∠N1–Pd–N2 (79.7° calc, 80.6° exp).

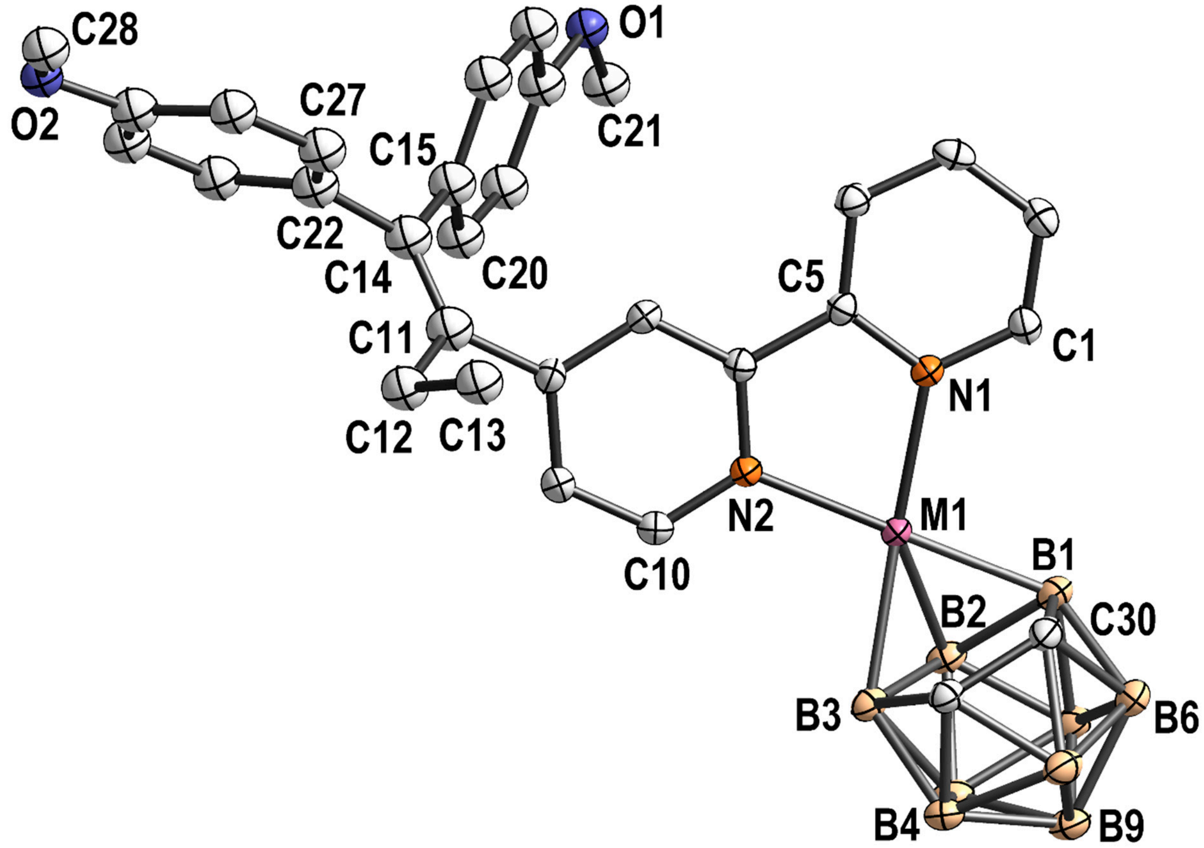

Figure S26. Molecular structure of **7** (M1 = Pt) and **8** (M1 = Pd) in the solid state. Hydrogen atoms are omitted for clarity. Displacement ellipsoids are presented at 50% probability level. The calculated bond lengths and angles at PBE0 D3BJ/def2-TZVPP level of theory are in agreement with the crystallographic data with the accuracy. N1−Pt (2.081 calc., 2.082 exp.), N2−Pt (2.074 calc., 2.072 exp.), B1−Pt (2.189 calc., 2.191 exp.), B2−Pt (2.172 calc., 2.183 exp.), B3−Pt (2.183 calc., 2.179 exp.); ∠B1−Pt−B3 (77.5° calc, 77.9° exp); ∠N1−Pt−N2 (77.1° calc, 77.1° exp); ∠N1−Pt−B1 (102.8° calc, 102.5° exp); ∠N2−Pt−B3 (102.6° calc, 102.5° exp), ∠B1−Pt−B2 (49.0° calc, 48.9° exp), ∠B2−Pt−B3 (49.2° calc, 49.0° exp). N1−Pt (2.081 calc., 2.082 exp.), N2−Pt (2.074 calc., 2.072 exp.), B1−Pt (2.189 calc., 2.191 exp.), B2−Pt (2.172 calc., 2.183 exp.), B3−Pt (2.183 calc., 2.179 exp.); ∠B1−Pt−B3 (77.5° calc, 77.9° exp); ∠N1−Pt−N2 (77.1° calc, 77.1° exp); ∠N1−Pt−B1 (102.8° calc, 102.5° exp); ∠N2−Pt−B3 (102.6° calc, 102.5° exp), ∠B1−Pt−B2 (49.0° calc, 48.9° exp), ∠B2−Pt−B3 (49.2° calc, 49.0° exp). N1−Pd (2.113 calc., 2.110 exp.), N2−Pd (2.105 calc., 2.098 exp.), B1−Pd (2.172 calc., 2.178 exp.), B2−Pd (2.238 calc., 2.270 exp.), B3−Pd (2.167 calc., 2.190 exp.); ∠B1−Pd−B3 (76.7° calc, 77.2° exp); ∠N1−Pd−N2 (77.5° calc, 77.8° exp); ∠N1−Pd−B1 (103.0° calc, 103.0° exp); ∠N2−Pd−B3 (102.8° calc, 102.9° exp), ∠B1−Pd−B2 (47.6° calc, 46.9° exp), ∠B2−Pd−B3 (47.8° calc, 47.4° exp).

Table S1. Summary of the structure refinement data of complexes **5** and **6**.

**Empirical formula**	$C_{28}H_{26}Cl_2N_2O_2Pt$ (**5**)	$C_{28}H_{26}Cl_2N_2O_2Pd$ (**6**)
**Formula weight**	688.50	599.81
**Temperature**	130(2) K	130(2) K
**Wavelength**	71.073 pm	71.073 pm
**Crystal system**	Monoclinic	Monoclinic
**Space group**	$P2_1/c$	$P2_1/c$
**Unit cell dimensions**	a = 1102.57(2) pm	1105.69(3) pm
	b = 1142.19(2) pm	1140.40(4) pm
	c = 2005.24(4) pm	1996.46(8) pm
	α = 90°	90°
	β = 102.364(2)°	102.343(3)°
	γ = 90°	90°
**Volume**	2.46672(8) nm^3	2.4592(2) nm^3
**Z**	4	4
**Density (calculated)**	1.854 mg m^{-3}	1.620 mg m^{-3}
**Absorption coefficient**	5.935 mm^{-1}	1.002 mm^{-1}
***F*(000)**	1344	1216
**Crystal size**	0.40 x 0.25 x 0.10 mm^3	0.15 x 0.03 x 0.02 mm^3
**Theta range for data collection**	1.891 to 32.515°	1.885 to 28.193°
**Index ranges**	$-16 \leq h \leq 15$, $-17 \leq k \leq 16$, $-29 \leq l \leq 29$	$-13 \leq h \leq 10$, $-13 \leq k \leq 14$, $-24 \leq l \leq 24$
**Reflections collected**	32170	17624
**Independent reflections**	8303 [R(int) = 0.0365]	5257 [R(int) = 0.0806]
**Completeness (theta)**	100.0 % (30.51°)	100.0 % (25.35°)
**Absorption correction**	Semi-empirical from equivalents	Semi-empirical from equivalents
**Max. and min. transmission**	1.00000 and 0.49996	1.00000 and 0.86051
**Refinement method**	Full-matrix least-squares on F^2	Full-matrix least-squares on F^2
**Data / restraints / parameters**	8303 / 0 / 319	5257 / 0 / 319
**Goodness-of-fit on F^2**	1.190	1.017
**Final R indices [I>2sigma(I)]**	R1 = 0.0391, wR2 = 0.0712	R1 = 0.0495, wR2 = 0.0862
**R indices (all data)**	R1 = 0.0496, wR2 = 0.0753	R1 = 0.0998, wR2 = 0.1035
**Residual electron density**	2.288 and −1.766 e·$Å^{-3}$	0.727 and −0.778 e·$Å^{-3}$
**CCDC Number**	2233556	2233557

Table S2. Summary of the structure refinement data of complexes **7** and **8**.

Empirical formula	$C_{30}H_{37}B_9N_2O_2Pt$ (**7**)	$C_{30}H_{37}B_9N_2O_2Pd$ (**8**)
**Formula weight**	749.99	661.30
**Temperature**	130(2) K	130(2) K
**Wavelength**	71.073 pm	71.073 pm
**Crystal system**	Monoclinic	Monoclinic
**Space group**	$P2_1/c$	$P2_1/c$
**Unit cell dimensions**	a = 1516.72(2) pm	1517.09(1) pm
	b = 1122.53(1) pm	1126.62(1) pm
	c = 1908.55(2) pm	1906.74(2) pm
	α = 90°	90°
	β = 108.172(1)°	108.367(1)°
	γ = 90°	90°
**Volume**	3.08736(6) nm^3	3.09295(5) nm^3
**Z**	4	4
**Density (calculated)**	1.614 mg/m^3	1.420 mg/m^3
**Absorption coefficient**	4.578 mm^{-1}	0.633 mm^{-1}
***F*(000)**	1480	1352
**Crystal size**	0.25 x 0.10 x 0.03 mm^3	0.40 x 0.30 x 0.15 mm^3
**Theta range for data collection**	2.134 to 32.609°	2.129 to 34.785°
**Index ranges**	$-22 \le h \le 22$, $-16 \le k \le 16$, $-28 \le l \le 28$	$-24 \le h \le 24$, $-18 \le k \le 17$, $-30 \le l \le 30$
**Reflections collected**	58096	88279
**Independent reflections**	10663 [R(int) = 0.0467]	12812 [R(int) = 0.0342]
**Completeness (theta)**	100.0 % (30.51°)	100.0 % (33.14°)
**Absorption correction**	Semi-empirical from equivalents	Semi-empirical from equivalents
**Max. and min. transmission**	1.00000 and 0.66474	1.00000 and 0.98289
**Refinement method**	Full-matrix least-squares on F^2	Full-matrix least-squares on F^2
**Data / restraints / parameters**	10663 / 0 / 400	12812 / 0 / 545
**Goodness-of-fit on F^2**	1.022	1.049
**Final R indices [I>2sigma(I)]**	R1 = 0.0292, wR2 = 0.0592	R1 = 0.0269, wR2 = 0.0610
**R indices (all data)**	R1 = 0.0397, wR2 = 0.0633	R1 = 0.0332, wR2 = 0.0640
**Residual electron density**	1.382 and 1.740 e·Å^{-3}	1.079 and −0.711 e·Å^{-3}
**CCDC Number**	2233558	2233559

3. NMR study

The comparison of the chemical shifts in ^1H NMR spectra of the ligand **4** and the resulting complexes **5** and **6** after complexation with the respective metal complex precursors evidenced the formation of the complexes. The signals 1 and 10 of compounds **5** and **6** are the most affected ones. They are shifted downfield. When the chloride ligands are exchanged with *nido*-carborate the signals of the ligand are shifted upfield. The coordination of the *nido*-carborate dianion results in the formation of two isomers for **7** and **8** which are not distinguishable with NMR spectroscopy.

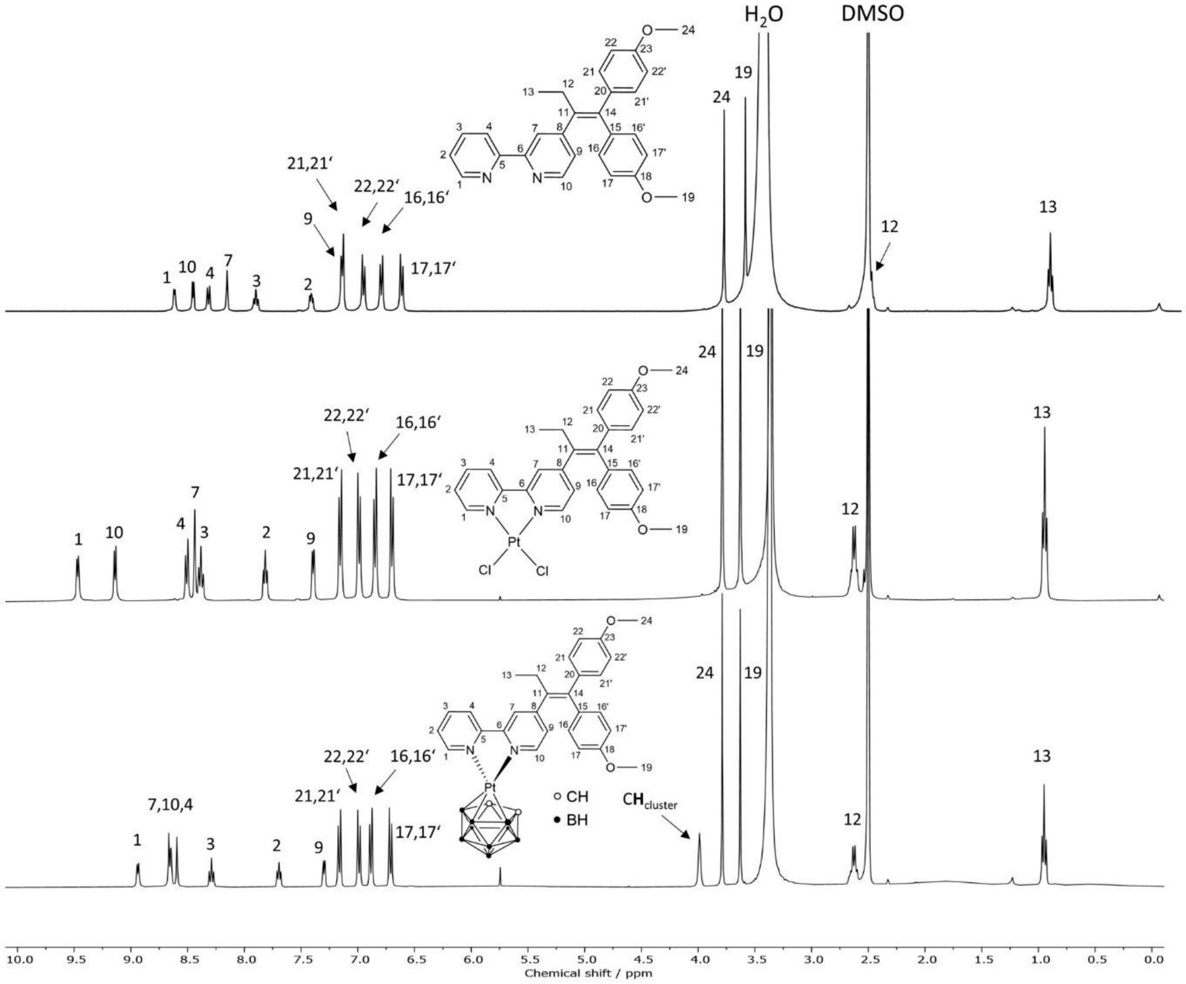

Figure S27. ^1H NMR spectrum of ligand **4** (top), platinum dichloride complex **5** (middle) and platinacarborane complex **7** (bottom) in DMSO-d_6. The hydrogen atoms 1, 2, 3, 4, 7, 9 and 10 in **5** are shifted downfield compared to the ligand **4**. On incorporation of *nido*-carborate, the signals 1, 2, 9 and 10 in **7** are shifted upfield and 7 is shifted downfield compared to **5**. Only small shifts of protons 12 in **5** and **7** are observed compared to ligand **4**.

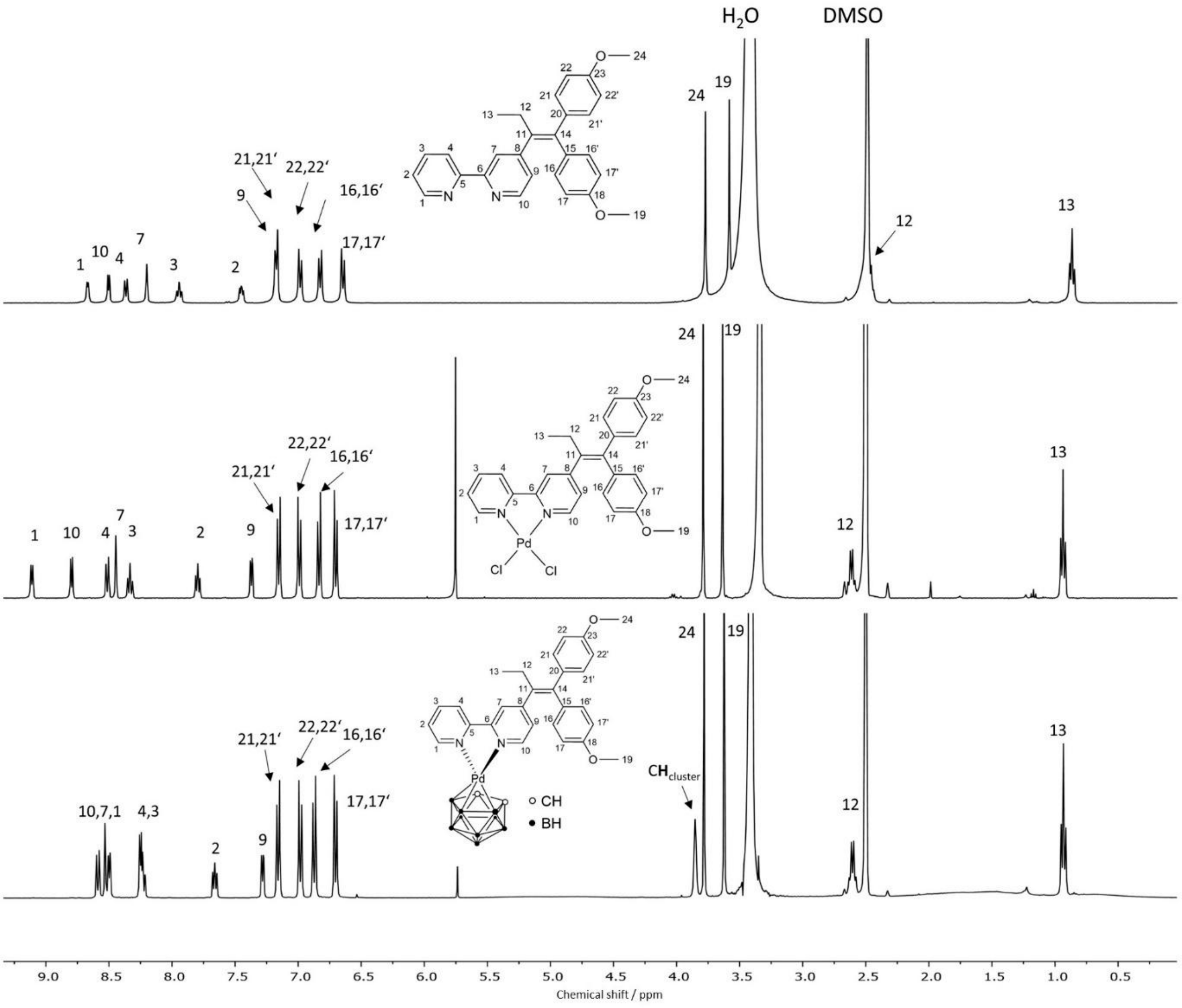

Figure S28. ${}^{1}H$ NMR spectra of ligand **4** (top), palladium dichloride complex **6** (middle) and palladacarborane complex **8** (bottom) in DMSO-d_6. The hydrogen atoms 1, 2, 3, 4, 7, 9 and 10 in **6** are shifted downfield compared to the ligand **4**. On incorporation of *nido*-carborate the signals 1, 2, 3, 4 and 10 in **8** are shifted upfield and 7 is shifted downfield compared to **6**. Only small shifts of protons 12 in **6** and **8** are observed compared to ligand **4**.

4. Stability

For the *in vitro* tests, stock solutions of the compounds in DMSO were prepared and stored at +4 °C. To assure the stability of the compounds, ${}^{1}H$ NMR spectra were recorded in water-containing DMSO-d_6 in air. The ligand **4** is stable in DMSO solution for one year, as was already shown by Schwarze et al. [5]. Complexes **5**–**8** were analysed over 30 days exposed to air using ${}^{1}H$ NMR spectroscopy; additionally, ${}^{11}B\{{}^{1}H\}$ NMR spectra were recorded for **7** and **8**. The stability tests revealed that complexes **5** and **6** can be stored for at least 30 days without decomposition. Platinacarborane complex **7** exhibited minor changes related to aggregation of this compound after 14 days in DMSO. The palladacarborane complex **8** when stored for up to 3 days in DMSO solution demonstrated minor dissociation with the release of $C_2B_9H_{11}^{2-}$.

4.1. Complex ***5***

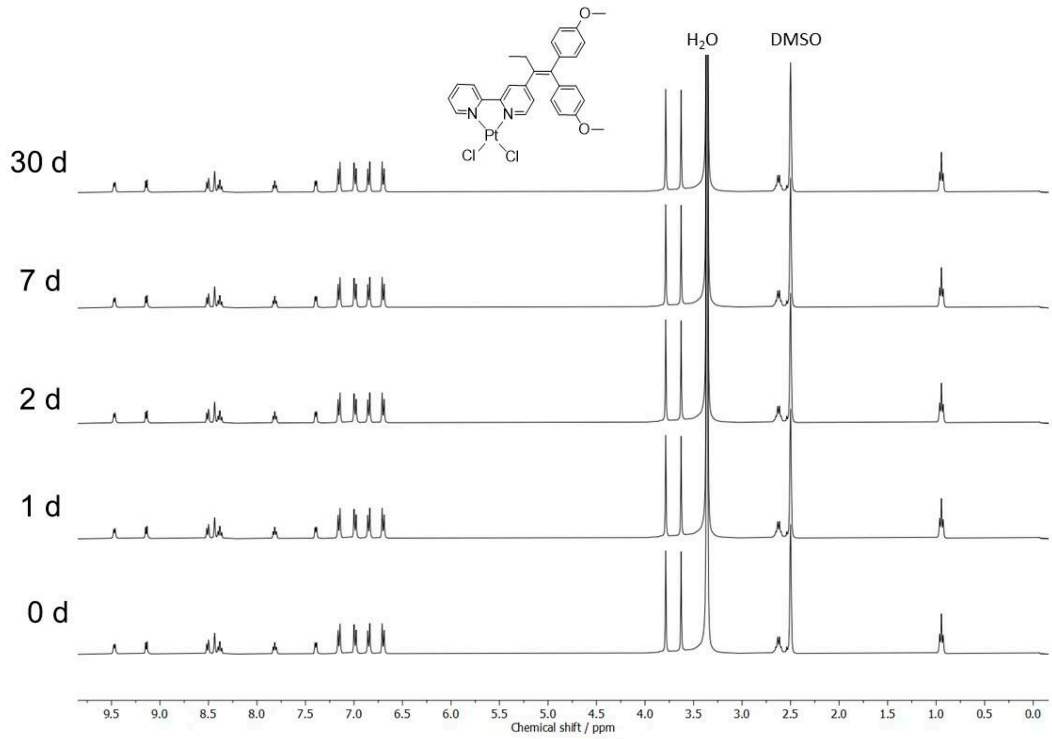

Figure S29. ^{1}H NMR spectra of **5** in DMSO-d6 recorded over a month at rt, but stored between the measurements at +4 °C. No changes can be observed.

4.2. Complex ***6***

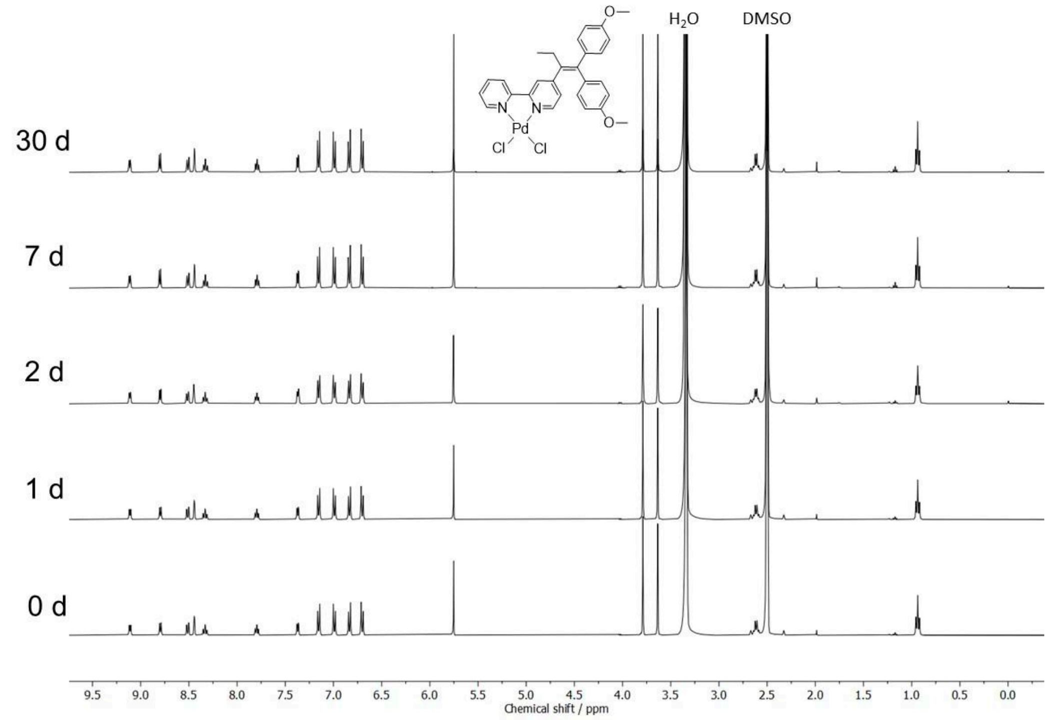

Figure S30. ^{1}H NMR spectra of **6** in DMSO-d6 recorded over a month at rt, but stored between the measurements at +4 °C. No changes can be observed.

*4.3. Complex **7***

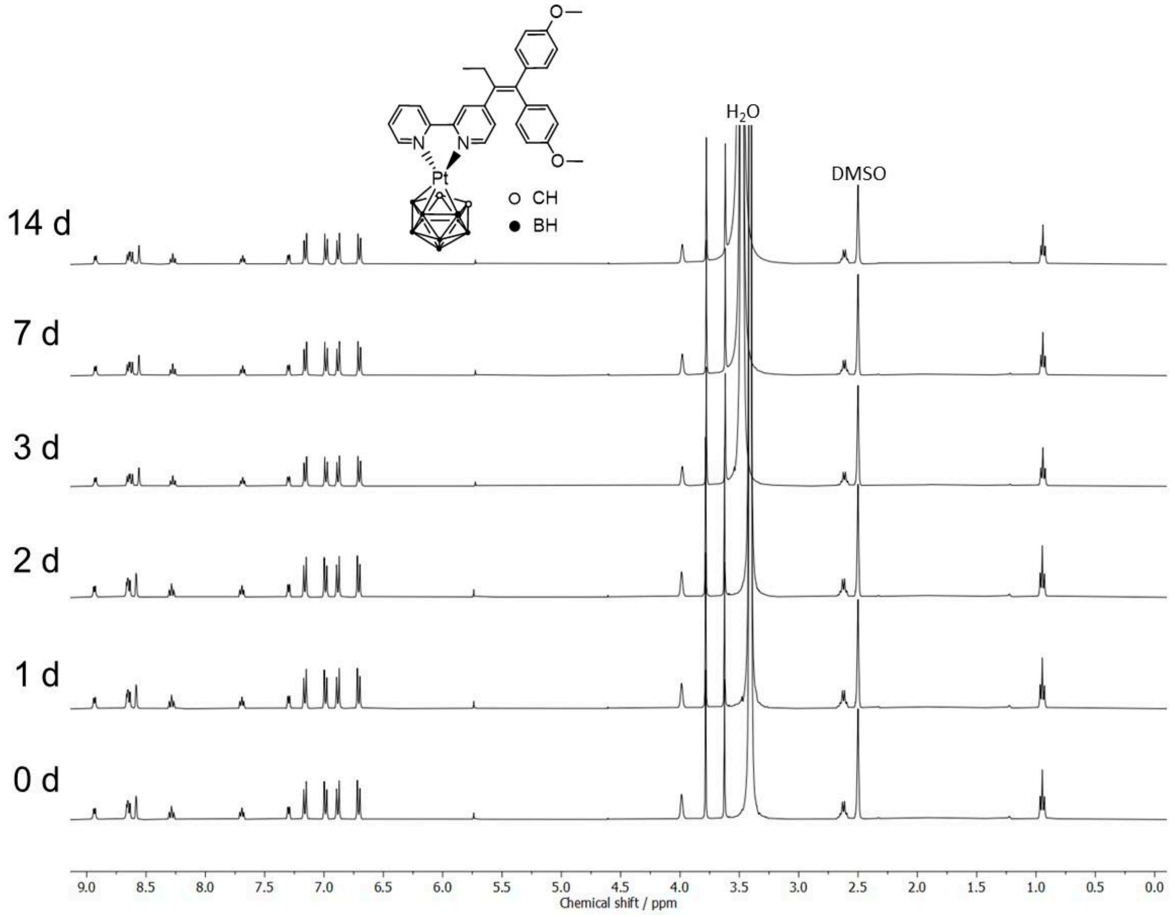

Figure S31. ^{1}H NMR spectra of **7** in DMSO-d_6 over 14 days at rt, but stored between the measurements at +4 °C. No changes can be observed.

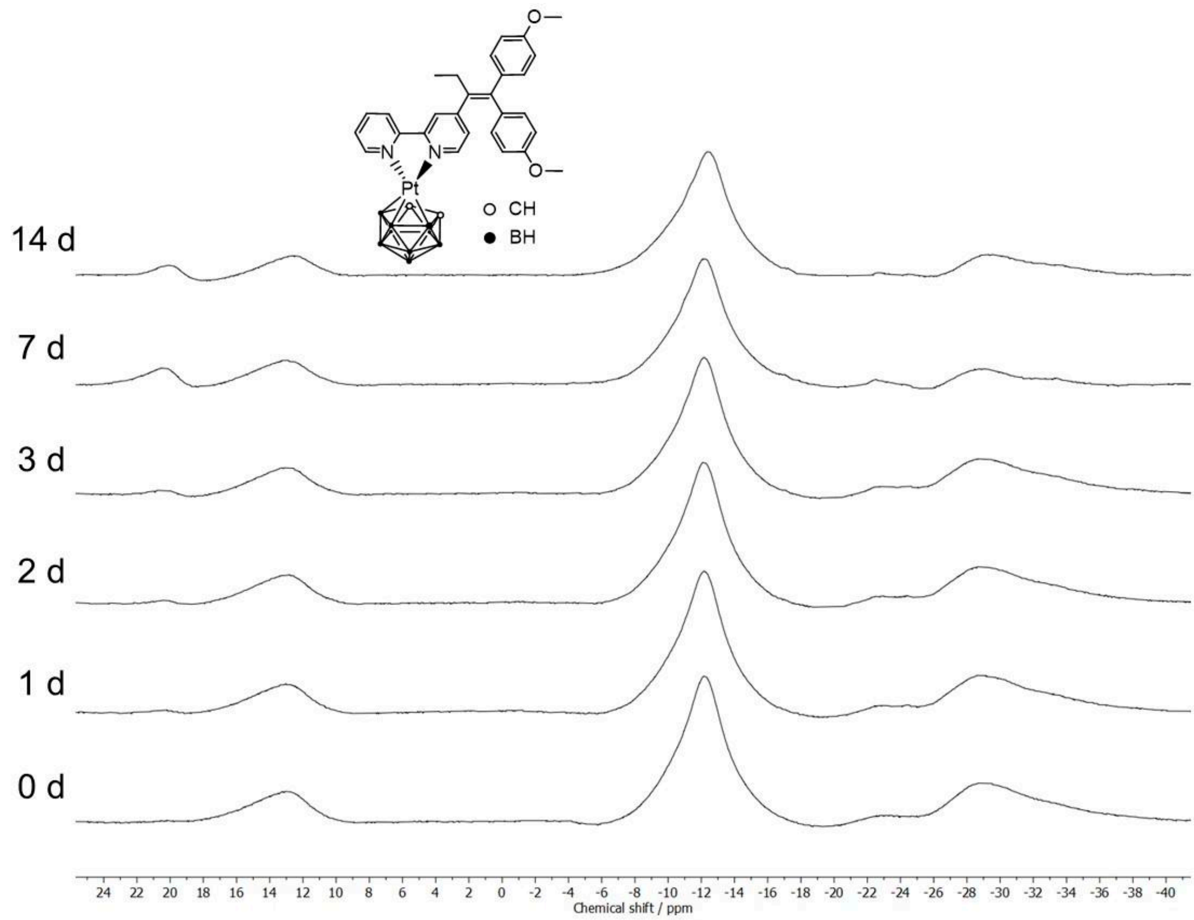

Figure S32. $^{11}B\{^{1}H\}$ NMR of **7** in DMSO-d_6 over 14 days at rt, but stored between the measurements at +4 °C. Minor changes are observed after 7 days, most likely related to self-assembly.

*4.4. Complex **8***

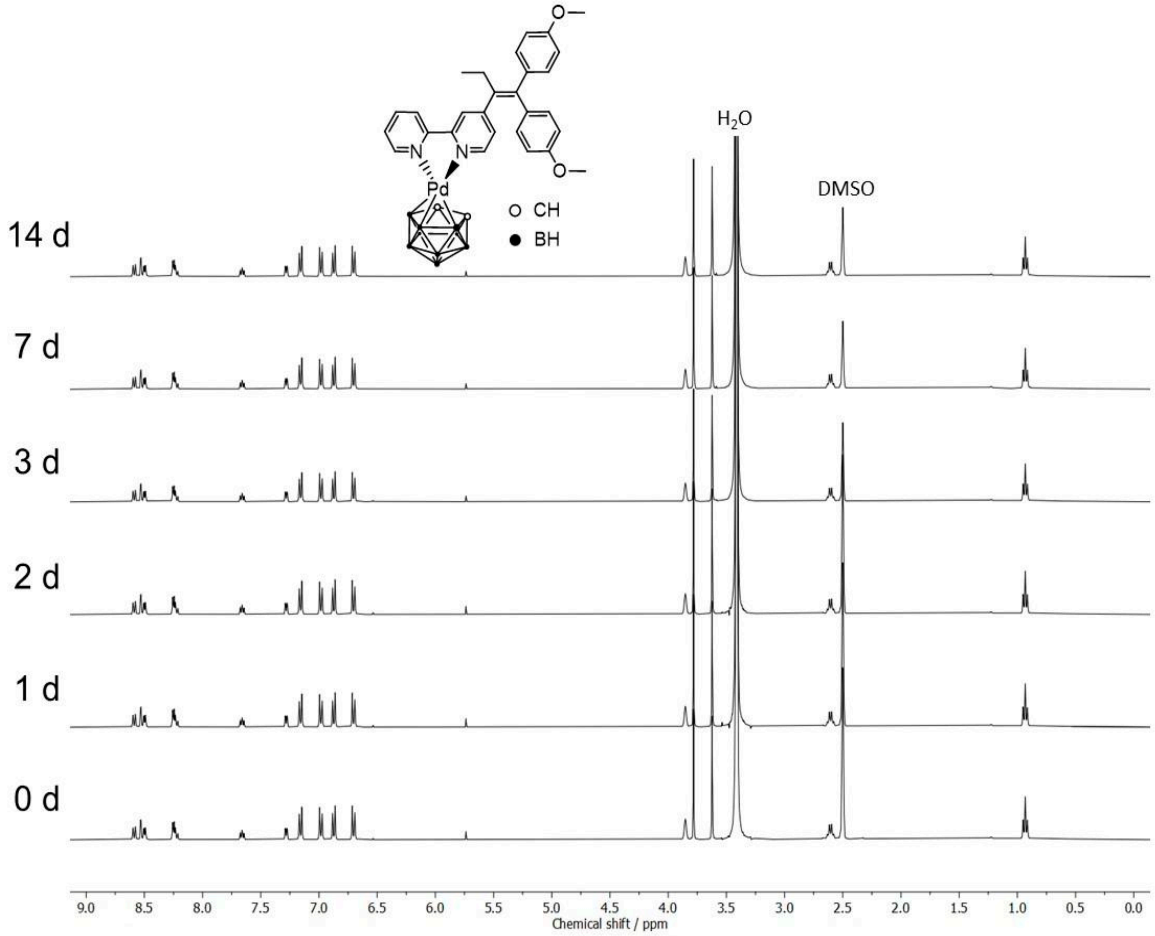

Figure S33. ^1H NMR spectra of **8** in DMSO-d_6 over 14 days at rt, but stored between the measurements at +4 °C. No changes can be observed.

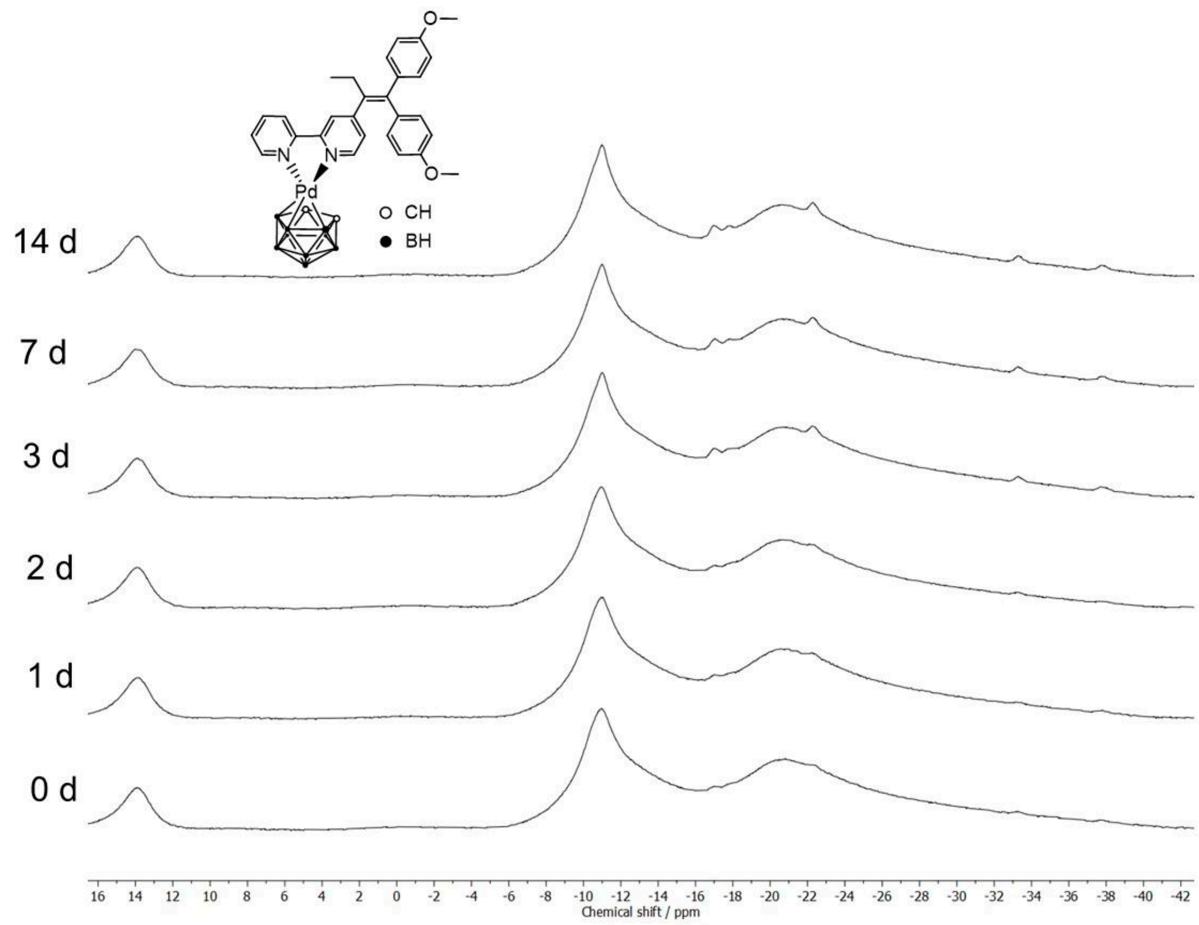

Figure S34. $^{11}B\{^{1}H\}$ NMR spectra of **8** in DMSO-d_6 over 14 days. Minor changes are observed after 3 days revealed by characteristic signals for *nido*-carborate.

5. Alternative synthetic strategies

One strategy to prepare complexes **7** and **8** was presented in the manuscript (see Scheme 1, B), which gave the best results. However, there are alternative routes, namely via the platinacarborane precursors, [3-(1′,2′:5′,6′-η^4-COD)-*closo*-3,1,2-$PtC_2B_9H_{11}$] (COD = 1,5-cyclooctadiene) (**12**) or [NEt_4][3-(η^3-C_3H_5)-*closo*-3,1,2-$PtC_2B_9H_{11}$] (**13**) [6], or palladacarborane [NEt_4][3-(η^3-C_3H_5)-*closo*-3,1,2-$PdC_2B_9H_{11}$] (**14**) [7].

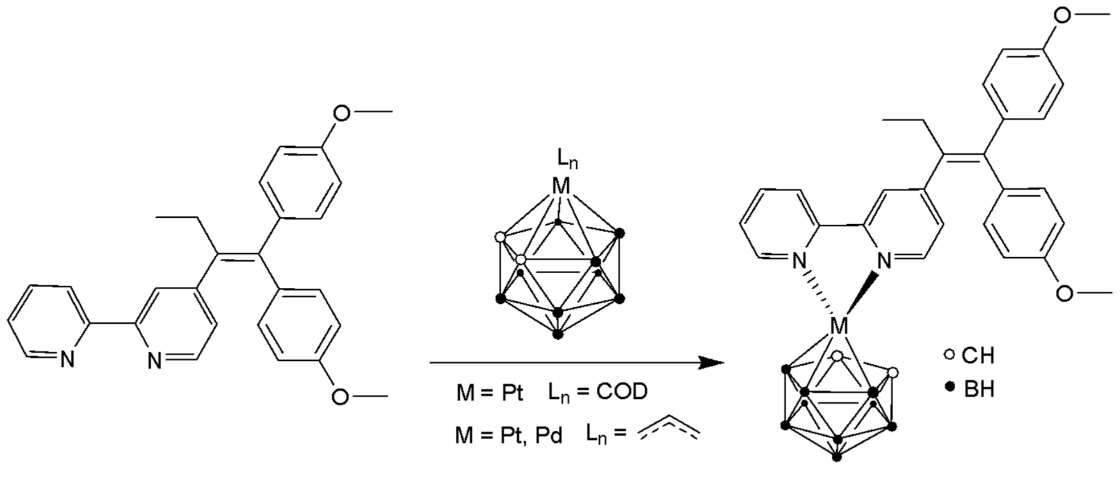

(**4**) M = Pt (**7**) or Pd (**8**)

Scheme S1. Alternative synthetic route towards **7** and **8**. Replacement of L_n in metallacarborane precursors of Pt with COD and Pt and Pd with allyl ligand (L_n) in ligand **4**.

Complex **12** was synthesised according to a modified procedure of Warren and Hawthorne [7] by reacting [$PtCl_2$(COD)] with $Na_2C_2B_9H_{11}$ at −80 °C in THF. In a second step, the COD ligand is supposed to be activated and replaced by ligand **4**. However, attempts to exchange the COD ligand with **4** under a variety of conditions were not successful (see Table S3). To better understand the bonding situation in complex **12**, we have carried out DFT calculation to identify the bonding interactions/strengths between platinum, COD and *nido*-carborate (Table S4). Our results suggest stronger interactions of Pt^{2+} with the COD ligand than with the *nido*-carborate cluster, which may explain the difficulties of the COD replacement. Although one example of COD replacement in the complexes [3-(1′,2′:5′,6′-η^4-COD)-*closo*-1,2-Me_2-3,1,2-$MC_2B_9H_9$] (M = Pt, Pd) by CO under CO pressure was reported [6,8], there are no examples of direct COD replacement with other stronger ligands. As the carbonyl complexes of platina- and palladacarboranes are even more stable, they were not considered as starting materials. The COD complex [3-(1′,2′:5′,6′-η^4-COD)-*closo*-3,1,2-$PdC_2B_9H_{11}$] was not described in the literature; however, its dimethyl-substituted derivative is known [8]. As a similar behaviour as observed for the corresponding platinacarborane complex can be expected, the palladium analogue was not prepared.

Suitable starting materials for the reaction in Scheme S1 are the platinum(II) and palladium(II) carborane allyl complexes [NEt_4][3-(η^3-C_3H_5)-*closo*-3,1,2-PtC_2B_9] and [NEt_4][3-(η^3-C_3H_5)-*closo*-3,1,2-$PdC_2B_9H_{11}$], which could be prepared according to a modified literature procedure for the methyl-substituted derivative [NEt_4][3-(η^3-C_3H_5)-*closo*-1,2-Me_2-3,1,2-$MC_2B_9H_9$], where M = Pt and Pd [8]. The protonation of the allyl ligand by $HBF_4{\cdot}Et_2O$ in THF followed by incorporation of the ligand **4** led to complexes **7** and **8**. Unfortunately, the yields

of the final step were very low. Considering the multiple synthetic steps for the starting materials **13** and **14**, this approach seemed inefficient.

Table S3. Summary of attempts to prepare metallacarborane complexes **7** and **8** starting from precursor complexes **12**, **13** or **14** and **4**. The reactions were carried out in inert atmosphere using Schlenk technique. The solvents used in the reactions were dried according to the literature [9]. 1 Eq. of ligand **4** was reacted with 1 eq. of **12**, **13** or **14**.

Starting material	Solvent	Conditions	Changes
**12** and **4**	THF	RT (12 h)	No changes, only starting materials were observed via NMR spectroscopy
	THF	Refluxing (12 h)	No changes, only starting materials were observed via NMR spectroscopy
	DMF	Refluxing (3 h)	Pink, unreacted ligand **4**, decomposition of **12**: black particles (probably of Pt^0), release of *nido*-carborane and boric acid
	Benzene	Refluxing (12 h)	Blue, unreacted ligand **4**, decomposition of **12**: release of *nido*-carborane
	Toluene	Refluxing (5 h)	Purple, unreacted ligand **4**, decomposition of **12**: formation of black particles and release of *nido*-carborane
	Ether	RT (12h)	No changes, only starting materials were observed via NMR spectroscopy
	THF	Refluxing with Zn powder (12 h)	No changes, only starting materials were observed via NMR spectroscopy
	DCM	LED lamp, RT (12 h)	Colour changed slightly, **12** decomposed slightly, release of *nido*-carborane
**13** and **4**	THF	$HBF_4{\cdot}Et_2O$, -80 °C	Colour changed from brown to deep brown, yield of **7** up to 5%
**14** and **4**	THF	$HBF_4{\cdot}Et_2O$, -80 °C	Colour changed from brown to orange-brown, yield of **8** up to 5%

6. Computational Chemistry

6.1. Geometry optimisation

Before docking and further studies with QTAIM, the structures of compounds **4–8** were fully optimised. The geometry optimisation was done based on X-ray crystal structures using density functional theory (DFT) [10] and performed with the ORCA 4.2 package [11]. The functional PBE0 has been chosen based on the results of benchmark studies for a set of the transition metal complexes [12,13]. In order to improve the stability of the optimised geometry Becke-Johnson dispersion correction of third order (D3BJ) [14] were included in the functional. We applied the density fitting technique resolution-of-identity approximation (RI-J) [15] and chain-of-sphere approximation (COSX) [15] in the geometry optimisation to speed up the calculations. The polarised basis set def2-TZVPP [16] for all non-transition metals and with the additional zeroth order regular approximation (ZORA) [17] for the transition metals was applied for better convergence of the energy. The local minimum of the optimised geometry was verified with numerical frequencies analysis, where no imaginary frequencies were observed.

6.2. UV-vis spectroscopy and transitions

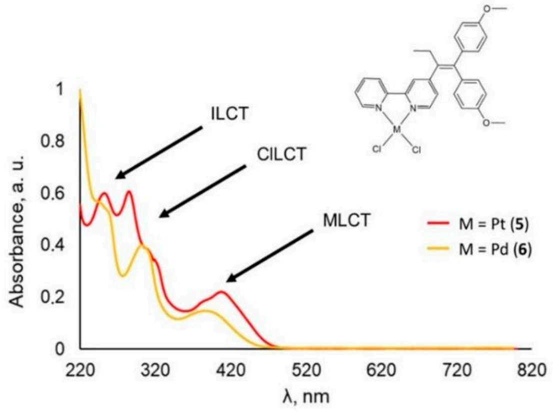

Figure S35. UV-vis spectra of compounds **5** and **6** recorded in acetonitrile.

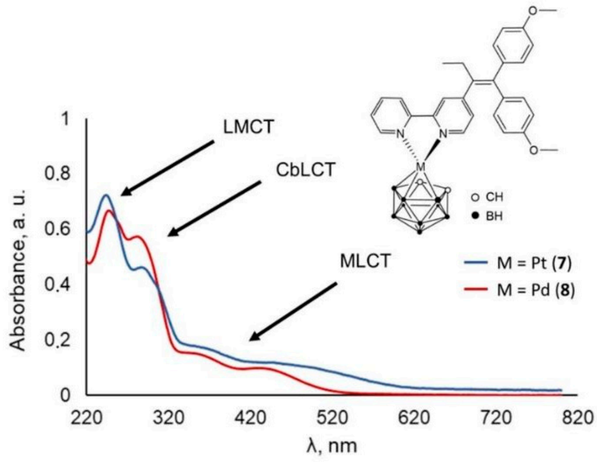

Figure S36. UV-vis spectra of compounds **7** and **8** recorded in acetonitrile.

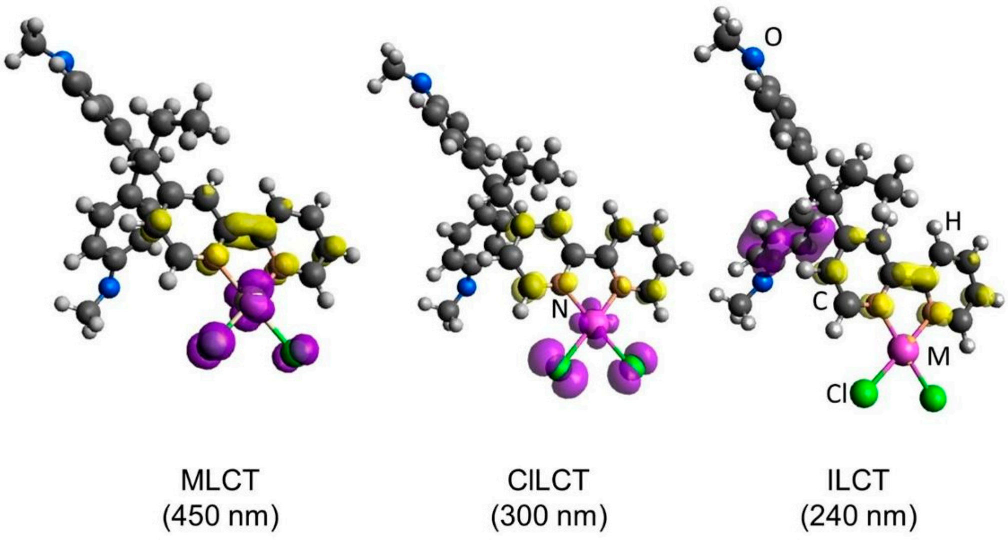

Figure S37. Difference densities of **5** and **6** visualising the charge transfer at certain wavelengths proceeding from purple to yellow iso-surfaces (L = ligand **4**, iso-value = 0.004). MLCT = metal-to-ligand **4** charge transfer, ClLCT = chloride-to-ligand **4** charge transfer, ILCT = intraligand **4** charge transfer.

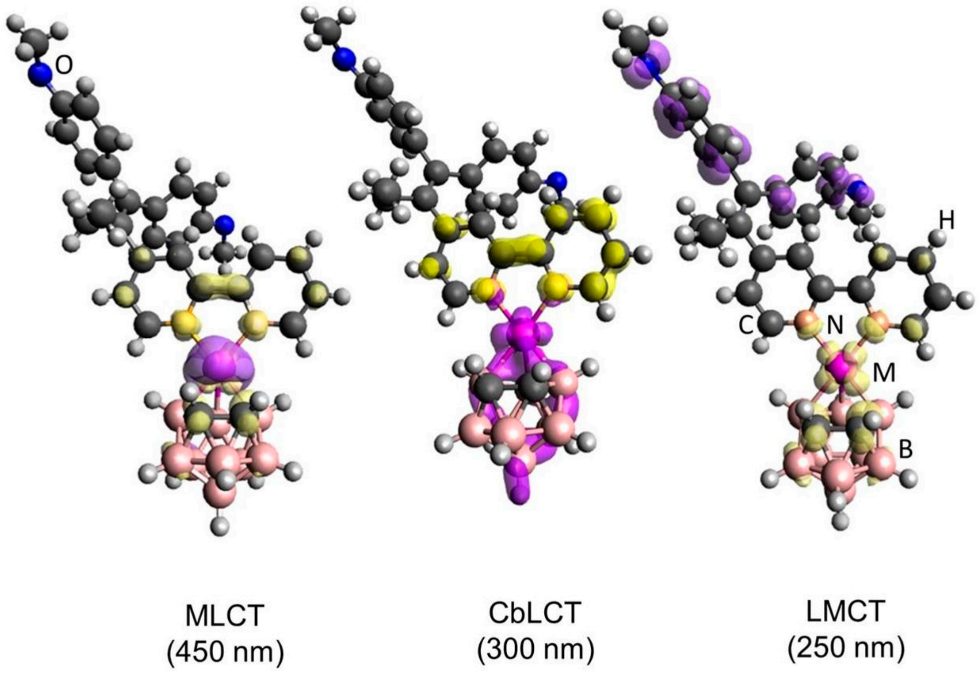

Figure S38. Difference densities of **7** and **8** visualising the charge transfer at certain wavelengths proceeding from purple to yellow iso-surfaces (L = ligand **4**, Cb = $[C_2B_9H_{11}]^{2-}$, iso-value = 0.004). MLCT = metal-to-ligand **4** charge transfer, CbLCT = $[C_2B_9H_{11}]^{2-}$-to-ligand **4** charge transfer, LMCT = ligand **4**-to-metal charge transfer.

6.3. QTAIM: bonding interactions

In order to estimate the bonding interactions in the transition metal complexes Quantum Theory of Atoms in Molecules (QTAIM) [18] was applied. According to this theory, the distribution of the electron density of the molecular system is structured in critical points (CPs) [18, 19], which fully characterise this system. The first ($\nabla \rho(r)$, gradient) and the second derivatives ($H\rho(r)$, Hessian matrix (1)) [18, 19] of the electron density ρ(**r**) reveal four types of CPs, which are the maxima, minima or saddle points of the electron density (ED).

$$H\rho(r) = \begin{bmatrix} \frac{\partial^2 \rho}{\partial x^2} & \frac{\partial^2 \rho}{\partial x \partial y} & \frac{\partial^2 \rho}{\partial x \partial z} \\ \frac{\partial^2 \rho}{\partial y \partial x} & \frac{\partial^2 \rho}{\partial y^2} & \frac{\partial^2 \rho}{\partial y \partial z} \\ \frac{\partial^2 \rho}{\partial z \partial x} & \frac{\partial^2 \rho}{\partial z \partial y} & \frac{\partial^2 \rho}{\partial z^2} \end{bmatrix} \quad (1)$$

In the CP, the gradient of the ED is equal to zero. When the molecule or molecular system is in the equilibrium state, matrix (1) can be diagonalised (all non-diagonal elements are zeros). The sum of the main diagonal elements of the matrix (1) is the Laplacian of ED (eq. (2)).

$$\nabla^2 \rho = \lambda_1 + \lambda_2 + \lambda_3 \quad (2)$$

According to the sum of the signs of λ, the four types of stable CPs can be distinguished: (3,−3): maximum, at the atomic nucleus the density reaches the maximum and all λ are negative; (3, −1): saddle point, the interatomic region between two atoms, where λ_1 and λ_2 are negative and λ_3 is positive (bonding CP or BCP); (3, +1): saddle point, the interatomic region between several atoms which form a ring where λ_1 and λ_2 are positive and λ_3 is negative; (3, +3): minimum, interatomic region where all λ are positive (molecular cage) [18,20].

In our description, we considered the topological parameters in the bonding critical points (BCPs, determine the interaction between two atoms) and in the ring critical points (RCPs). The RCPs indicate the interaction of several atoms, which form the ring. In order to classify these interactions, we considered such parameters as bond length, electron density (ED, ρ_{cp}), Laplacian of electron density ($\nabla^2\rho_{cp}$), density of potential energy (V_{cp}), density of the full electron energy (H_{cp}) and the ratio $|V_{cp}|/G_{cp}$ in CPs. Positive values of the Laplacian in BCPs indicate the depletion of the electron charge, while negative values demonstrate its concentration. The ratio of the potential and kinetic energy densities $|V_{cp}|/G_{cp}$ allows to classify the interactions into three groups: shared (covalent and polar-covalent with $|V_{cp}|/G_{cp} > 2$, $\nabla^2\rho_{cp} < 0$, $H_{cp} < 0$), transit (intermediate with $1 < |V_{cp}|/G_{cp} < 2$, $\nabla^2\rho_{cp} > 0$, $H_{cp} < 0$), and closed-shell interactions ($|V_{cp}|/G_{cp} < 1$, $\nabla^2\rho_{cp} > 0$, $H_{cp} > 0$) [19,21]. The greater the values of $|V_{cp}|$ compared to G_{cp} (and the greater the ratio $|V_{cp}|/G_{cp}$), the higher is the covalent character of the interaction. In general, in spite of the depletion of ED, at the BCPs of coordination bonds ($\nabla^2\rho_{cp} > 0$) these bonds are stabilised by the negative values of the full electron energy density ($H_{cp}<0$). The more negative H_{cp} the higher the stability of the interactions (Table S5).

Table S5. Topological properties of the electron density in the bond and ring critical points of complexes **5–8** and [3-(1′,2′:5′,6′-η^4-COD)-*closo*-3,1,2-$PtC_2B_9H_{11}$] (**12**)

Complex	Bond or ring	Bond length, Å	ρ_{cp}, a.u.	$\nabla^2\rho_{cp}$, a.u.	V_{cp}, a.u.	G_{cp}, a.u.	H_{cp}, a.u.	$\lvert V_{cp}\rvert/G_{cp}$
**5**	N1–Pt	2.019	0.131	0.465	−0.219	0.168	−0.051	1.3
	N2–Pt	2.021	0.133	0.462	−0.220	0.168	−0.052	1.3
	Cl1–Pt	2.305	0.108	0.210	−0.142	0.097	−0.045	1.5
	Cl2–Pt	2.291	0.110	0.210	−0.143	0.097	−0.046	1.5
	Pt–N1–C5–C6–N2	-	0.322	−0.969	−0.457	0.101	−0.356	4.3
	Pt–Cl1–H1–C1–N1	-	0.109	0.220	−0.144	0.100	−0.044	1.4
	Pt–Cl2–H10–C10–N2	-	0.014	0.061	−0.011	0.013	0.002	0.8
**6**	N1–Pd	2.017	0.113	0.452	−0.173	0.141	−0.032	1.2
	N2–Pd	2.020	0.111	0.448	−0.178	0.146	−0.032	1.2
	Cl1–Pd	2.276	0.098	0.236	−0.121	0.090	−0.031	1.5
	Cl2–Pd	2.286	0.099	0.235	−0.122	0.090	−0.032	1.5
	Pd–N1–C5–C6–N2	-	0.326	−0.991	−0.464	0.108	−0.356	4.3
	Pd–Cl1–H1–C1–N1	-	0.096	0.236	−0.122	0.090	−0.317	1.4
	Pd–Cl2–H10–C10–N2	-	0.013	0,057	−0.010	0.012	0.002	0.8
**7**	B4–Pt	2.191	0.099	−0.012	−0.088	0.043	−0.045	2.1
	B8–Pt	2.183	0.095	0.035	−0.095	0.052	−0.043	1.8
	B7–Pt	2.179	0.100	−0.014	−0.089	0.043	−0.046	2.1
	N1–Pt	2.077	0.112	0.398	−0.175	0.137	−0.038	1.3
	N2–Pt	2.082	0.114	0.401	−0.179	0.140	−0.039	1.3
	Pt–(B4, B7) (Pt–cage)	–	0.042	0.144	−0.042	0.039	−0.003	1.1
	Pt–N1–C5–C6–N2	–	0.121	−0.121	−0.108	0.039	−0.069	2.8
**8**	B4–Pd	2.178	0.089	0.031	−0.081	0.044	−0.037	1.8
	B8–Pd	2.270	0.078	0.109	−0.079	0.053	−0.026	1.5
	B7–Pd	2.190	0.090	0.029	−0.082	0.045	−0.037	1.8
	N1–Pd	2.111	0.096	0.400	−0.144	0.122	−0.022	1.2
	N2–Pd	2.098	0.094	0.395	−0.140	0.119	−0.021	1.2
	Pd–(B4, B7) (Pd–cage)	–	0.042	0.156	−0.044	0.041	−0.003	1.0
	Pd–N1–C5–C6–N2	–	0.120	−0.120	−0.107	0.038	−0.069	2.8
**12**	C1′–Pt	2.189	0.120	0.105	−0.141	0.083	−0.058	1.7
	C2′–Pt	2.201	0.101	0.142	−0.118	0.077	−0.041	1.5
	C5′–Pt	2.178	0.098	0.154	−0.115	0.077	−0.038	1.5
	C6′–Pt	2.135	0.120	0.108	−0.142	0.084	−0.058	1.7
	C1–Pt	2.337	0.093	0.179	−0.113	0.079	−0.034	1.4
	B4–Pt	2.235	0.082	0.052	−0.078	0.045	−0.033	1.7
	B8–Pt	2.274	0.099	−0.047	−0.091	0.040	−0.051	2.3
	B7–Pt	2.191	0.081	0.038	−0.073	0.041	−0.032	1.8

Short bond length, high ρ_{cp} values and lower H_{cp} of N–M compared to Cl–M evidence stronger bonding between the nitrogen atoms and the metal cations than between Cl and M. Despite slightly higher covalency of the Cl–M bond ($\lvert V_{cp}\rvert/G_{cp}$ of the N–M bond is insignificantly lower than this ratio of Cl–M), the parameters in RCP demonstrate that the ED concentrates in the M–N1–C5–C6–N2–M ring with great stabilising negative values of $\nabla^2\rho_{cp}$

and H_{cp}. The interaction between Pt^{2+} or Pd^{2+} and ligand **4** is stronger than between the metal cations and the chlorides. Chloride ligands in complexes **5** and **6** form H bonds and, thus, close the rings M−Cl−H−C−N−M. The topological features of the RCPs, however, demonstrate that the formed rings are less stable than those containing the bidentate 2,2′-bpy ligand.

Several interesting features were observed for BCPs when considering the pair atomic interactions between polyhedral cluster and metal cations. For instance, even though the distances between the carbon atoms of the *nido*-carborate and the metal cations are less than the sum of the Van der Waals radii, the bond paths $C_{cluster}$−M are not formed, while BCPs and bond paths exist between M and B atoms. However, in comparison to each other, B−Pt and B−Pd bonds show different character. For example, the platinum metal in compound **7** binds to B4 and B7 with a higher covalency compared to B8. This is evidenced by the negative values of the Laplacian, the lower stabilising H_{cp} and the ratio $|V_{bcp}|/G_{bcp}$ being slightly greater than 2. In palladacarborane complex **8**, B7−Pd and B4−Pd bonds are shorter and exhibit more covalent character than B8−Pd (lower $\nabla^2\rho_{cp}$ values, the total energy density is more negative and the ratio $|V_{cp}|/G_{cp}$ is closer to 2 in the BCPs for B7−Pd and B4−Pd than for B8−Pd). However, when we compare the binding nature of the sum of B−Pt and B−Pd bonds, we observe that the B−Pt bonds are of greater covalent character than the B−Pd bonds. The lower ρ_{cp} values and only positive $\nabla^2\rho_{cp}$ at the BCPs of B−Pd indicate the depletion of the electron charge for these bonds. In other terms, the bonding interactions between $[C_2B_9H_{11}]^{2-}$ and Pt^{2+} are stronger than between $[C_2B_9H_{11}]^{2-}$ and Pd^{2+}. This may reveal different biological properties of the compounds **7** and **8**, depending on the nature of the metal, when stability of these bonds is a key property.

The same analysis was performed for **12**. Although the bonding character of B−Pt exhibits equal or even higher extent of covalency, the ED values in the BCPs of B−Pt are lower than those observed for Pt−COD. The sum of H_{cp} for Pt−COD bonds are lower than for the (*nido*-carborate)−Pt bonds showing the stronger COD−Pt interaction. This could be a reason why we observed release of *nido*-carborate in some of the trials to replace the COD ligand.

The molecular graph (Figure S30) also demonstrates that one RCP in Pt−(B7,B8) ring is very close to the BCP of B8−Pt. Under certain conditions, these two points overlap with each other which leads to a merging of both the BCP and RCP. This situation is called catastrophe situation [22]. It also indicated the less stable interaction of Pt^{2+} with the *nido* cluster. This additionally explains the higher possibility to release $[C_2B_9H_{11}]^{2-}$ instead of COD (Table S3).

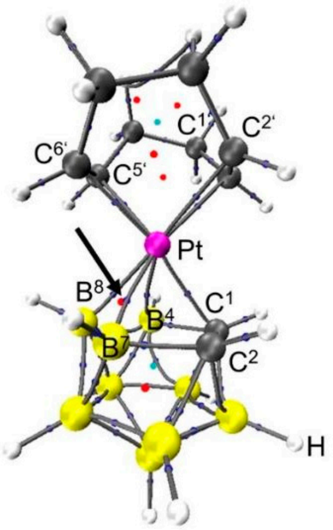

Figure S39. QTAIM analysis: molecular graph of [3-(1′,2′:5′,6′-η^4-COD)-*closo*-3,1,2-$PtC_2B_9H_{11}$] (**12**). The blue dots are BCPs, the red dots are RCPs and the grey lines connecting the nuclei and BCPs are the bond paths. The arrow shows the catastrophe, where the close position of the BCP and RCP may lead to the merge of the bond path.

6.4. Protein structure preparation

The crystal structure of the oestrogen receptor α was prepared for docking. The X-ray structure of the ERα is available from the protein data bank (PDB) under the code 3ERT [23] and contains the dimer with 4-hydroxytamoxifen as ligand in the ligand-binding pocket. One identical monomer was removed from the file and the other with the initial ligand was protonated using the Reduce program [24]. The force field CHARMM [25] was assigned in NAMD [26] for the atoms of the receptor, the ligand and water in the initial structure (3ERT). The molecular dynamic minimisation (Langevin dynamics) [27] with the ligand in the binding pocket of the receptor has been applied in order to avoid the contraction of the binding pocket. The dynamic was run for 500 ps with Langevin thermostat at 298 K. The integration time step is 2 fs/step and the size of the water cell is 70 x 64 x 82 $Å^3$ with the centre placed at 4.55, 2.82, 33.22 of x,y,z-coordinates. The rigid model was applied for the water molecules (TIP3P). For the long-range electrostatic interaction, the particle-meshed Edwald method (PME) was used [28]. The coordinates of the minimised energy structure have been taken and 4-hydroxytamixfen has been removed from the binding pocket for further docking.

6.5. Docking

The molecular docking was performed with the AutoDockTools4 software [29] using the Lamarckian Genetic Algorithm [30]. The force-field parameters for the transition elements (Pt and Pd) and boron atoms were added manually in the parameter file of the AutoDockTools4 library. The water molecules were eliminated and the non-polar hydrogen atoms were merged. The docking area was limited by the constructed grid box of the size 58 x 64 x 88 centred at 32.336, −1.613, 26.672 of x,y,z-coordinates (based on the position of the LBD). The following parameters were used in the docking: number of hybrid GA-LS runs: 500; population size: 150; maximum number of energy evaluations: 25,000,000, maximum number of top individuals to survive to next generation: 1; rate of gene mutation: 0.02; rate of crossover:

0.8; Mean of Cauchy distribution for gene mutation: 0.0; variance of Cauchy distribution for gene mutation: 1.0.

All the agonists such as the endogenous 17β-estradiol as well as some of the known synthetic antagonists such as ICI-164384 [31] exclusively bind to the C-terminal ligand-binding domain (LBD) [23]. Tamoxifen as a selective oestrogen receptor modulator (SERM) is an antagonist [23, 32]. Its metabolite 4-hydroxytamoxifen binds non-covalently to the LBD and induces the conformational change of helix 12, which is essential for activation function 2 (AF-2) activity [23]. In order to obtain information about the putative binding modes of compounds **2–9** to the ERα, docking simulations with **3–9** into the LBD of ERα (PDB code: 3ERT; [23]) were conducted using AutoDockTools4 [29]. The best ranked positions are shown in Figure S31 relative to the position of the metabolites 4,4′-dihydroxytamoxifen (**3**) and 4-hydroxytamoxifen (**2**).

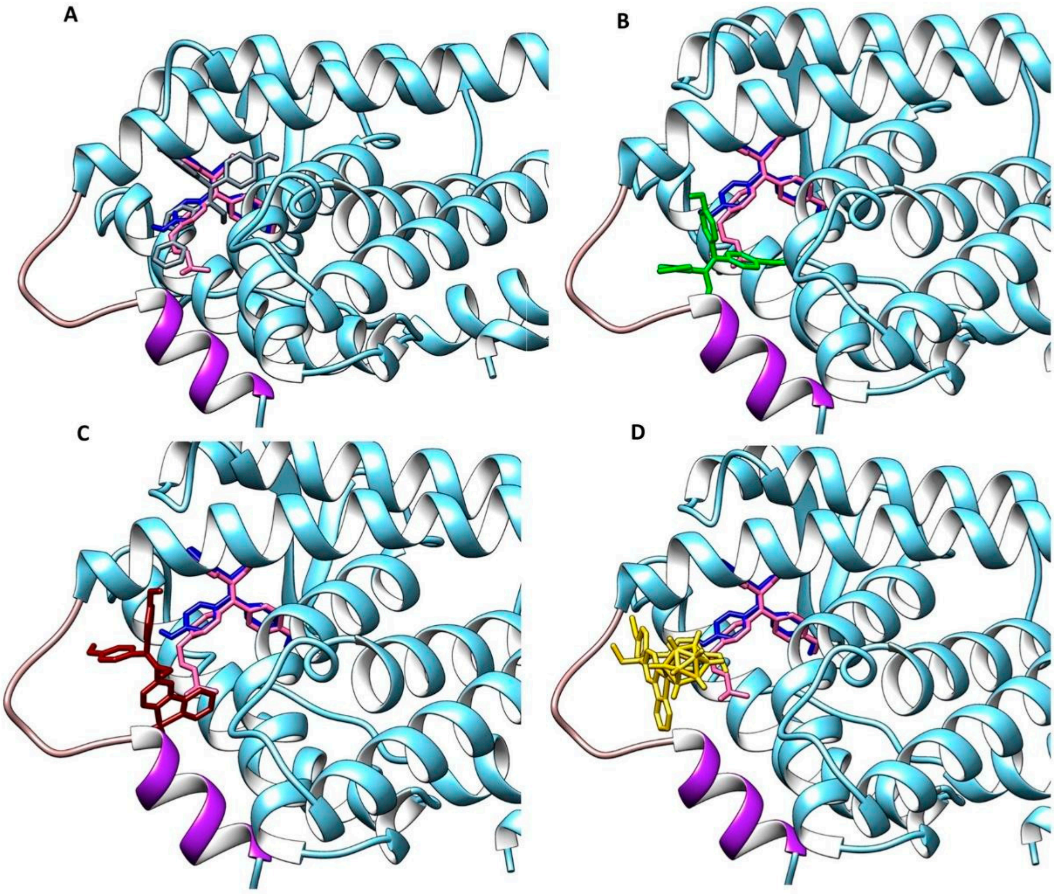

Figure S40. *In silico* investigation of the binding modes of compounds **2–9** based on docking. The highest ranked docked positions of compounds **9** (grey, **A**), **4** (green, **B**), **5** or **6** (magenta, **C**) and **7** or **8** (yellow, **D**) are shown compared to the position of the **2** (pink) and **3** (dark blue). Helix 12 is shown in purple, and the loop of the receptor is shown in light brown.

First, the binding energy of **3** was estimated by docking the molecule into the X-ray structure of the ERα (PDB code: 3ERT). We observed that the majority of conformations of **3** occupy a similar position as the original metabolite **2** with a scored binding energy value of

−10.73 kcal mol^{-1} (the binding energy is −10.80 kcal mol^{-1} for **2**, Figure S40). Both metabolites do not bind directly to helix 12 and probably induce the conformational changes of this helix indirectly [23]. Incorporation of 2,2′-bpy in the tamoxifen structure decreased the energy of the interaction with the LBD, with a binding energy of −8.44 kcal mol^{-1} (ligand **4**) and −8.76 kcal mol^{-1} (ligand **9**). However, the best ranked position of **9** suggests that this compound occupies the same positions as the metabolites **2** and **3**, while the predicted poses show that the best model position of **4** is located at the receptor loop, suggesting direct interaction with the loop and helix 12. Once the 2,2′-bpy unit was combined with the $PtCl_2$ or $PdCl_2$ moieties (**5** and **6**, respectively) the binding energies slightly improved (−9.08 and −8.99 kcal mol^{-1}, respectively). Further, the incorporation of the bulky carborane appears to have a destabilising impact on the binding energy of compound **7** (−8.14 kcal mol^{-1}). However, the palladium complex **8** showed comparable affinity and orientation, in comparison to compounds **5** and **6**, but with a higher binding energy of −9.71 kcal mol^{-1}.

7. Bioanalytical measurements

7.1. PI staining on chamber slides

MCF-7 cells were seeded overnight at a density of 1.5 × 10^3 cells/well followed by treatment with an IC_{50} dose of the compounds **4**–**6** for 60 h. After incubation, the cells were washed with phosphate-buffered saline (PBS) and fixed with 4% paraformaldehyde (PFA) for 15 min at rt. Thereafter, the cells were washed with PBS and stained with a solution of propidium iodide (PI) in a concentration of 50 μg mL^{-1} with 0.1 mM ethylenediaminetetraacetic acid (EDTA) at pH 8.0, 0.1% Triton X-100 and RNase (85 μg mL^{-1}) in PBS for 2 min. Afterwards, for covering the slides, fluorescent mounting medium was used. Finally, the slides were analysed with a Zeiss AxioObserver Z1 inverted fluorescence microscope (Carl Zeiss AG, Oberkochen, Germany) at 200x magnification.

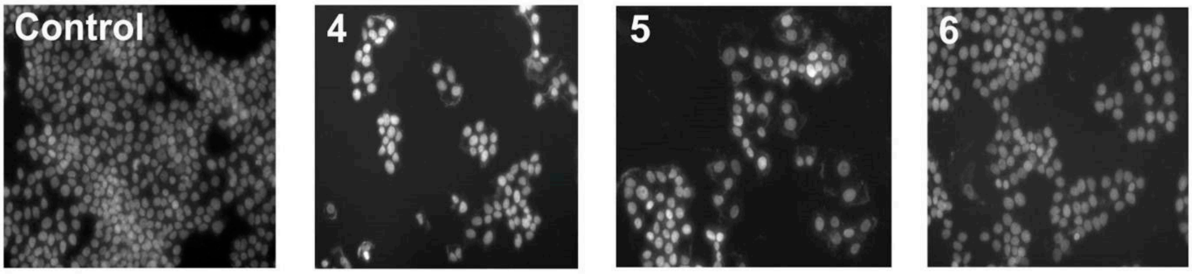

Figure S41. The potential of compounds **4**–**6** to induce apoptotic cell death. MCF-7 cells were exposed to IC_{50} doses of compounds **4**, **5** and **6**, and after 60 h the cells were analysed by fluorescent microscopy.

7.2. Cell viability

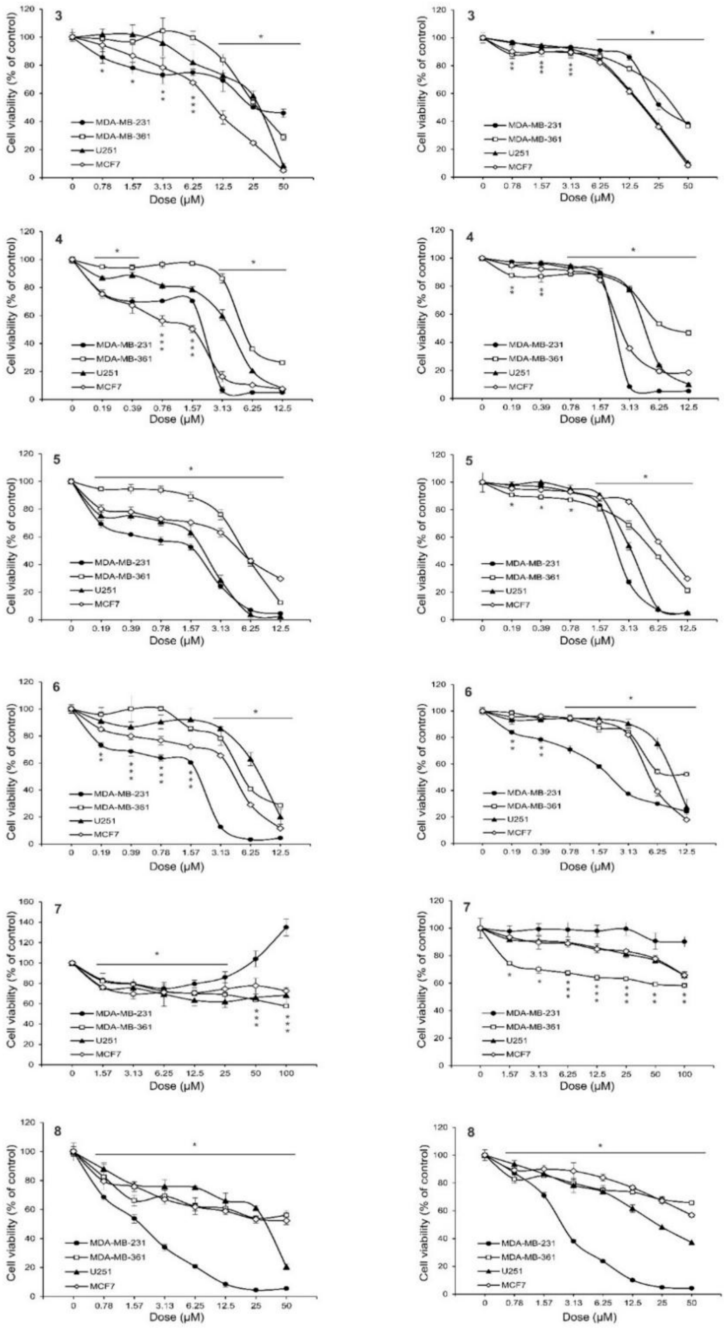

Figure S42. The effect of compounds **3**–**8** on tumour cell viability. Human breast cancer cell lines MCF-7, MDA-MB-361, MDA-MB-231 and the human glioblastoma cell line U251 were treated with **3**–**8** for 72 h and subjected to 3-(4,5-dimethylthiazol-2-yl)-2,5-diphenyltetrazolium bromide (MTT, left) and crystal violet (CV, right) viability assays. The data is expressed as percentage of viability of untreated control (set as 100%) from one representative out of three independent experiments and presented as mean ± SD of triplicate cultures (* $p < 0.05$ in comparison to untreated cells).

References

1. Rikagu Oxford Diffraction. *CrysAlisPro;* Agilent Technologies inc.: Yarnton, Oxfordshire, UK, 2018. https://www.rigaku.com/products/crystallography/crysalis (accessed 02.01.2023).
2. Sheldrick, G. M. SHELXT – Integrated Space-Group and Crystal-Structure Determination. *Acta Crystallogr. A* **2015**, *71*, 3–8. https://doi.org/10.1107/S2053273314026370.
3. Sheldrick, G. M. Crystal Structure Refinement with SHELXL. *Acta Crystallogr. C* **2015**, *71*, 3–8. https://doi.org/10.1107/S2053229614024218.
4. Brandenburg, K. *Diamond*; Crystal Impact GbR: Bonn, Germany, 2022.
5. Schwarze, B.; Jelača, S.; Welcke, L.; Maksimović-Ivanić, D.; Mijatović, S.; Hey-Hawkins, E. 2,2′-Bipyridine-Modified Tamoxifen: A Versatile Vector for Molybdacarboranes. *ChemMedChem* **2019**, *14*, 2075–2083. https://doi.org/10.1002/cmdc.201900554.
6. Carr, N.; Mullica, D. F.; Sappenfield, Eric. L.; Stone, F. G. A. Carborane Complexes of Nickel and Platinum: Synthesis and Protonation Reactions of Anionic Allyl(Carborane) Species. *Inorg. Chem.* **1994**, *33*, 1666–1673. https://doi.org/10.1021/ic00086a017.
7. Warren, L. F.; Hawthorne, M. F. Chemistry of the Bis[π-(3)-1,2-Dicarbollyl] Metalates of Nickel and Palladium. *J. Am. Chem. Soc.* **1970**, *92*, 1157–1173. https://doi.org/10.1021/ja00708a009.
8. Fallis, K. A.; Mullica, D. F.; Sappenfield, E. L.; Stone, F. G. A. Synthesis of Carborane Palladium Complexes: Examples of Low-Temperature Polytopal Rearrangements. *Inorg. Chem.* **1994**, *33*, 4927–4933. https://doi.org/10.1021/ic00100a015.
9. Armarego, W. L. F.; Chai, C. L. L. *Purification of Laboratory Chemicals*; Butterworth-Heinemann, 2003.
10. Kohn, W. Density Functional Theory: Basic Results and Some Observations. In *Density Functional Methods In Physics*; Dreizler, R. M., da Providência, J., Eds.; NATO ASI Series; Springer US: Boston, MA, 1985; pp 1–9. https://doi.org/10.1007/978-1-4757-0818-9_1.
11. Neese, F. The ORCA Program System. *Wiley Interdiscip. Rev. Comput. Mol. Sci.* **2012**, *2*, 73–78. https://doi.org/10.1002/wcms.81.
12. Cramer, C. J.; Truhlar, D. G. Density Functional Theory for Transition Metals and Transition Metal Chemistry. *Phys. Chem. Chem. Phys.* **2009**, *11*, 10757–10816. https://doi.org/10.1039/B907148B.
13. Weymuth, T.; Couzijn, E. P. A.; Chen, P.; Reiher, M. New Benchmark Set of Transition-Metal Coordination Reactions for the Assessment of Density Functionals. *J. Chem. Theory Comput.* **2014**, *10*, 3092–3103. https://doi.org/10.1021/ct500248h.
14. Schröder, H.; Creon, A.; Schwabe, T. Reformulation of the D3(Becke–Johnson) Dispersion Correction without Resorting to Higher than C6 Dispersion Coefficients. *J. Chem. Theory Comput.* **2015**, *11*, 3163–3170. https://doi.org/10.1021/acs.jctc.5b00400.
15. Kossmann, S.; Neese, F. Efficient Structure Optimization with Second-Order Many-Body Perturbation Theory: The RIJCOSX-MP2 Method. *J. Chem. Theory Comput.* **2010**, *6*, 2325–2338. https://doi.org/10.1021/ct100199k.
16. Hellweg, A.; Rappoport, D. Development of New Auxiliary Basis Functions of the Karlsruhe Segmented Contracted Basis Sets Including Diffuse Basis Functions (Def2-SVPD, Def2-TZVPPD, and Def2-QVPPD) for RI-MP2 and RI-CC Calculations. *Phys. Chem. Chem. Phys.* **2014**, *17*, 1010–1017. https://doi.org/10.1039/C4CP04286G.
17. van Lenthe, E.; Snijders, J. G.; Baerends, E. J. The Zero-order Regular Approximation for Relativistic Effects: The Effect of Spin–Orbit Coupling in Closed Shell Molecules. *J. Chem. Phys.* **1996**, *105*, 6505–6516. https://doi.org/10.1063/1.472460.
18. Bader, R. F. W.; Nguyen-Dang, T. T. Quantum Theory of Atoms in Molecules–Dalton Revisited. In *Advances in Quantum Chemistry*; Löwdin, P.-O., Ed.; Academic Press, 1981; Vol. 14, pp 63–124. https://doi.org/10.1016/S0065-3276(08)60326-3.
19. Hilal, R.; Aziz, S. G.; Alyoubi, A. O.; Elroby, S. Quantum Topology of the Charge Density of Chemical Bonds. QTAIM Analysis of the C-Br and O-Br Bonds. *Procedia Comput. Sci.* **2015**, *51*, 1872–1877. https://doi.org/10.1016/j.procs.2015.05.423.

20. Bader, R. F. W. A Bond Path: A Universal Indicator of Bonded Interactions. *J. Phys. Chem. A* **1998**, *102*, 7314–7323. https://doi.org/10.1021/jp981794v.
21. Dmitry V. Korabel'nikov; Zhuravlev, Y. N. The Nature of the Chemical Bond in Oxyanionic Crystals Based on QTAIM Topological Analysis of Electron Densities. *RSC Adv.* **2019**, *9*, 12020–12033. https://doi.org/10.1039/C9RA01403A.
22. Remya, P. R.; Suresh, C. H. Planar Tetracoordinate Carbon in Tungstenacyclobutadiene from Alkyne Metathesis and Expanded Structures. *Dalton Trans.* **2016**, *45*, 1769–1778. https://doi.org/10.1039/C5DT03922C.
23. Shiau, A. K.; Barstad, D.; Loria, P. M.; Cheng, L.; Kushner, P. J.; Agard, D. A.; Greene, G. L. The Structural Basis of Estrogen Receptor/Coactivator Recognition and the Antagonism of This Interaction by Tamoxifen. *Cell* **1998**, *95*, 927–937. https://doi.org/10.1016/S0092-8674(00)81717-1.
24. Word, J. M.; Lovell, S. C.; Richardson, J. S.; Richardson, D. C. Asparagine and Glutamine: Using Hydrogen Atom Contacts in the Choice of Side-Chain Amide Orientation. *J. Mol. Biol.* **1999**, *285*, 1735–1747. https://doi.org/10.1006/jmbi.1998.2401.
25. Brooks, B. R.; Brooks III, C. L.; Mackerell Jr., A. D.; Nilsson, L.; Petrella, R. J.; Roux, B.; Won, Y.; Archontis, G.; Bartels, C.; Boresch, S.; Caflisch, A.; Caves, L.; Cui, Q.; Dinner, A. R.; Feig, M.; Fischer, S.; Gao, J.; Hodoscek, M.; Im, W.; Kuczera, K.; Lazaridis, T.; Ma, J.; Ovchinnikov, V.; Paci, E.; Pastor, R. W.; Post, C. B.; Pu, J. Z.; Schaefer, M.; Tidor, B.; Venable, R. M.; Woodcock, H. L.; Wu, X.; Yang, W.; York, D. M.; Karplus, M. CHARMM: The Biomolecular Simulation Program. *J. Comput. Chem.* **2009**, *30*, 1545–1614. https://doi.org/10.1002/jcc.21287.
26. Phillips, J. C.; Hardy, D. J.; Maia, J. D. C.; Stone, J. E.; Ribeiro, J. V.; Bernardi, R. C.; Buch, R.; Fiorin, G.; Hénin, J.; Jiang, W.; McGreevy, R.; Melo, M. C. R.; Radak, B. K.; Skeel, R. D.; Singharoy, A.; Wang, Y.; Roux, B.; Aksimentiev, A.; Luthey-Schulten, Z.; Kalé, L. V.; Schulten, K.; Chipot, C.; Tajkhorshid, E. Scalable Molecular Dynamics on CPU and GPU Architectures with NAMD. *J. Chem. Phys.* **2020**, *153*, 044130. https://doi.org/10.1063/5.0014475.
27. Shang, X.; Kröger, M. Time Correlation Functions of Equilibrium and Nonequilibrium Langevin Dynamics: Derivations and Numerics Using Random Numbers. *SIAM Rev.* **2020**, *62*, 901–935. https://doi.org/10.1137/19M1255471.
28. de Souza, O. N.; Ornstein, R. L. Effect of Periodic Box Size on Aqueous Molecular Dynamics Simulation of a DNA Dodecamer with Particle-Mesh Ewald Method. *Biophys. J.* **1997**, *72*, 2395–2397. https://doi.org/10.1016/S0006-3495(97)78884-2.
29. Morris, G. M.; Huey, R.; Lindstrom, W.; Sanner, M. F.; Belew, R. K.; Goodsell, D. S.; Olson, A. J. AutoDock4 and AutoDockTools4: Automated Docking with Selective Receptor Flexibility. *J. Comput. Chem.* **2009**, *30*, 2785–2791. https://doi.org/10.1002/jcc.21256.
30. Morris, G. M.; Goodsell, D. S.; Halliday, R. S.; Huey, R.; Hart, W. E.; Belew, R. K.; Olson, A. J. Automated Docking Using a Lamarckian Genetic Algorithm and an Empirical Binding Free Energy Function. *J. Comput. Chem.* **1998**, *19*, 1639–1662. https://doi.org/10.1002/(SICI)1096-987X(19981115)19:14<1639::AID-JCC10>3.0.CO;2-B.
31. Dauvois, S.; Danielian, P. S.; White, R.; Parker, M. G. Antiestrogen ICI 164,384 Reduces Cellular Estrogen Receptor Content by Increasing Its Turnover. *Proc. Natl. Acad. Sci. U.S.A.* **1992**, *89*, 4037–4041. https://doi.org/10.1073/pnas.89.9.4037.
32. Berry, M.; Metzger, D.; Chambon, P. Role of the Two Activating Domains of the Oestrogen Receptor in the Cell-Type and Promoter-Context Dependent Agonistic Activity of the Anti-Oestrogen 4-Hydroxytamoxifen. *EMBO J.* **1990**, *9*, 2811–2818.
